# Epiphytic leafy liverworts diversified in angiosperm-dominated forests

**DOI:** 10.1038/srep05974

**Published:** 2014-08-07

**Authors:** Kathrin Feldberg, Harald Schneider, Tanja Stadler, Alfons Schäfer-Verwimp, Alexander R. Schmidt, Jochen Heinrichs

**Affiliations:** 1 Systematische Botanik und Mykologie, Ludwig-Maximilians-Universität München, 80638 München, Germany; 2 Department of Life Sciences, Natural History Museum, London SW75BD, United Kingdom; 3 Department of Biosystems Science & Engineering, ETH Zürich, 4058 Basel, Switzerland; 4 Mittlere Letten 11, 88634 Herdwangen-Schönach, Germany; 5 Courant Research Centre Geobiology, Georg-August-Universität Göttingen, 37077 Göttingen, Germany

## Abstract

Recent studies have provided evidence for pulses in the diversification of angiosperms, ferns, gymnosperms, and mosses as well as various groups of animals during the Cretaceous revolution of terrestrial ecosystems. However, evidence for such pulses has not been reported so far for liverworts. Here we provide new insight into liverwort evolution by integrating a comprehensive molecular dataset with a set of 20 fossil age constraints. We found evidence for a relative constant diversification rate of generalistic liverworts (Jungermanniales) since the Palaeozoic, whereas epiphytic liverworts (Porellales) show a sudden increase of lineage accumulation in the Cretaceous. This difference is likely caused by the pronounced response of Porellales to the ecological opportunities provided by humid, megathermal forests, which were increasingly available as a result of the rise of the angiosperms.

In recent years, the hypothesis of the Cretaceous Terrestrial Revolution (KTR) was introduced in response to the increasing accumulation of evidence for a substantial change in the terrestrial biodiversity around 110 to 70 million years ago (Ma)^1^,^2^. The KTR is marked by the fact that terrestrial diversity exceeded the diversity of marine habitats for the first time^3^ and that it coincided with the rapid rise of the angiosperms^3^,^4^,^5^,^6^,^7^,^8^,^9^,^10^. Evidence for coinciding Cretaceous pulses of increased diversification have been found in many lineages of terrestrial organisms including various lineages of land plants^11^,^12^,^13^, insects^14^,^15^,^16^,^17^, and tetrapods^1^,^18^. In turn, the coincidence of KTR and rise of the angiosperms implies some correlation of these events, although the causality is currently unknown. However, this argument is consistent with the reports on the coincidences of changes in the “tree of life” and the rise of the angiosperms. Examples of coinciding diversification include those observed in ants^14^, and “derived ferns”^11^ as well as changes in the lineage composition of dinosaurs^19^.

Further support of a causal link between the rise of angiosperms and the KTR was provided by ecophysiological studies providing evidence for a connection of several traits of angiosperms and the establishment of humid megathermal forests^2^,^20^,^21^,^22^,^23^,^24^. This scenario challenged the hypothesis of the replacement of lineages considered as primitive such as bryophytes, ferns, and gymnosperms by angiosperms as predicted by the sequential replacement hypothesis^4^,^25^. In contrast, a new hypothesis emerged in which various lineages of organisms underwent increased diversification during the KTR and early Cenozoic in response to the rapid spread of angiosperm-dominated habitats^9^,^10^.

In contrast to other land plants, no evidence has been found for pulses in the diversification of liverworts that coincide with the KTR or post-KTR events^12^,^26^,^27^,^28^. This is remarkable given the evidence for KTR and post-KTR pulses of diversification in nearly all other groups of land plants and the ecological association of many liverwort species and angiosperms^12^,^29^,^30^. Previous studies reported either constant or declining diversification rates, indicating that liverworts are either in an equilibrium state or are actually in decline as expected under the replacement hypothesis. However, these constant rates may be an artefact because analyses of diversification rates are highly sensitive to sampling density^31^. These arguments resemble to some degree the controversy about the impact of the Cretaceous-Palaeogene mass-extinction event on land-plant diversity^32^. High-resolution regional samplings were required to detect the impact of mass extinctions on plant diversity^32^,^33^. In turn, these arguments suggest the need for a high-resolution study that focuses on the unbalanced distribution of species diversity across liverwort phylogeny and the high proportion of epiphytes in some lineages.

Here, we explore the hypothesis that epiphytic liverworts diversified in the shadow of angiosperms while generalist liverworts were less affected by the KTR. The expectation of an increased speciation rate of epiphytic liverworts is supported by the argument that the rise of angiosperms increased the occurrence of humid forests^20^. The vast majority of extant epiphytic liverworts are found in rainforests dominated by angiosperms, which suggests a close link between angiosperms and epiphytic liverworts. This hypothesis is also supported by evidence from other studies of a causal link between angiosperm forests and epiphytism in ferns^34^, and the connection between leaf-shape evolution of southern hemisphere conifers and angiosperms^35^. To address this hypothesis, we selected the Palaeozoic sister orders Jungermanniales and Porellales^28^ because of their high species diversity, of about 2,600 and 2,300 species respectively, and the substantial difference between them in the proportion of epiphytic species. The majority of Porellales species grow either epiphytically or epiphyllically, whereas Jungermanniales show a dominance of species with a more generalistic strategy. Based on evidence provided in ecophysiological studies on early angiosperms, we expected the majority of epiphytic and epiphyllic liverworts to have diversified either during the KTR or after the Cretaceous-Palaeogene (K-Pg) boundary and, in contrast, generalists to have accumulated their diversity under a rather constant diversification rate as inferred in preceding studies^12^,^27^. Specifically, we explore this hypothesis by examining the evidence supporting two pertinent arguments: 1) that the two orders show distinct patterns of diversification during the Mesozoic and early Cenozoic; 2) that these differences coincide with the ecological preferences of the majority of species belonging to each order, in particular the strong preference in Porellales for epiphytic habitats.

## Results

### Lineage diversification

**Figure 1:**
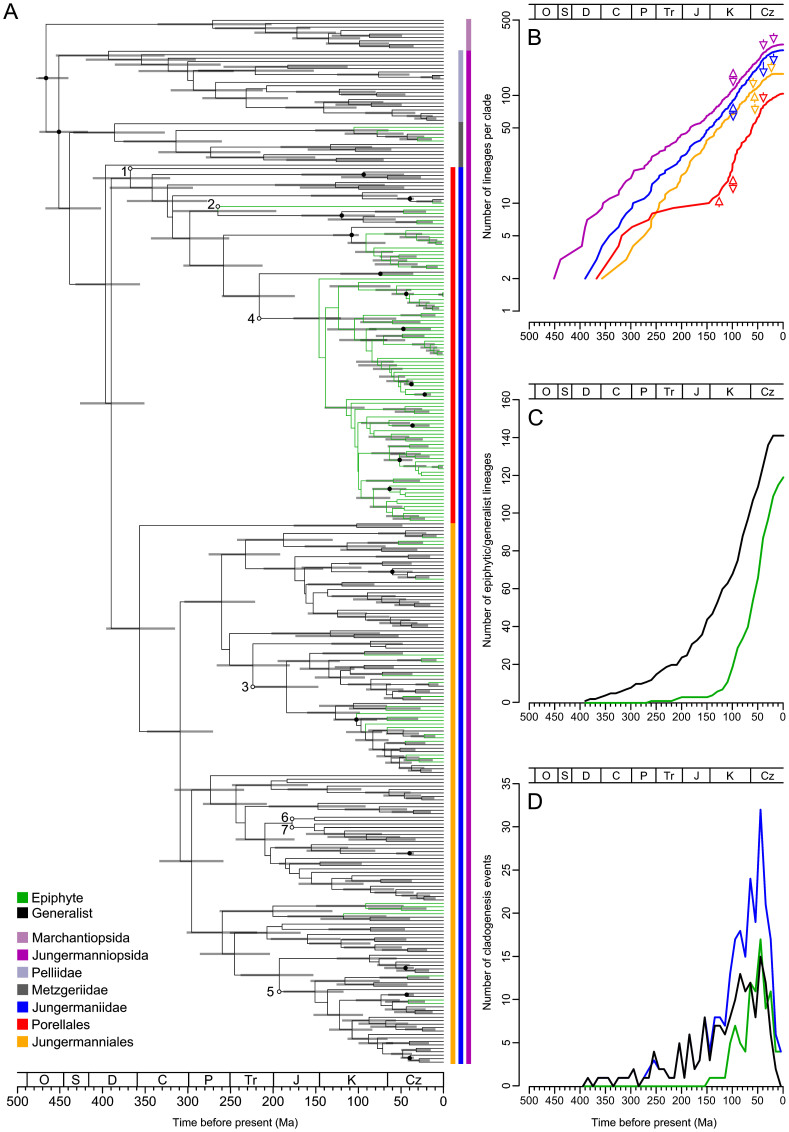
Diversification of liverworts. (*A*) Mean age consensus chronogam with time scale. Green branches are epiphytes, black branches are generalists. Grey horizontal bars show confidence intervals. Black dots indicate nodes with calibrations (see Supplementary 2.1), white dots with small letters indicate the shifts in the diversification rates (breakpoints 1 to 7 estimated by the MEDUSA analysis, see Supplementary 2.7). Vertical bars indicate higher taxonomic units marked using the colour code shown at the lower left corner of the figure. We employed the current classification differentiating into classes (-opsida), subclasses (-idae), and orders (-ales). (*B*) Lineage through time plot for the Jungermanniopsida (purple), Jungermanniidae (blue), Jungermanniales (orange), Porellales (red). Arrows indicate rate shifts as estimated with LASER (Supplementary 2.6) with rate decline indicated by arrows pointing downwards and rate incline by arrows pointing upwards. (*C*) Accumulation of epiphytic and generalist species diversity through time (green epiphytes, black generalists). (*D*) Number of cladogenesis events through time for epiphytes (green), generalists (black), both (blue).

The orders Jungermanniales and Porellales show distinct patterns of biodiversity assembly through the Mesozoic and early Cenozoic (Figs 1A–1D, Supplementary 2.3–2.7). Jungermanniales accumulated their diversity with a relatively constant diversification rate until about 20–40 Ma, whereas Porellales show a delay of the accumulation of lineages from the Permian-Triassic border onwards followed by a sudden increase in the Cretaceous (Fig. 1B, Supplementary 2.4). A decline of the diversification rate from ca. 20–40 Ma onwards is indicated for both orders (Fig. 1B, Supplementary 2.3). This is consistent with the negative gamma statistics of constant diversity accumulation over time with -5.98 for Jungermanniales and -2.12 for Porellales (Supplementary 2.4). Compared to Jungermanniales, the Porellales show not only evidence for the shared reduction of the diversification rate around 40 Ma but also an evident increase of the diversification from the mid Cretaceous to the early Cenozoic (Fig. 1B, Supplementary 2.5).

Evidence was found for statistically significant changes in the diversification rates of leafy liverworts (Fig. 1B, Supplementary 2.6 and 2.7). Diversification models assuming non-constant rates fitted better with the recovered phylogeny and main clades recognised (Suppementary 2.6), and up to seven significant shifts in the diversification rates were recovered in the MEDUSA analyses (Fig. 1A, Supplementary 2.7). Three breakpoints with rate slow-downs were associated with nodes containing rather few extant species (Fig. 1A, Supplementary 2.7; breakpoints 1, 2 & 6), whereas two out of the four rate changes indicating increased rates were associated with the two most species rich families of liverworts, the Lejeuneaceae and Plagiochilaceae (Fig. 1A, Supplementary 2.7; breakpoints 3, 4 & 5). The results suggested an increased rate during the KTR associated with the onset of the diversification of species-rich families in Porellales such as Lejeuneaceae and Frullaniaceae (Fig. 1B, Supplementary 2.3–2.8). These results are consistent with the estimates of birth-death rates obtained using DIVERSITREE (Supplementary 2.9), which indicate a slightly higher speciation rate for Porellales (λ = 0.0120) compared to Jungermanniales (λ = 0.0093) in the Bayesian mcmc estimation with the extinction rate (µ) to be close to zero.

### Differences in diversification between epiphytes and generalists

A much larger fraction of Porellales grows preferably as epiphytes, with > 95% in Porellales versus < 30% in Jungermanniales. BiSSE analyses with DIVERSITREE found evidence supporting the hypothesis of interdependence of epiphytism and diversification rates (Table 1). A diversification rate of λ_E_ = 0.08906 for epiphytes and λ_G_ = 0.07725 for generalists was found with the six parameter model; the other models likewise indicated higher diversification rates of epiphytes compared to generalists (Table 1, Supplementary 2.8). The selected models suggested a significant (p < 0.005 in Table 1) interdependence of the diversification rates and preference scored as epiphyte or generalist combined with the asymmetric transition between the two preference states. The loss of epiphytism was found to be more frequent than the gain of epiphytism (model with six parameters, loss q_E-G_ = 0.008 vs. gain q_G-E_ = 0.0006 with a significance of p < 0.005; Supplementary 2.8 and 2.9). This is consistent with the observation that more than 95% of the putative transitions to epiphytism were younger than 150 million years (Fig. 1C). More importantly, the number of cladogenesis events per 10 million years (Fig. 1D) showed a substantially higher increase during the KTR compared to the Late Jurassic and Early Cretaceous in epiphytes (x5.7) than in generalists (x1.8). Notably, 53% of cladogenesis events involving epiphytes happened in the KTR (110-70 Ma) and 70% in the period from 110 to 30 Ma. In contrast, conspicuously smaller proportions, 25% and 53% respectively, contributed to the total of cladogenesis events associated with generalists.

## Discussion

Our analyses point to remarkable differences in the diversification rates of the predominantly epiphytic Porellales and the predominantly generalistic Jungermanniales. Porellales show a sudden increase of lineage accumulation in the Cretaceous, whereas Jungermanniales had a relatively constant diversification rate since the Palaeozoic. The declining accumulation of both lineages around 40 Ma in the LTTPs (Fig. 1B, Supplementary 2.3) and the TreePAR analyses (Supplementary 2.5) is very likely the result of the density of the taxon sampling, but the differences between Porellales and Jungermanniales in the LTTPs, gamma values, and TreePAR analyses (Supplementary 2.3–2.5) are unlikely to be the result of unbalanced sampling, because we sampled ~5% of the extant diversity of each order. Within each order, taxa within families were sampled randomly under the constraint of the proportional contribution of families to the taxon diversity of the order. The low transition rates from a generalistic to an epiphytic mode of life estimated in the BiSSE analysis (Table 1, Supplementary 2.8) indicate that the delayed diversification of the Porellales crown group may be caused by their preference for epiphytic niches. This argument is consistent with the results of diversification models recovering slightly lower extinction rates and higher speciation rates for Porellales than those estimated for Jungermanniales. The divergence time estimated for the rise of the Porellales suggests a coincidence of increased accumulation of epiphytic diversity with the rise of the angiosperms.

The results of the LTTPs and the BiSSE analyses raise the issue of why epiphytic liverworts appear to be more sensitive to the transformation of the environment caused by angiosperms, compared to generalists. The latter may show a higher ecological flexibility and thus they may be able to colonize new habitats or tolerate local environmental changes without a significant change in the rate of diversification, meaning that extinction and speciation events are balanced. Some of these taxa are also able to grow epiphytically, but they are not restricted to this habitat. In contrast, species belonging to epiphytic lineages exhibit a marked preference for forests with high humidity. A considerable portion of Porellales shows a unique preference for the surface of the leaves of vascular plants. Although these epiphylls are not restricted to growing on angiosperms and are also found on the leaves of other land plants such as ferns, epiphyllic species are nested in clades that diversified not earlier than the Late Cretaceous^26^. In general, there is no strict host preference for epiphytic liverworts^36^,^37^,^38^ and substrate preferences are likely shaped by humidity of the environment with a strong tendency for epiphytic growth in superhumid forests^39^.

The new results on the diversification of liverworts provide further support for the hypothesis that various groups of land plants exploited the opportunities created by the rise of the angiosperms^11^. In turn, our study provides further evidence for the rise of the angiosperms as the putative trigger of the KTR. Specifically, the opportunities for leafy liverworts were mainly provided by the expansion of epiphytic and epiphyllic habitats offered by humid, megathermal forests dominated by angiosperms. This resembles the situation of ferns in humid, megathermal forests^11^ but the Cretaceous fern diversification was likely mainly due to ferns exploiting terrestrial habitats. The majority of epiphytic fern lineages, on the other hand, shows a post K-Pg onset of their diversification^34^. Nonetheless, some epiphytic filmy ferns were found to diversify as early as the KTR^40^. This difference may be explained by various factors, including pre-adaptation to epiphytic habitats in leafy liverworts and filmy ferns compared to other epiphytic fern groups, and by the higher tolerance of epiphytic liverworts to disturbance, e.g. the K-Pg mass extinction. The dissimilar accumulation of liverwort and fern epiphyte diversity coincided with the phases of vein density increase reported for angiosperms in the mid-Cretaceous and after the K-Pg boundary^21^.

In this context, it is important to note that some controversy still exists concerning the origin of the first forests dominated by angiosperms. Based on Cretaceous North American floras, angiosperm-dominated forests were assumed to be not older than K-Pg. However, increasing evidence has accumulated suggesting that angiosperms may have formed a substantial component of the Late Cretaceous forest vegetation in at least some areas^10^,^41^. Arguments supporting the pre-Cenozoic existence of angiosperm-dominated megathermal forests were also provided in studies focusing on tropical angiosperm families such as the custard apple family (Annonaceae^42^), palms (Arecaceae^43^), and rosids^5^.

In summary, we found evidence for distinct responses of leafy liverwort clades to the opportunities and challenges provided by the rise of the angiosperms. Ecological generalists may have exploited these opportunities without changes in the speciation rates, whereas epiphytic specialists show a strong increase in the diversification rate coinciding with the rise of the angiosperms. In turn, the diversification of ecological generalists appears not to be affected by the great mass-extinction events, e.g. at the Permian-Triassic, Triassic-Jurassic, and Cretaceous-Palaeogene boundaries or by the rise of the angiosperms. For Porellales, the increase of the speciation rate was found to be correlated with epiphytism and thus the evolutionary success of the order is likely closely linked to the establishment of humid forests dominated by angiosperms. We also considered the alternative that the low diversification rate in the Triassic and Jurassic is an artefact of our study having been based on extant taxa. It is possible that epiphytic diversity occurring mainly on Mesozoic gymnosperms was replaced by epiphytic diversity occurring mainly on angiosperms. However, no evidence exists to support the assumed lineage-specificity of epiphytic liverworts to either angiosperms or gymnosperms. Instead, the humidity maintained in forests is the most probable factor controlling the assembly of epiphytic liverwort diversity.

## Methods

### Taxon sampling

In total, we sampled 303 species of liverworts with the two orders Porellales and Jungermanniales sampled proportionally to represent about 5% of their extant species diversity. We also sampled representatives of the Metzgeriidae and Pelliidae (Supplementary Information 4: Accession numbers). The sampling was designed based on our current understanding of the phylogeny and the systematics of these liverworts^44^. Species numbers were compiled based on newly obtained estimates and taking into account evidence for a higher number of currently unrecognized species in Porellales versus Jungermanniales^45^,^46^. The obtained sampling is best described as random but constrained by our current knowledge of the phylogeny and distribution of species diversity. Several analyses were specifically applied to determine the impact of the sampling density and species number estimates, e.g. MEDUSA. The design of the dataset took into consideration challenges such as limited sampling by carrying out analyses allowing to correct species numbers and by employing a random sampling within well-defined clades.

### Divergence time estimates

Bayesian divergence time estimates were generated with the software package BEAST 1.6.2^47^,^48^ using a dataset including 303 species and sequences of the plastid gene *rbc*L. Twenty fossil calibrations were selected and assigned based on recent research (Supplementary 1.2). The assignment of these calibrations was based on our current understanding of the phylogeny and taxonomy of the related liverwort clades whereas the assigned age intervals were based on the recent literature on the topic (Supplementary 1.2.1 and 1.2.2). Age intervals were integrated as age constraints with a uniform prior distribution for the minimum age of the fossil and a maximum age of the root (475 Ma). The latter accounts for uncertainties of maximum clade age due to the relative sparseness of fossils older than 35 Ma^49^,^50^. The maximum age of liverworts was set to 475 Ma based on the age of the oldest known cryptospores^51^,^52^. Final estimates were obtained using the GTR+I+G model as determined with jModeltest 1.0^53^, lognormal relaxed molecular clock^48^, and a birth-death tree model^54^. The analysis was run for 200,000,000 generations and by sampling every 20,000^th^ tree, so that the resulting tree file contained 10,000 trees. Effective sample size was investigated with dedicated tools such as TRACER 1.5 that are part of the BEAST package. After a burn-in of 25% a maximum credibility tree was compiled with TreeAnnotator 1.6.2. Phylogenetic uncertainty, e.g. node robustness and branch length, were taken into account by employing methods using at least 100 randomly selected chronograms generated in the BEAST analyses instead of the single maximum credibility tree (Supplementary 2.1–2.4). We carefully explored the distribution of substitution rate changes to detect biases such as incorrectly applied time constraints and correlation between diversification rates and substitution rates. No significant correlation was found.

### Diversification rate analyses

These analyses were carried out with 100 randomly selected chronograms or with the maximum credibility tree from the BEAST results for Jungermanniopsida, Jungermanniidae, Jungermanniales, and Porellales (for details see Supplementary Information 1: Material and Methods). Lineage through time plots (LTTPs) were generated with TreeSIM^55^, and the gamma statistic values^56^ were calculated in APE^57^ while the null distribution of the gamma statistic was sampled with GEIGER^58^. Evidence for non-constant rates was further investigated in LASER^59^ by testing the fit of several time-dependent models (assuming constant rates or variable rates). The results of these analyses were investigated using the MEDUSA approach as implemented in GEIGER^58^. TreePAR analyses^60^ were carried out to detect changes in the diversification rates through time. A penalized likelihood analysis was performed with the chronoplot function in APE^57^ to visualize changes in the substitution rates across the chronograms. The analysis was performed for nine values of the smoothing parameter lambda, and with the cross-validation option on (Supplementary 2.2).

### Diversification rate and ecological preference

The ecological preference of the species included was inferred based on reports in the literature, our own observations, and herbarium specimens. Two distinct conditions were found: 1) Generalists show a wide range of preferences including terrestrial, saxicolous, and sometimes epiphytic habitats. However, they rarely show a strong preference for a certain substrate. The few epiphyte lineages of Jungermanniales do not show special morphological adaptations such as complicate bilobed leaves with the ventral lobes modified into pockets or watersacs; 2) Epiphytic taxa, in contrast, grow exclusively or nearly exclusively on the branches, stems or leaves of vascular plants and have adapted morphologically to this habitat by developing the above-mentioned types of leaves, bundled rhizoids, and exclusively lateral branching that allows a flat growth of the shoots pressed to their substrate^61^.

These data were used in BiSSE analyses^62^ calculated in DIVERSITREE^63^ (for details see Supplementary 1.1.8). The distribution of cladogenesis events associated with both classes was inferred by counting the number of events per time unit of 10 Ma for the whole chronogram or for three age classes of 40 Ma each, pre KTA (150-110), KTA (110-70), post-KTA (70-30). All analyses were designed and carried out considering the known challenges of BiSSE analyses^62^,^64^,^65^,^66^. Several analyses were employed to explore the impact of the taxon sampling and phylogenetic uncertainty to the obtained results (gamma statistics, Supplementary 2.4; mcmc-cross-validation of the birth-death model, Supplementary 2.9).

## Author Contributions

K.F., H.S. and J.H. designed the research and wrote the paper; K.F., A.S.-V., A.R.S. and J.H. assembled the data sets; K.F. analyzed the data; T.S. assisted in the TreePAR-analyses.

## Supplementary Material

Supplementary Information Supplementary Information

## Figures and Tables

**Table 1:** Results of the BiSSE analyses exploring the interdependence of diversification rate on ecological preference (epiphytic or generalist). Seven models were tested and compared using likelihood values (ln) and Akaike Information Criterion (AIC). The significance of the differences between models was explored using a Χ^2^ test. The seven models considered the following parameters: speciation rate (λ), extinction rate (µ), and character state transition rate (q). These models parameters were either treated as dependent on the ecological preferences (≠) or as constant ( = ). The upper three models were found to be the best fit using a Χ^2^ test and significance value of p < 0.01. The three selected models shared the prediction of the interdependence of character transition (q) and the two parameters (λ and µ) contributing to the diversification rate

parameters	model	ln	AIC	X^2^ values	p value
6	λ_0_≠λ_1_,μ_0_≠μ_1_,q_G-E_≠q_E-G_	−1729.0	3469.9		
5	λ_0_ = λ_1_,μ_0_≠μ_1_,q_G-E_≠q_E-G_	−1729.2	3468.4	0.469	0.493505
	λ_0_≠λ_1_,μ_0_ = μ_1_,q_G-E_≠q_E-G_	−1729.0	3468.0	0.051	0.821696
	λ_0_≠λ_1_,μ_0_≠μ_1_,q_G-E_ = q_E-G_	−1732.7	3475.4	7.521	0.006097
4	λ_0_ = λ_1_,μ_0_ = μ_1_,q_G-E_≠q_E-G_	−1750.2	3508.4	42.501	5.901e-10
	λ_0_≠λ_1_,μ_0_ = μ_1_,q_G-E_ = q_E-G_	−1733.7	3475.4	9.462	0.008816
	λ_0_ = λ_1_,μ_0_≠μ_1_,q_G-E_ = q_E-G_	−1735.4	3478.8	12.852	0.001619
