# Supplementary material for: Epiphytic leafy liverworts diversified in angiosperm-dominated forests
Source: Sci Rep. 2014 Aug 7;4:5974. doi: 10.1038/srep05974 (PMC4124468; doi:10.1038/srep05974)
Supplement: Supplementary Information [file srep05974-s1.pdf]

# Epiphytic leafy liverworts diversified in angiosperm-dominated forests

Kathrin Feldberg, Harald Schneider, Tanja Stadler, Alfons Schäfer-Verwimp, Alexander R. Schmidt, and Jochen Heinrichs

## Supplementary Information

**Supplementary Information 1: Material and Methods**
1.1. Analyses with R 2
1.1.1. General remarks 2
1.1.2. Penalized likelihood (Chronoplots with APE) 2
1.1.3. Lineages through time plots with TreeSIM 2
1.1.4. Gamma statistics with APE and MCCR test with GEIGER 2
1.1.5. Estimation of diversification rate shifts with TreePAR 3
1.1.6. Modelfitting with LASER 3
1.1.7. MEDUSA 3
1.1.8. BiSSE with DIVERSITREE 3
1.1.9. Estimation of speciation and extinction rates with DIVERSITREE 4
1.2. Fossil calibrations 5
1.2.1. General remarks 5
1.2.2. Discussion of fossil assignments 6
**Supplementary Information 2: Results**
2.1. Chronogram for Jungermanniopsida (estimated with BEAST 1.6.2.) 9
2.2. Penalized likelihood (Chronoplots with APE) 17
2.3. Lineages through time plots with TreeSIM 23
2.4. Gamma statistics with APE and MCCR test with GEIGER 24
2.5. Estimation of diversification rate shifts with TreePAR 25
2.6. Modelfitting with LASER 26
2.7. MEDUSA 28
2.8. BiSSE with DIVERSITREE 29
2.9. Estimation of speciation and extinction rates with DIVERSITREE 35
**Supplementary Information 3: References** 36
**Supplementary Information 4: Accession Numbers** 38

# Supplementary Information 1: Material and Methods

## 1.1 Analyses with R

The following analyses were performed with R[1] (http://www.r-project.org/).

### 1.1.1. General remarks

A dated phylogenetic framework was reconstructed by estimating simultaneously divergence time estimates and topology using a bayesian divergence time estimate approach as implemented in Beast 1.6.2. The analysed dataset comprised representatives of the leafy and simple thalloid liverworts (class Jungermanniopsida) plus a few representatives of the complex thalloid liverworts (class Marchantiopsida) utilized as outgroup taxa. A taxonomical overview of liverworts including thorough morphological discussion can be found in[2]. The sampling was designed to obtain a proportional sampling of the two orders Jungermanniales and Porellales via random sampling within clades that are known to be phylogenetically robust. Except for 1.1.7 and 1.1.8, four independent analyses were run with the sampling corresponding to the hierachical classification: the class Jungermanniopsida (simple thalloid and leafy liverworts), the subclass Jungermanniidae (leafy liverworts), as well as the orders Porellales (mainly epiphytic leafy liverworts) and Jungermanniales (mainly generalistic leafy liverworts). This was done to investigate whether the results for Jungermanniopsida (comprising all investigated taxa) were challenged if the subclades were analysed separately (subsampling effects).
For analyses 1.1.2, 1.1.4, 1.1.6, 1.1.7, 1.1.8, and 1.1.9 the maximum credibility tree obtained from the Beast analyses was used. For analyses 1.1.3 and 1.1.5, 100 trees were selected randomly from the trees saved in the convergence phase of the Beast analysis used to estimate the maximum credibility tree. These analyses allowed us to explore the influence of phylogenetic uncertainty to our investigation. The assignments of the fossils are discussed in section 1.2.1.

### 1.1.2. Penalized Likelihood (Chronoplots with APE)

This method was applied to explore the substitution rate changes and the influence of the fossil assignments on our investigation. In particular, we investigated if the studied changes in the diversification rates show correlations with changes in the substitution rates. To visualize rate changes across the maximum credibility tree the penalized likelihood[3] (PL) approach as implemented in the chronoplot function of APE[4] (http://cran.rproject.org/web/packages/ape/index.html) was performed for Jungermanniopsida, Jungermanniidae, Jungermanniales and Porellales. To find the smallest smoothing parameter *lambda* that allows the maximal variation of the substitution rates in the subsequent PL, the cross-validation option was employed (values for the smoothing parameter *lambda* ranging from 10e-4 to 10e4). Nine values of *lambda* were sampled and plotted against the sum of the cross-validation values, then the analysis was performed with the smallest value for *lambda*[5]. The results for the cross-validation of the four clades are displayed in Supplementary 2.2. Figs 3-6 in Supplementary 2.2 show the rates plotted on the four clades of the maximum credibility tree from the Beast analysis.

### 1.1.3. Lineages through time plots with TreeSIM

To visualize the lineage diversification of the four clades (see 1.1.1) in time with confidence intervals, lineages through time plots (LTTP) were estimated with the R package TreeSIM[6] (http://cran.rproject.org/web/packages/TreeSim/index.html) for 100 random trees from the convergence phase of the Beast analysis. These plots provide a visualisation of the accumulation of extant diversity trough time (shown at the x-axis in million years towards the present (Ma). The y-axis plotted in a logarithmic scale shows the proportion of lineages present at a given time interval. By doing so, we were able to integrate phyologenetic uncertainty (composed by topological uncertainty and variation of branch length estimates).

### 1.1.4. Gamma Statistics with APE and MCCR test with GEIGER

The gamma statistics of Pybus & Harvey[7] is a constant rates (CR) test that evaluates the null hypothesis that speciation ($\lambda$) and extinction ($\mu$) rates are constant through time using the internode intervals in a tree. Given a pure birth process (yule process) and a complete phylogeny the gamma values ($\gamma$) show a normal distribution with a mean zero and a standard deviation 1. The estimated $\gamma$ shows to what extent a given phylogeny differs from this distribution. A negative $\gamma$ ($\gamma < 0$) means that the cladogenic events are distributed more towards the root of the tree than can be expected under a pure birth model, indicating declining diversification rates. Positive

values ($\gamma > 0$) indicate a concentration of cladogenic events towards the tip nodes and therefore an acceleration of diversification. The null hypothesis of a constant rate is rejected when $\gamma < -1.645$ or $\gamma > 1.645$.
$\gamma$ was estimated with the "gammaStat" function of APE[4] for the four clades of the maximum credibility tree (see 1.1.1). To account for the incompleteness of the phylogeny that may have biased the $\gamma$ towards negative values, the null distribution of $\gamma$ is estimated with the MCCR Test[7]. If the observed $\gamma$ falls into the tail of this null distribution even with incomplete taxon sampling, there are more cladogenetic events early or later in the history of the tree than expected under a constant rates model. The birth-death simulation with 500 replicates and random pruning was carried out with GEIGER[8] (http://cran.r-project.org/web/packages/geiger/index.html) for the four clades recognised in the credibility tree (see above).

### 1.1.5. Estimation of diversification rate shifts with TreePAR

The R package TreePAR[9] (http://cran.r-project.org/web/packages/TreePar/index.html) includes functions to estimate and visualize diversification rates shifts for a set of trees. The software was designed to indentify evidence for changes in speciation ($\lambda$) and extinction ($\mu$) rates as well as mass extinction events using not a single tree but evaluating the hypothesis accross several trees generated for the same dataset. The function "bd.shifts.optim", that estimates the maximum likelihood speciation and extinction rates as well as the rate shift times, was run with 100 random trees for the four clades (see 1.1.1). These analyses provide additional evidence for the detected rate changes under consideration of phylogenetic uncertainty measured using 100 randomly sampled trees (see above).

### 1.1.6. Modelfitting with LASER

To test which evolutionary model fits best to the branch times of the maximum credibility tree (see 1.1.1), we used the functions "fitdAICrc" and "yule-n-rate" in implemented LASER[10] (http://cran.r-project.org/web/packages/laser/index.html). The fitdAICrc function fits a set of rate-variable and rate-constant models to the branching times and compares the AIC (Akaike Information Criterion) scores to find the best fitting models. The analysis was run for the following models: pure birth (yule), birth-death, DDL, and DDX. The "pureBirth" function models the speciation rate ($\lambda$) and the "bd" function additionally the extinction rate ($\mu$); both are constant rate models. The diversity density dependend models DDX (taxa dependent) and DDL (rate dependent) were used beside the other models. The "yule-n-rate" function fits models with up to 5 rate changes under a pure birth model to a set of branching times and estimates the time of the rate change, again an AIC score is given. The four clades (see 1.1.1) where analysed separately. The resulting values for all models are displayed in the corresponding results sections. The best model (yule-5-rate) was chosen with the AIC and the times of rate changes where plotted on the lineages through time plots estimated with TreeSIM (see 1.1.3).

### 1.1.7. MEDUSA

With the "medusa" function of the R package GEIGER[8] it is possible to estimate breakpoints in diversification by fitting diversification models (yule, birth-death, mixed) to a phylogeny. This approach allows to correct the impact of the incomplete sampling by considering the estimates of species diversity of terminal nodes. In this case, we used estimates for the species richness of each family of liverworts. To choose the best fitting model the AIC (Akaike Information Criterion) and the AICc (corrected Akaike Information Criterion) were employed. It was necessary to create a matrix of taxonomic richness containing the species numbers of genera or families as well as a reduced tree containing only one representative of the genus or family. With Mesquite[11] (http://mesquiteproject.org) the maximum credibility tree of Jungermanniopsida (see 1.1.1) was reduced to one species per family and a matrix with the species numbers per family was build. The following arguments were employed: AICc and AIC as criterions, "mixed" as model (considers yule and birth-death).

### 1.1.8. BiSSE with DIVERSITREE

To estimate if the characters "epiphytism" and "generalism" have a significant influence on the evolution of species, a BiSSE (binary state speciation and extinction)[12,13] analysis was performed with the R package DIVERSITREE[12] (http://cran.r-project.org/web/packages/diversitree/index.html) for the maximum credibility tree and the clade Jungermanniopsida. The speciation rates ($\lambda$), extinction rates ($\mu$) and character state transition rates (q) for epiphytes (E) and generalists (G) were estimated with the "make.bisse" function with maximum likelihood as well as bayesian inference under an exponential prior distribution[13,14]. We explored in total seven models with one model having six (no constant rates), three models having five (one constant rate), and three models having four parameters (two constant rates). For each model, the likelihood values were used besides

AIC to rank the models hierarchically. Significance was tested using a chi-square test to estimate p-values. Particular care was given to observe the test statistics to identify conflicts. Character dependence of rate changes were also inferred using a sister comparison carried out by a Wilcoxon test. This analysis provided additional support to the hypothesis estimated independently from the BiSSE approach.

### 1.1.9. Estimation of speciation and extinction rates with DIVERSITREE

To explore the robustness of the test statistics, we employed the MCMC-based versions of BiSSE embedded in DIVERSITREE[14]: We calculated the distribution T null of each parameter set considered in the models above under a birth-death process. The constant rate function ("make.bd" function of DIVERSITREE) was employed when applicable. The obtained test statistics was used to explore the significance of the estimated model parameters in combination with the test statistics used to explore the best fitting models.

## 1.2. Fossil calibrations

### 1.2.1. General remarks

Fossils are assigned as most recent common ancestors (mrca) based on comparison with the extant liverwort diversity and phylogenetic evidence. Information on the morphology of the fossils was taken from the literature and/or based on the study of original material including types. In the framework of this study, we investigated some 200 amber inclusions of liverworts including specimens from the American Museum of Natural History in New York (USA), the National Museum of Natural History of the Smithsonian Institution in Washington DC (USA), the Natural History Museum London (UK), the Geoscientific Collections of the Georg August University Göttingen (Germany), the Geological-Palaeontological Collection of the University of Hamburg (Germany), the amber collection of the Museum of Natural History Berlin (Germany), the Stuttgart State Museum of Natural History (Germany), and the private collections of C. Groehn (Glinde, Germany), H. Grabenhorst (Wienhausen, Germany) and C. & H. W. Hoffeins (Hamburg, Germany). Fossils included in the analyses are listed in Supplementary 1.2.1 Table 1 and discussed in Supplementary 1.2.2. Intervals of the age of the fossils were taken from the most recent literature on the particular lagerstaetten. The youngest age was considered as minimum age constraint as recommended by several authors[15-18] whereas the maximum age of 475 Ma for the root of the liverwort tree was employed as the maximum age constraint. This estimate is based on the age of the oldest known land plant fragments, Cambrian cryptospores[19], some of which show similarities with the spores of certain extant liverworts[20].

**Supplementary 1.2.1 Table 1:** Fossils employed as minimum age constraints. The red letters in the first column refer to the chronogram for Jungermanniopsida (Supplementary 2.1 Fig.1).

| | Age assignments | Taxon and reference | Origin and age of fossils |
|---|---|---|---|
| A | uniform: 385.3 – 475 Ma | *Metzgeriothallus sharonae* VanAller Hernick et al. [21] | USA: 385.3-391.8 Ma |
| B | uniform: 35.0 - 385.3 Ma | *Ptilidium* spec. [22, 23] | Baltic amber: 35-55 Ma |
| C | uniform: 35.0 - 385.3 Ma | *Porella subgrandiloba* Grolle & M.L.So[24] | Baltic amber: 35-55 Ma |
| D | uniform: 35.0 - 385.3 Ma | *Radula sphaerocarpoides* Grolle[25] | Baltic amber: 35-55 Ma<br>Bitterfeld amber: ~23 Ma |
| | | *Radula oblongifolia* Casp. [26] | Baltic amber: 35-55 Ma<br>Bitterfeld amber: ~23 Ma |
| E | uniform: 99.6 - 385.3 Ma | *Frullania cretacea* Hentschel et al. [27] | Burmese amber: 99.6-112 Ma |
| F | uniform: 35.0 - 385.3 Ma | *Nipponolejeunea europaea* Grolle[28] | Baltic amber: 35-55 Ma<br>Bitterfeld amber: ~23Ma |
| G | uniform: 35.0 - 385.3 Ma | *Cheilolejeunea latiloba* (Casp.) Grolle[29] | Baltic amber: 35-55 Ma<br>Bitterfeld amber: ~23Ma |
| H | uniform: 15.0 - 385.3 Ma | *Marchesinia pusilla* Gradst.[30] | Dominican amber: 15-20 Ma |
| I | uniform: 35.0 - 385.3 Ma | *Spruceanthus polonicus* Grolle[31] | Baltic amber: 35-55 Ma |
| J | uniform: 15.0 - 385.3 Ma | *Lopholejeunea subnigricans* Gradst.[30] | Dominican amber: 15-20 Ma |
| K | uniform: 15.0 - 385.3 Ma | *Ceratolejeunea* spec.[30] | Dominican amber: 15-20 Ma |
| L | uniform: 15.0 - 385.3 Ma | *Lejeunea* spec.[32] | Dominican amber : 15-20 Ma |
| M | uniform: 15.0 - 385.3 Ma | *Cololejeunea* spec.[33] | Dominican amber: 15-20 Ma |
| N | uniform: 35.0 - 385.3 Ma | *Bazzania polyodus* (Casp.) Grolle[34] | Baltic amber: 35-55 Ma |
| O | uniform: 35.0 - 385.3 Ma | *Plagiochila groehnii* Grolle & Heinrichs[35] | Baltic amber: 35-55 Ma |
| P | uniform: 35.0 - | *Calypogeia stenzeliana* Grolle[23,36] | Baltic amber: 35-55 |

| | | | |
|---|---|---|---|
| | 385.3 Ma | | Ma<br>Bitterfeld amber: ~23Ma |
| Q | uniform: 35.0 - 385.3 Ma | *Cylindrocolea dimorpha* (Casp.) Grolle[23] | Baltic amber: 35-55 Ma<br>Bitterfeld amber: ~23Ma |
| R | uniform: 35.0 - 385.3 Ma | *Lophozia kutscheri* Grolle[23,37] | Baltic amber: 35-55 Ma<br>Bitterfeld amber: ~23Ma |
| S | uniform: 35.0 - 385.3 Ma | *Scapania hoffeinsiana* Grolle[38,39] | Baltic amber: 35-55 Ma<br>Bitterfeld amber: ~23Ma |

### 1.2.2. Discussion of fossil assignments:

#### 1.2.2.1. Sedimentary fossil:

*Metzgeriothallus sharonae*:

Fossil plant fragments from the Givetian (upper Middle Devonian) of New York consisting of furcated thalli with unistratose wings and a multistratose costa were described as *Metzgeriothallus sharonae* and assigned to the extant Metzgeriales[21]. Similar morphotypes occur not only in Metzgeriales but in several independent lineages of Jungermanniopsida, namely Metzgeriidae and Pelliidae[2]. Accordingly, we abstain from accepting *Metzgeriothallus* as a member of Metzgeriales but instead treat it as mrca of Jungermanniopsida (simple thallose and derived leafy liverworts).

#### 1.2.2.2. Amber fossils:

**A. Burmese amber**

Based on biostratigraphic work [40,41,42] Burmese amber is of mid Cretaceous origin (~ Cenomanian); therefore 99.6 Ma was employed as minimum age.

*Frullania cretacea*: The cosmopolitan genus *Frullania* is represented in numerous Cenozoic amber inclusions, and also present in mid Cretaceous Burmese amber (for review see[43]). It is characterized by reddish gametophytes with incubously-inserted, complicate bilobed leaves with the ventral lobe often forming a watersac. Several Burmese amber inclusions have been assigned to the extinct *F. cretacea*; a single gametophyte fragment from the same deposit has been described as *F. baerlocheri*. Since these fossils match the morphology of the *Frullania* crowngroup, they were treated as their mrca.

**B. Baltic and Bitterfeld amber**

The age of the Eocene Baltic amber is estimated as 35-55 Ma[44,45]. It is a very prominent source of fossil liverworts[23]. Numerous morphological details of these inclusions are preserved allowing for reliable assignments of the related species to extant genera. The Bitterfeld amber is of Oligocene origin and ca. 23 Ma old[46,47,48], and includes a liverwort diversity that is comparable to that of Baltic amber.

B.1. Porellales

*Ptilidium* spec.: A single fossil from Baltic amber was assigned to the extant species *Ptilidium pulcherimum*[23]. The deeply lobed leaves with long ciliate appendages are indicative of *Ptilidium*, but the extensive sequence similarities of the extant sister species *P. pulcherrium* and *P. ciliare*[22,49] as well as slight differences in leaf shape oppose this species assignment. Accordingly, the fossil was treated as *Ptilidium* spec. and assigned to the clade containing the genera *Ptilidium*, *Neotrichocolea* and *Trichocoleopsis*.

*Porella subgrandiloba*: This species is based on a fertile shoot fragment with incubously inserted, complicate bilobed, entire leaves with flat lobules and juvenile gynoecia, and resembles the extant species *Porella*

*grandiloba* and *P. obtusata*[23,24]. Due to the similaties to extant species in leaf and underleaf morphology, it was assigned to the *Porella* crown group.

*Radula oblongifolia* and *Radula sphaerocarpoides* are represented in Baltic and Bitterfeld amber; both species clearly belong to *Radula* based on the presense of *Radula*-type branching, incubous, complicate bilobed leaves and the absence of amphigastria. *R. sphaerocarpoides* is known only in sterile condition whereas some specimens of *R. oblongifolia* include fertile material with sporophytes[23]. The morphological overlap of both species with extant representatives of *Radula* allows for a crown group assignment. We abstain from an assignment to subclades of *Radula* because of the morphologically diffuse nature of these clades[50].

*Nipponolejeunea europaea*: Gametophytes of this genus occur frequently in Baltic and Bitterfeld amber. A Baltic specimen was described as the extinct *Nipponolejeunea europaea*[28] and assigned to the extant *N. subalpina*[23]. Heinrichs et al.[22] doubted the conspecificity of the fossil *N. europaea* and the extant *N. subalpina* because of extensive sequence similarities of the two extant *Nipponolejeunea* species (*N. pilifera*, *N. subalpina*). Following the suggestions of [51], the amber inclusions were thus treated as extinct species and assigned to a clade with *Nipponolejeunea* and its sister lineage *Jubula.*

*Cheilolejeunea latiloba*: Fertile amber inclusions of this species with intact gynoecia have been found in Baltic and Bitterfeld amber[23], supporting a treatment as mrca of the *Cheilolejeunea* crowngroup. The internal structure of *Cheilolejeunea* is not yet fully clarified and several genera are nested in it, e.g. *Aureolejeunea* and *Omphalanthus*[52]. This situation complicates a comparision of *Cheilolejeunea latiloba* with the extant diversity of Cheilolejeuneinae, and assignments to *Cheilolejeunea* subclades.

*Spruceanthus polonicus*: *Spruceanthus* is a small genus with three extant species in SE Asia and one in the Neotropics. A Baltic amber fossil with gynoecia matches the morphology of extant *Spruceanthus* and somewhat resembles the extant *S. semirepandus* from SE Asia[23]. The extinct species *Spruceanthus polonicus* was placed on the split of *Spruceanthus* and *Ptychanthus*.

B.2 Jungermanniales

*Bazzania polyodus*: This species was recognized in Baltic and Bitterfeld amber and identified as a member of the extant genus *Bazzania*[34] based on the incubous leaf insertion, bifid leaves, and irregularly quadrilaciniate underleaves. It is treated as mrca of *Bazzania* since its incomplete preservation hampers a comprehensive comparision to extant species.

*Plagiochila groehnii*: Two sterile shoot fragments from Baltic amber with alternating, toothed, wide spreading leaves are indicative of *Plagiochila* and resemble the extant *P. sciophila*[35]. Since taxonomically relevant fertile structures and branches are unknown, the species was not assigned to a certain crown group lineage but treated as its mrca.

*Calypogeia stenzeliana*: This fossil species is known only in sterile condition and is characterized by incubous leaves, bilobed amphigastria and sometimes tapering shoot apices. The vegetative structures resemble those of the Holarctic *Calypogeia suecica*[23]; lack of fertile structures and sporophytes only allow a treatment as the mrca of the *Calypogeia* crown group.

*Cylindrocolea dimorpha*: Several inclusions of ventrally branched, minute plants with succubously inserted, bilobed leaves in Baltic and Bitterfeld amber have been assigned to this morphologically variable, extinct species. The taxon was first assigned to *Cephaloziella*[34] but subsequently transferred to *Cylindrocolea* based on the lack of gemmae[23]. Since *Cylindrocolea* is probably nested in *Cephaloziella*, the fossil taxon was treated as mrca of a well supported clade containing both genera.

*Lophozia kutscheri*: This fossil species is characterized by 2-3-lobed, transversely inserted leaves with gemmiferous apices. It was assigned to a widely circumscribed genus *Lophozia* including *Barbilophozia*, and compared to the extant *Barbilophozia hatcheri*[23]. In recent years the genus *Barbilophozia* was separated from *Lophozia* based on molecular phylogenetic and morphological evidence[53]; hence, *L. kutscheri* was assigned as mrca of this genus.

*Scapania hoffeinsiana*: This fossil representative of the extant genus *Scapania* was based on a liverwort inclusion with sharply complicate-bilobed, tothed leaves, eplicate perianth and 4-valved capsule in Bitterfeld

amber[38] and subsequently recognized in Baltic amber[39]. The morphology of the fossil species indicates an affiliation to the extant subgenus *Scapania*[51,54]; hence *S. hoffeinsiana* was treated as the mrca of this subgenus.

**C. Dominican amber**

Dominican amber is of early to middle Miocene origin (15-20 Ma)[55]. It contains numerous inclusions of leafy liverworts, especially of the order Porellales[32]:

*Marchesinia pusilla*: Two species of *Marchesinia* were recognized by Gradstein[30]. One sterile inclusion was assigned to the extant *M. brachiata*, but the assignment needs verification according to[56]. The extinct species *Marchesinia pusilla* was assigned to the split of *Marchesinia* and *Frullanoides*.

*Lejeunea* spec. A sterile specimen in Dominican amber with *Lejeunea*-type branching, two cells wide ventral merophytes, bifid underleaves as well as complicate bilobed leaves with rounded lobes and strongly inflated lobules fully matched the morphology of extant *Lejeunea*[32]. The generic affiliation of a second taxon in Miocene Mexican amber (*Lejeunea palaeomexicana*) has recently been questioned[57].

*Lopholejeunea subnigricans*: This Dominican amber fossil is known only in sterile condition. However, based on the undivided underleaves and the characteristic blackish color of the cell walls it could be assigned to *Lopholejeunea* without much doubt. It is morphologically similar to *L. nigricans*[30].

*Ceratolejeunea* spec.: The fossil is sterile and can be classified to genus level only. It shares some characteristics with the extant *C. plumula*, e.g. the toothed leaves[30].

*Cololejeunea* spec.: This fossil is a syninclusion of the holotype of the lichen *Phyllopsora dominicanus*[33]; the complicate-bilobed, incubously inserted leaves and lack of underleaves are indicative of *Cololejeunea*

# Supplementary Information 2: Results

## 2.1. Chronogram for Jungermanniopsida (estimated with BEAST 1.6.2.)

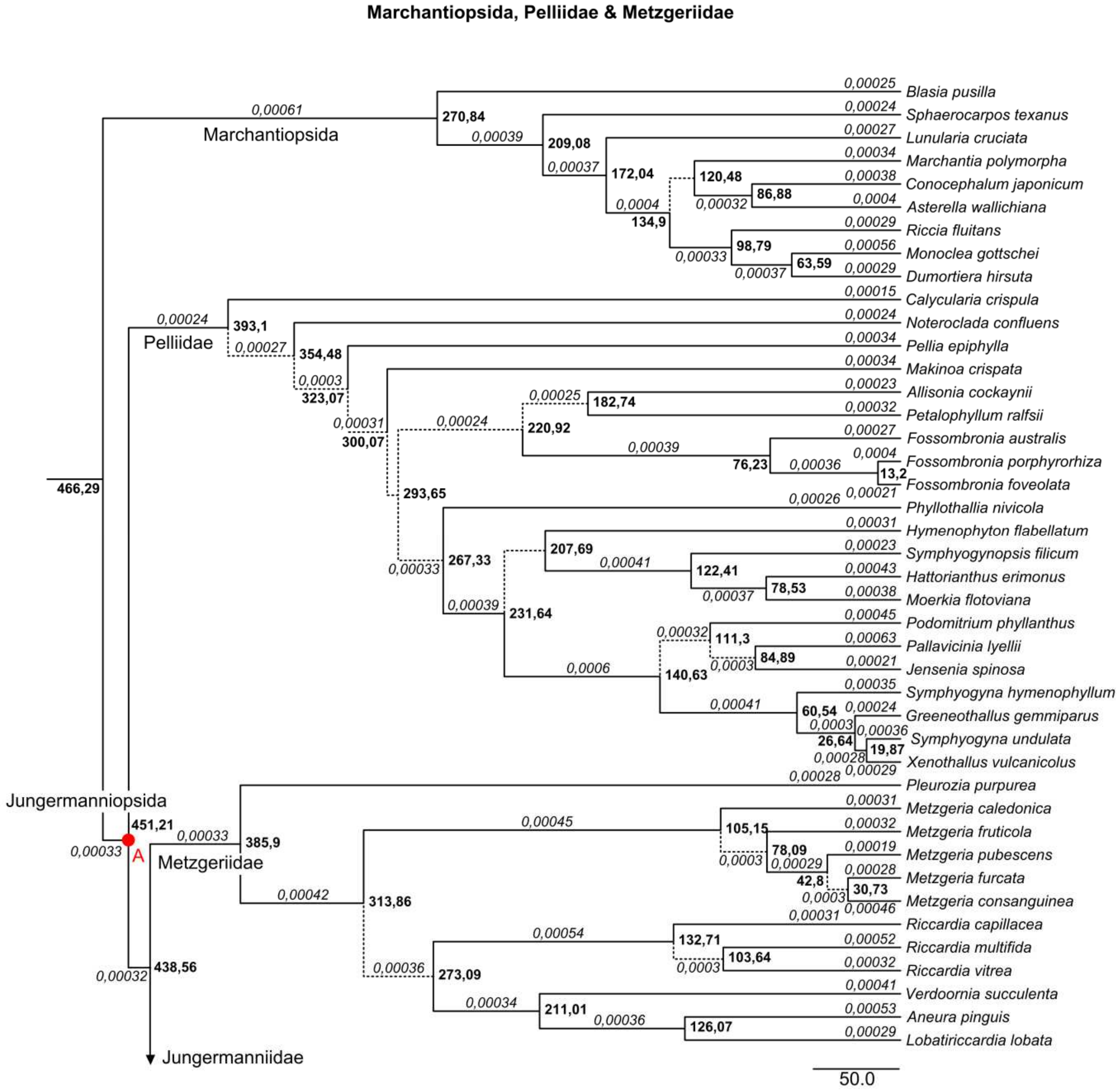

**Porellales I**

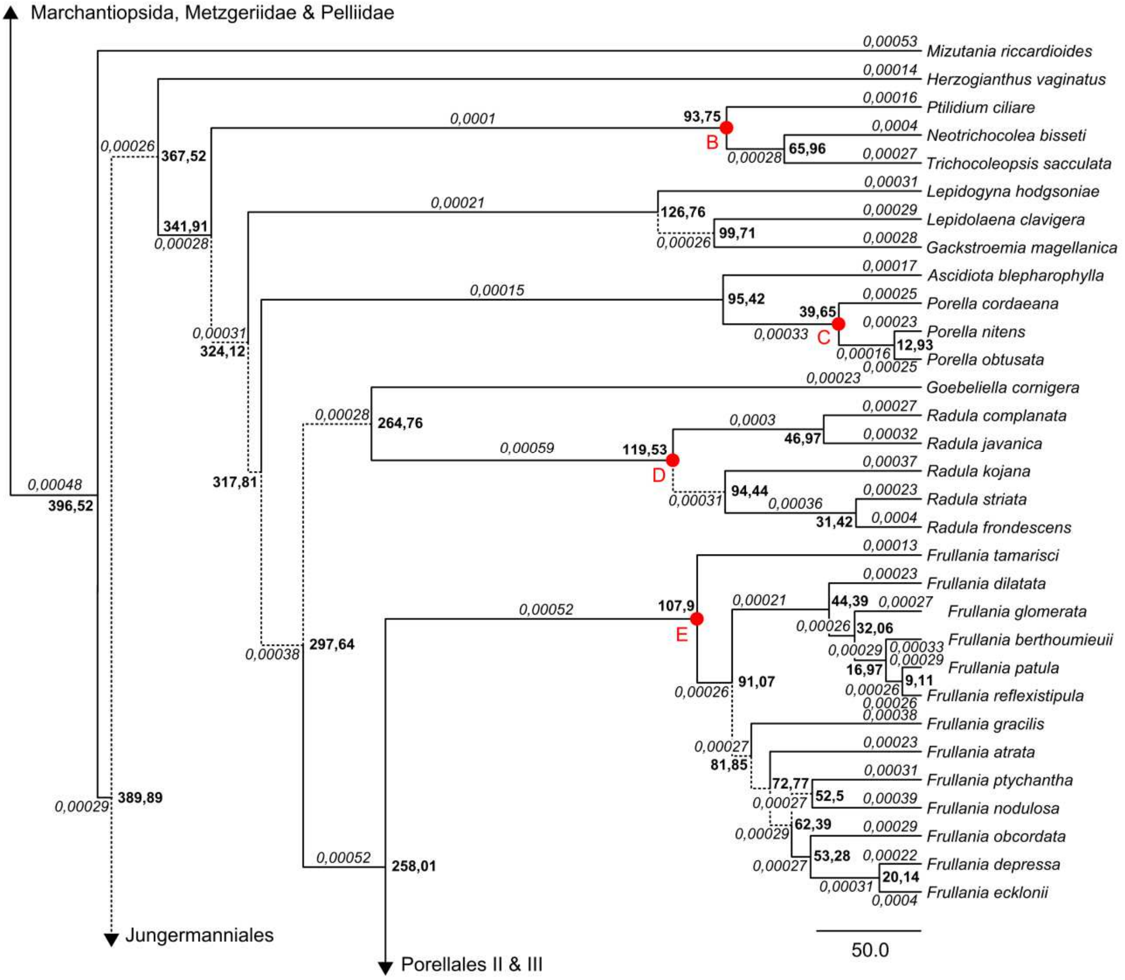

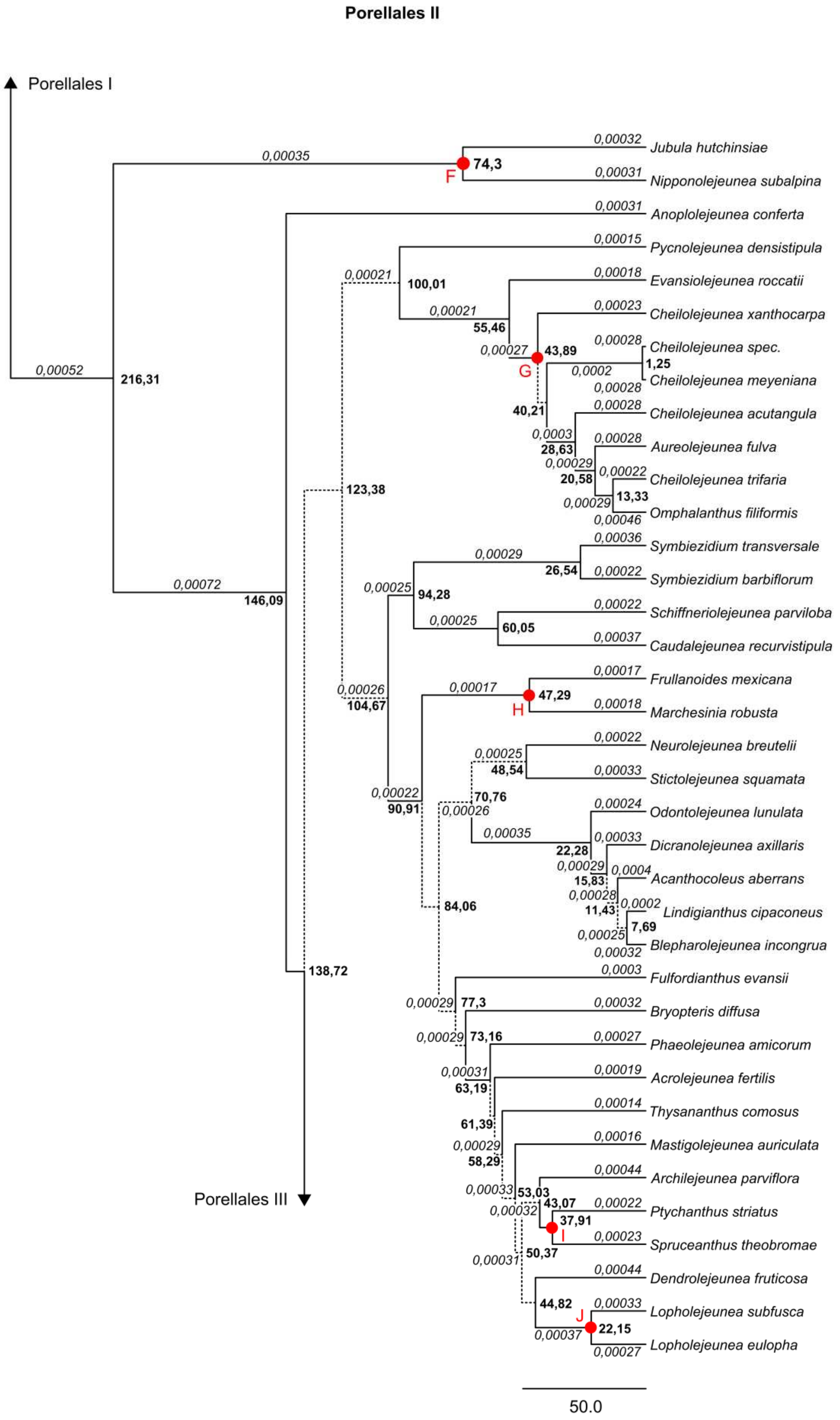

Porellales II
Porellales I
Porellales III
0,00052
216,31
0,00035
74,3
F
0,00032 Jubula hutchinsiae
0,00031 Nipponolejeunea subalpina
0,00031 Anoplolejeunea conferta
0,00015 Pycnolejeunea densistipula
0,00021
100,01
0,00018 Evansiolejeunea roccatii
0,00021
55,46
0,00023 Cheilolejeunea xanthocarpa
0,00027
43,89
G
0,00028 Cheilolejeunea spec.
1,25
0,0002
0,00028 Cheilolejeunea meyeniana
40,21
0,00028 Cheilolejeunea acutangula
0,0003
28,63
0,00028 Aureolejeunea fulva
0,00029
20,58
0,00022 Cheilolejeunea trifaria
13,33
0,00029
0,00046 Omphalanthus filiformis
123,38
0,00036 Symbiezidium transversale
0,00029
26,54
0,00022 Symbiezidium barbiflorum
0,00072
146,09
0,00025
94,28
0,00022 Schiffneriolejeunea parviloba
0,00025
60,05
0,00037 Caudalejeunea recurvistipula
0,00017 Frullanoides mexicana
0,00026
104,67
0,00017
47,29
H
0,00018 Marchesinia robusta
0,00022 Neurolejeunea breutelii
0,00025
48,54
0,00033 Stictolejeunea squamata
0,00022
90,91
70,76
0,00026
0,00024 Odontolejeunea lunulata
0,00035
22,28
0,00033 Dicranolejeunea axillaris
0,00029
15,83
0,0004 Acanthocoleus aberrans
0,00028
11,43
0,0002 Lindigianthus cipaconeus
84,06
7,69
0,00025
0,00032 Blepharolejeunea incongrua
138,72
0,0003 Fulfordianthus evansii
0,00029
77,3
0,00032 Bryopteris diffusa
0,00029
73,16
0,00027 Phaeolejeunea amicorum
0,00031
63,19
0,00019 Acrolejeunea fertilis
61,39
0,00014 Thysananthus comosus
0,00029
58,29
0,00016 Mastigolejeunea auriculata
0,00033
53,03
0,00044 Archilejeunea parviflora
0,00032
43,07
0,00022 Ptychanthus striatus
37,91
I
0,00023 Spruceanthus theobromae
0,00031
50,37
0,00044 Dendrolejeunea fruticosa
44,82
J
0,00033 Lopholejeunea subfusca
0,00037
22,15
0,00027 Lopholejeunea eulopha
50.0

**Porellales III**

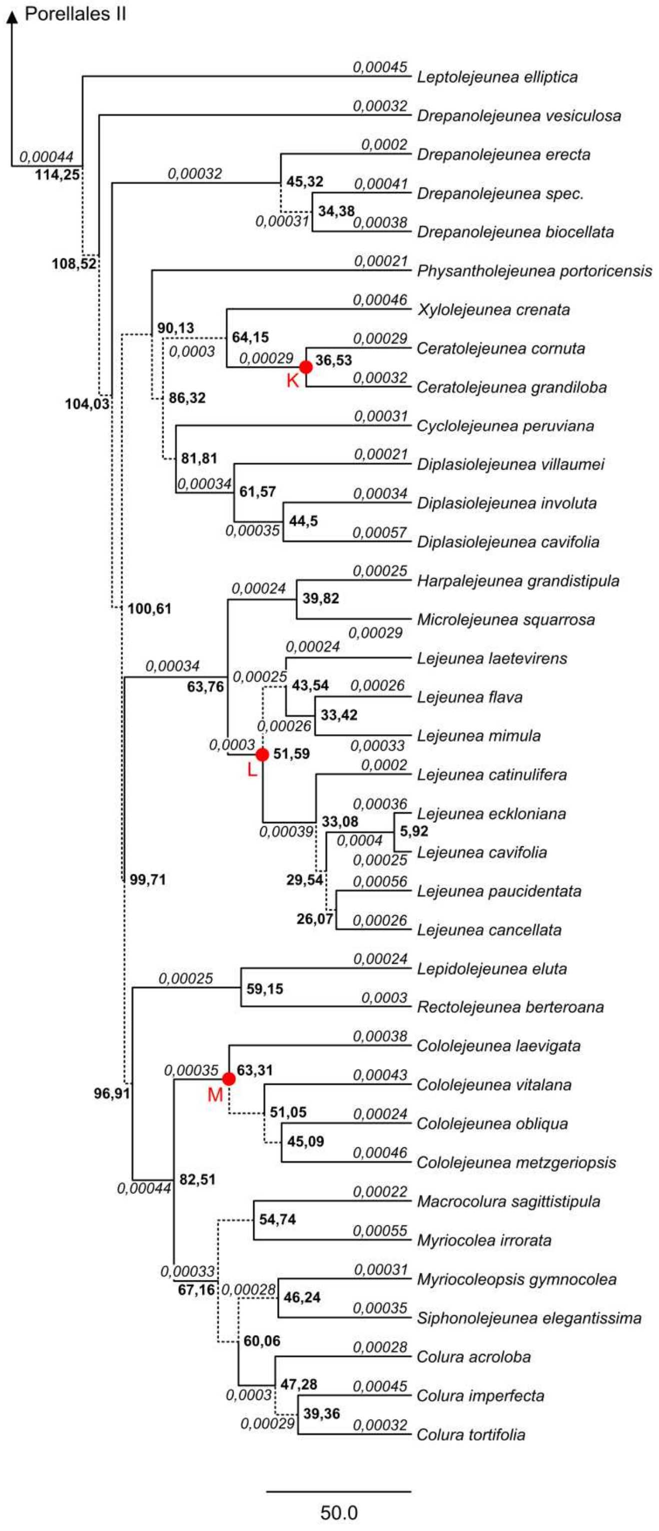

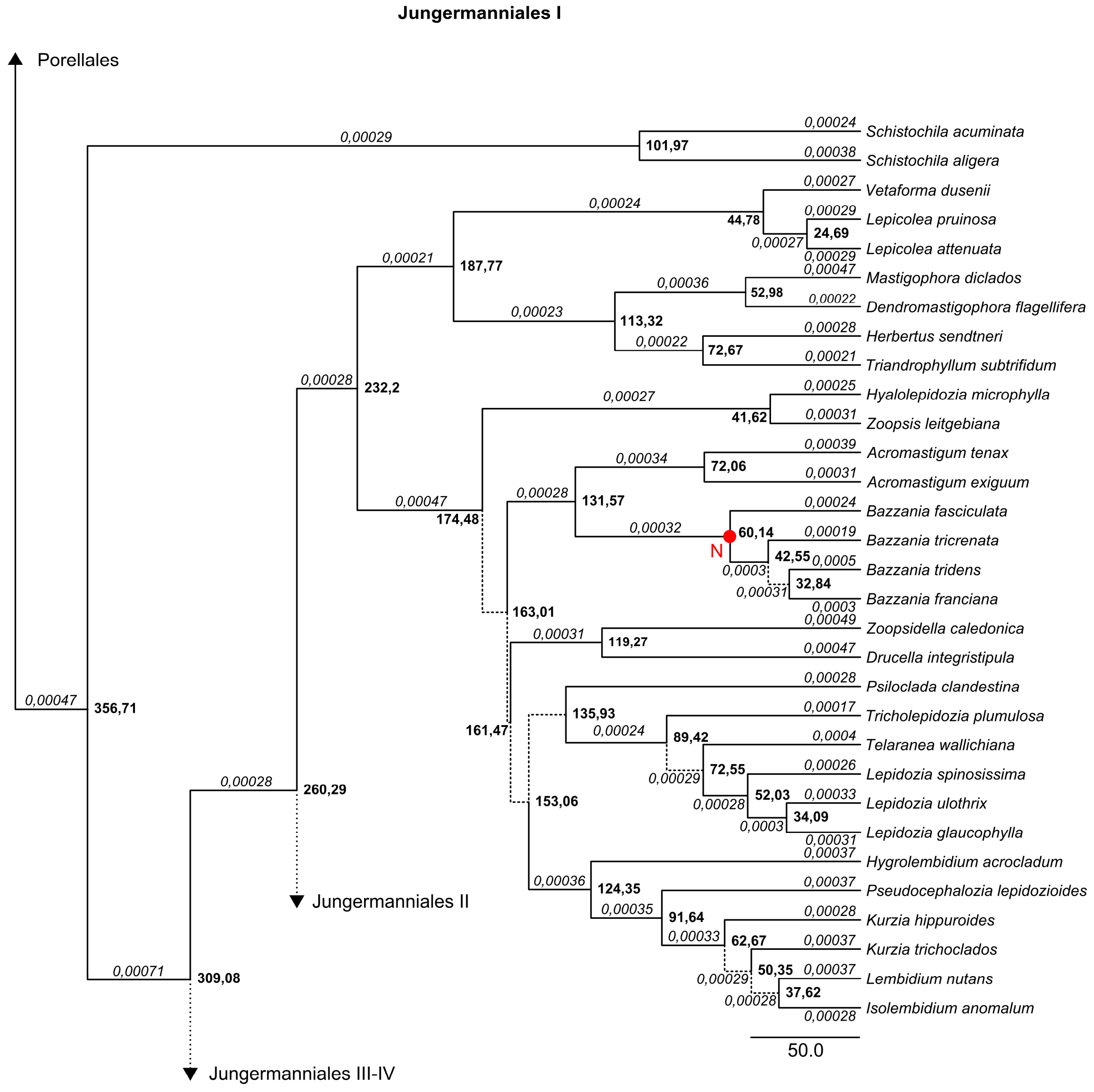
Jungermanniales I
Porellales
Schistochila acuminata
Schistochila aligera
Vetaforma dusenii
Lepicolea pruinosa
Lepicolea attenuata
Mastigophora diclados
Dendromastigophora flagellifera
Herbertus sendtneri
Triandrophyllum subtrifidum
Hyalolepidozia microphylla
Zoopsis leitgebiana
Acromastigum tenax
Acromastigum exiguum
Bazzania fasciculata
Bazzania tricrenata
Bazzania tridens
Bazzania franciana
Zoopsidella caledonica
Drucella integristipula
Psiloclada clandestina
Tricholepidozia plumulosa
Telaranea wallichiana
Lepidozia spinosissima
Lepidozia ulothrix
Lepidozia glaucophylla
Hygrolembidium acrocladum
Pseudocephalozia lepidozioides
Kurzia hippuroides
Kurzia trichoclados
Lembidium nutans
Isolembidium anomalum
Jungermanniales II
Jungermanniales III-IV
N
50.0

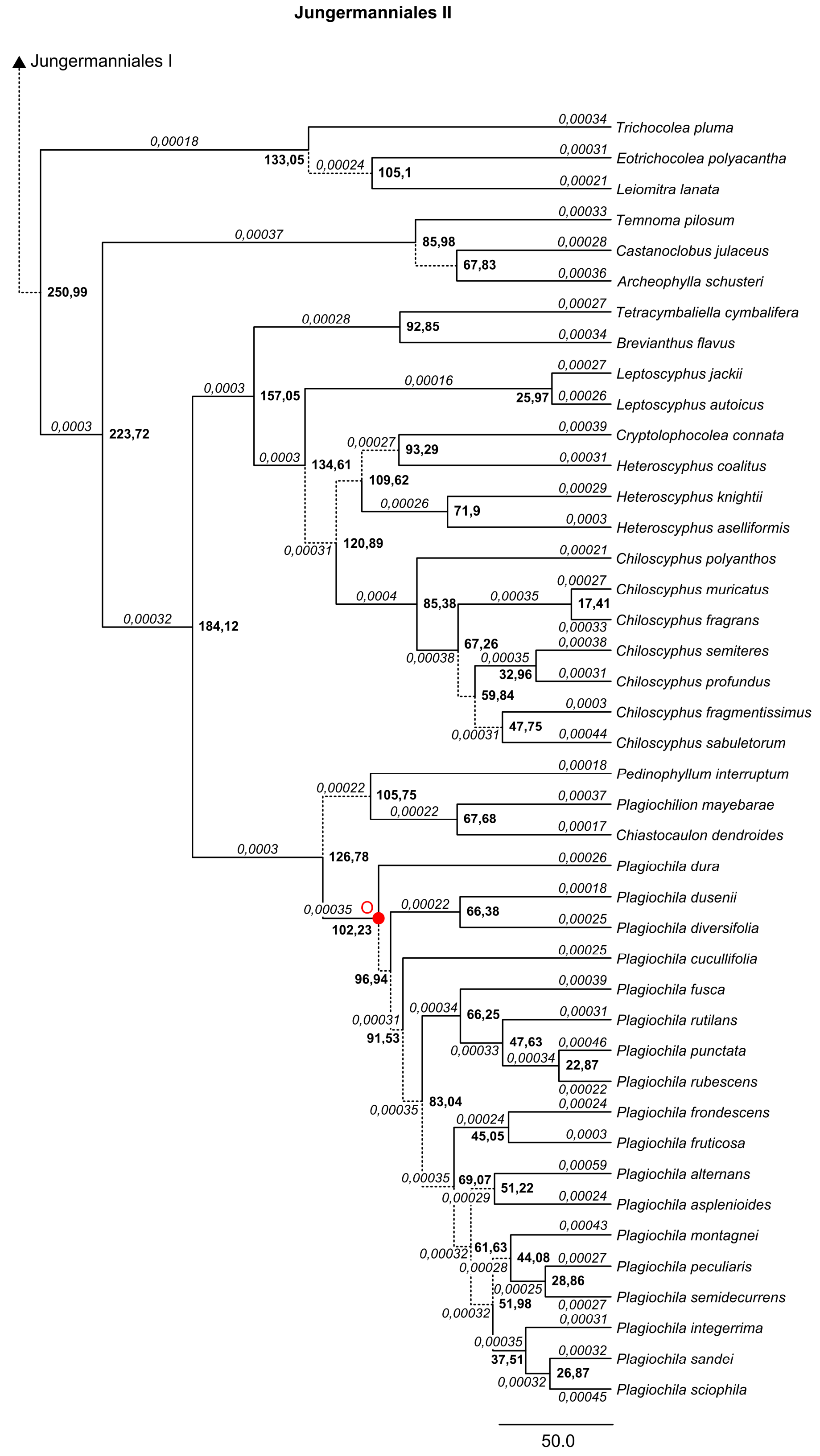
Jungermanniales II
Jungermanniales I
0,00018
133,05
0,00024
105,1
0,00034 Trichocolea pluma
0,00031 Eotrichocolea polyacantha
0,00021 Leiomitra lanata
0,00037
85,98
67,83
0,00033 Temnoma pilosum
0,00028 Castanoclobus julaceus
0,00036 Archeophylla schusteri
250,99
0,0003
223,72
0,00028
92,85
0,00027 Tetracymbaliella cymbalifera
0,00034 Brevianthus flavus
0,0003
157,05
0,00016
25,97
0,00027 Leptoscyphus jackii
0,00026 Leptoscyphus autoicus
0,0003
134,61
0,00027
93,29
0,00039 Cryptolophocolea connata
0,00031 Heteroscyphus coalitus
109,62
0,00026
71,9
0,00029 Heteroscyphus knightii
0,0003 Heteroscyphus aselliformis
0,00031
120,89
0,00021 Chiloscyphus polyanthos
0,0004
85,38
0,00035
17,41
0,00027 Chiloscyphus muricatus
0,00033 Chiloscyphus fragrans
0,00032
184,12
0,00038
67,26
0,00035
32,96
0,00038 Chiloscyphus semiteres
0,00031 Chiloscyphus profundus
59,84
0,0003 Chiloscyphus fragmentissimus
0,00031
47,75
0,00044 Chiloscyphus sabuletorum
0,00022
105,75
0,00018 Pedinophyllum interruptum
0,00022
67,68
0,00037 Plagiochilion mayebarae
0,00017 Chiastocaulon dendroides
0,0003
126,78
0,00026 Plagiochila dura
0,00035
102,23
0,00022
66,38
0,00018 Plagiochila dusenii
0,00025 Plagiochila diversifolia
96,94
0,00025 Plagiochila cucullifolia
0,00039 Plagiochila fusca
0,00031
91,53
0,00034
66,25
0,00031 Plagiochila rutilans
0,00033
47,63
0,00034
22,87
0,00046 Plagiochila punctata
0,00022 Plagiochila rubescens
0,00035
83,04
0,00024
45,05
0,00024 Plagiochila frondescens
0,0003 Plagiochila fruticosa
0,00035
69,07
0,00029
51,22
0,00059 Plagiochila alternans
0,00024 Plagiochila asplenioides
0,00032
61,63
0,00028
44,08
0,00043 Plagiochila montagnei
0,00027 Plagiochila peculiaris
0,00025
28,86
0,00027 Plagiochila semidecurrens
0,00032
51,98
0,00031 Plagiochila integerrima
0,00035
37,51
0,00032 Plagiochila sandei
0,00032
26,87
0,00045 Plagiochila sciophila
50.0

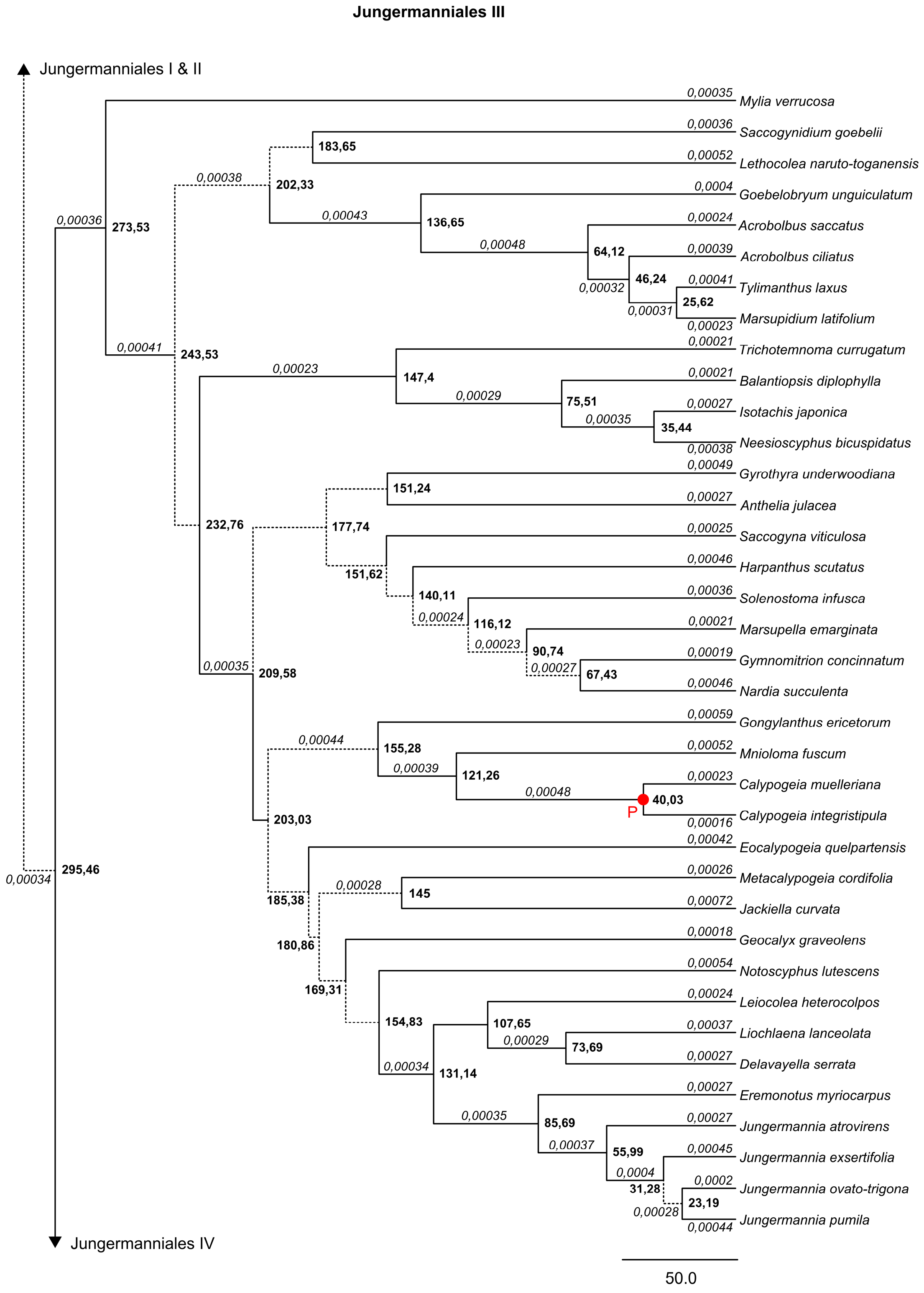
Jungermanniales III
Jungermanniales I & II
Jungermanniales IV
Mylia verrucosa
Saccogynidium goebelii
Lethocolea naruto-toganensis
Goebelobryum unguiculatum
Acrobolbus saccatus
Acrobolbus ciliatus
Tylimanthus laxus
Marsupidium latifolium
Trichotemnoma currugatum
Balantiopsis diplophylla
Isotachis japonica
Neesioscyphus bicuspidatus
Gyrothyra underwoodiana
Anthelia julacea
Saccogyna viticulosa
Harpanthus scutatus
Solenostoma infusca
Marsupella emarginata
Gymnomitrion concinnatum
Nardia succulenta
Gongylanthus ericetorum
Mnioloma fuscum
Calypogeia muelleriana
Calypogeia integristipula
Eocalypogeia quelpartensis
Metacalypogeia cordifolia
Jackiella curvata
Geocalyx graveolens
Notoscyphus lutescens
Leiocolea heterocolpos
Liochlaena lanceolata
Delavayella serrata
Eremonotus myriocarpus
Jungermannia atrovirens
Jungermannia exsertifolia
Jungermannia ovato-trigona
Jungermannia pumila
295,46
273,53
243,53
232,76
209,58
203,03
202,33
183,65
136,65
64,12
46,24
25,62
147,4
75,51
35,44
177,74
151,24
151,62
140,11
116,12
90,74
67,43
155,28
121,26
40,03
P
185,38
180,86
169,31
145
154,83
131,14
107,65
73,69
85,69
55,99
31,28
23,19
50.0

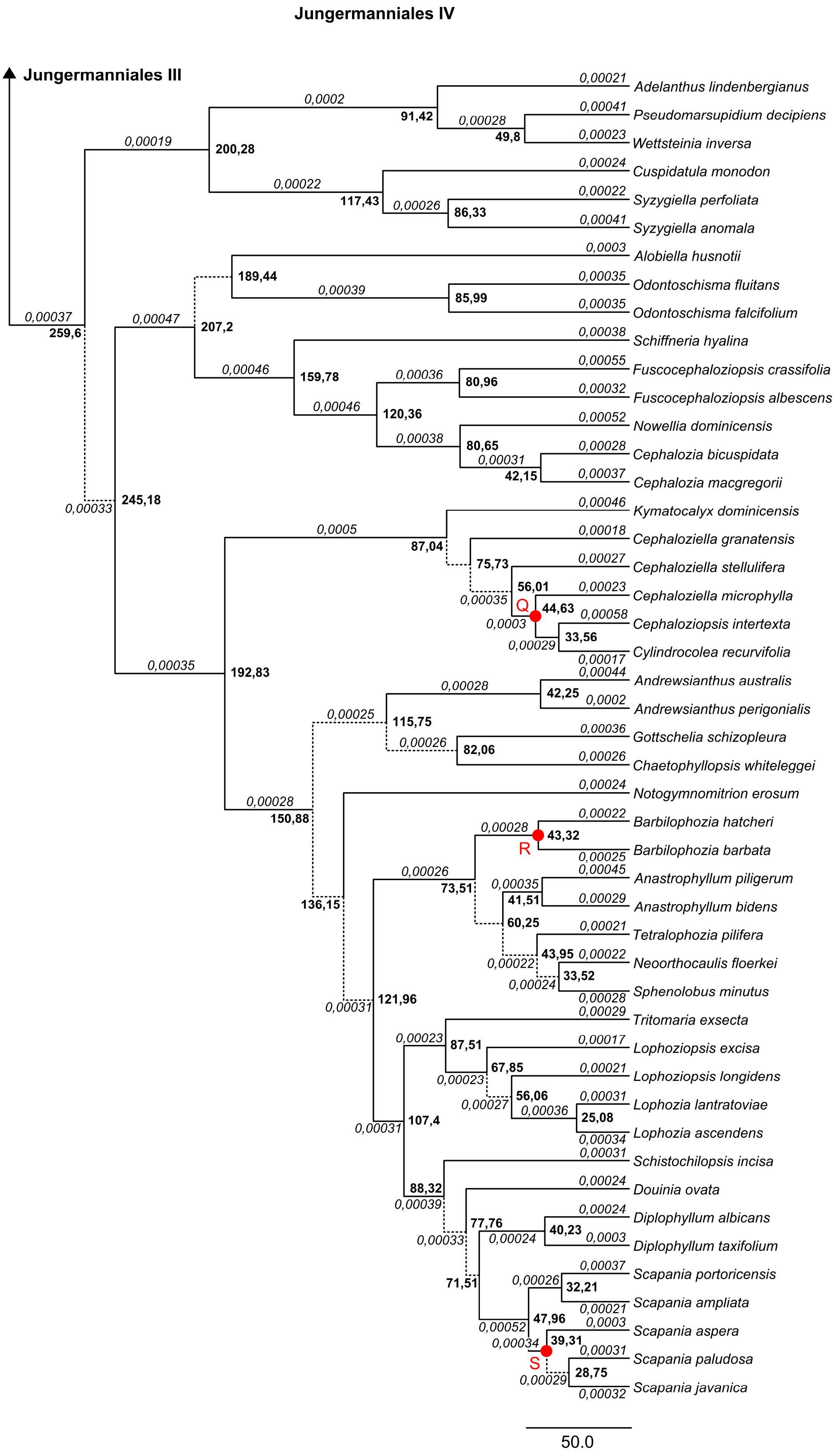


**Supplementary 2.1. Fig. 1:** Maximum credibility tree for Jungermanniopsida (estimated with Beast 1.6.2). Node ages (**bold**) and evolutionary rates (*italic*) are indicated. Branches with a posterior probability below 0.9 are indicated with dashed lines. Fossil calibrations are indicated as red dots with letters corresponding to Supplementary 1.2.1. Table 1. This is the chronogram as shown in Fig. 1.

**2.2. Penalized Likelihood (Chronoplots with APE)**

The mean substitution rates were estimated as relatively similar for all clades (Supplementary 2.2. Table 1), while the values for the minimum rates differ considerably, especially between Porellales and Jungermanniales, with the latter having much higher substitution rates. This is also apparent in the chronograms (Supplementary 2.2. Figs 2-5), with the colours corresponding to the substitution rates. There seem to be more rate changes within Porellales and some branches show very slow rates from 0.0-0.4 substitutions. Jungermanniales show mostly faster rates, with only few branches having a rate of 0.8-0.6. In summary, the results document the influence of the assigned fossil constrain and the absence of significant variation of substituation rates among the main clades of liverworts studied despite the lack of evidence for a constant molecular clock.

| | Min. | 1st Qu. | Median | Mean | 3rd Qu. | Max. |
|---|---|---|---|---|---|---|
| class Jungermanniopsida (simple thallose & leafy liverworts) | 0.5792 | 1.0000 | 1.0000 | 0.9976 | 1.0000 | 1.0000 |
| subclass Jungermanniidae (leafy liverworts) | 0.1166 | 1.0000 | 1.0000 | 0.9787 | 1.0000 | 1.0000 |
| order Porellales (mainly epiphytic liverworts) | 0.1703 | 1.0000 | 1.0000 | 0.9671 | 1.0000 | 1.0000 |
| order Jungermanniales (mainly generalistic liverworts) | 0.6757 | 1.0000 | 1.0000 | 0.9860 | 1.0000 | 1.0000 |

**Supplementary 2.2. Table 1:** Summary of the estimated rates. Rates are in units of substitutions per site per $10^9$ years shown again using the hierachical classification with class, subclass and orders (see 1.1.1). No major effect was detected for the subsampling and no evidence was found for a significant difference of the substituton rates estimated for the two orders.

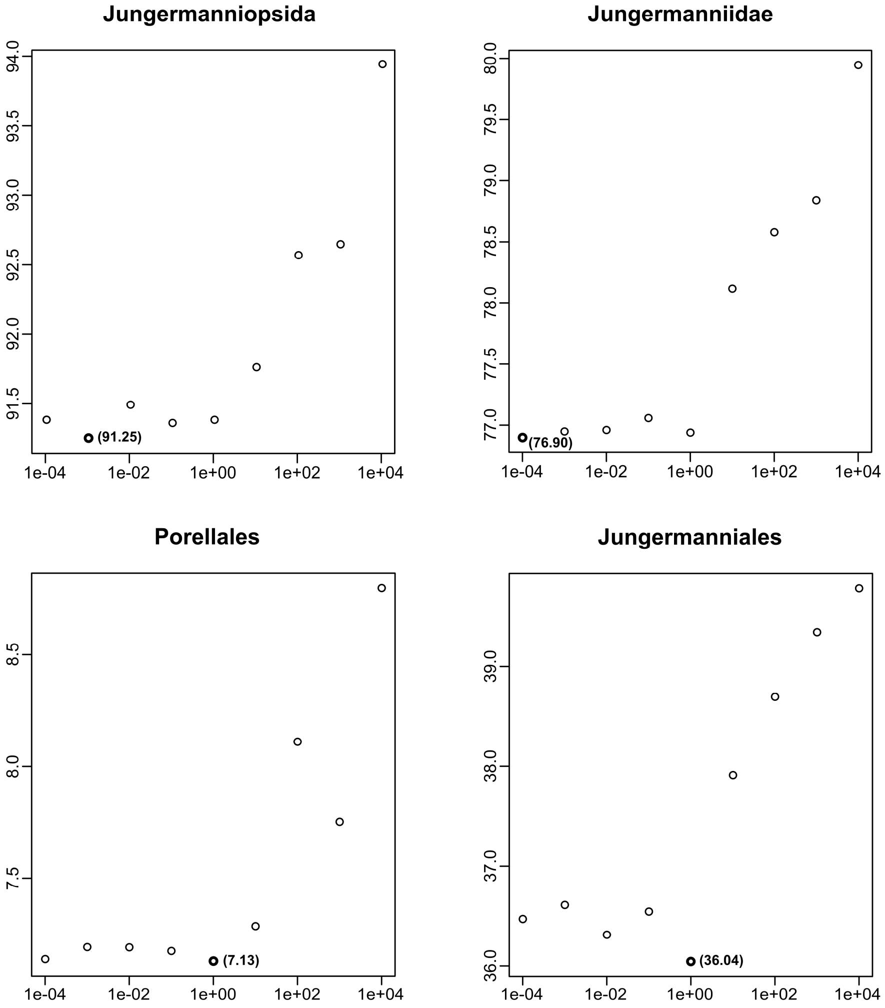


**Supplementary 2.2. Fig. 1:** Visualisation of the values of the cross-validation criterion (CV) plotted against the values of the smoothing parameter *lambda*. The lowest values indicate low differences between the predicted and observed values and the results of these estimates were therefore used for plotting the substitution rates. Again, this is done for the four hierachical taxonomic units explored (see 1.1.1).

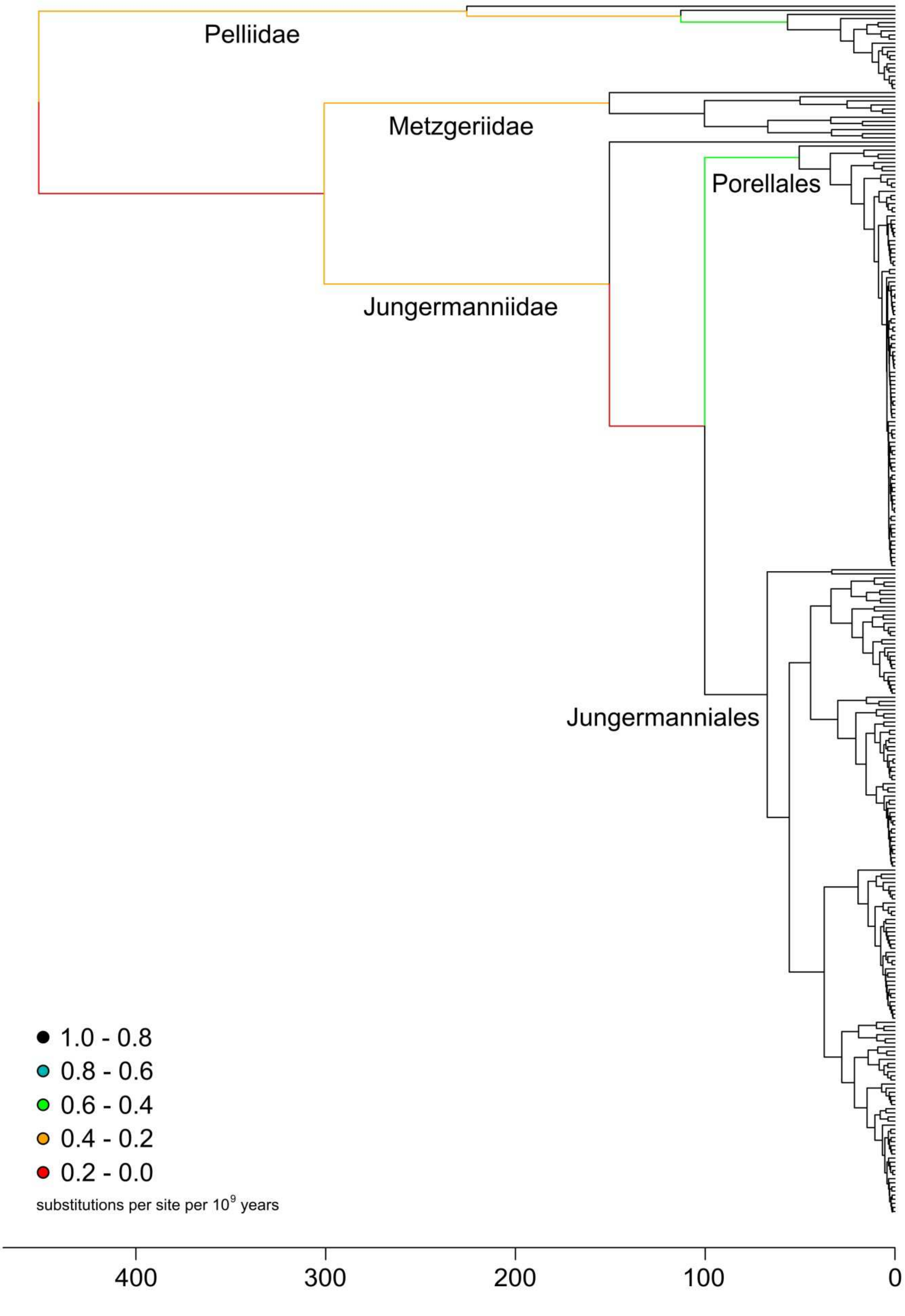


**Supplementary 2.2. Fig. 2:** Maximum credibility tree for Jungermanniopsida (leafy liverworts and simple thallose liverworts) estimated with Beast 1.6.2. Substitution rate estimates are plotted with the "chronoplot" function in APE (timescale in Ma).

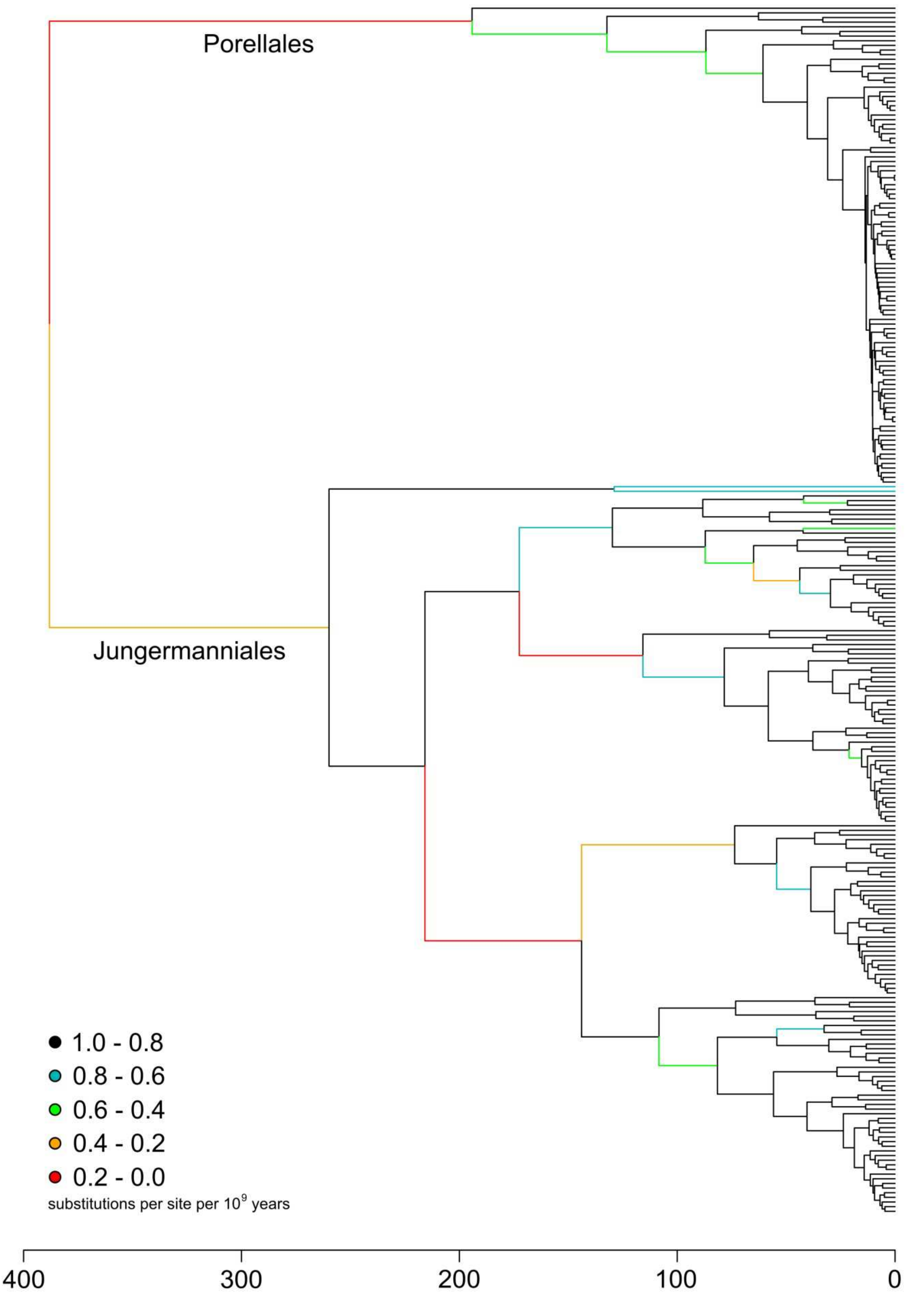


**Supplementary 2.2. Fig. 3:** The Jungermaniidae (leafy liverworts) clade from the maximum credibility tree for Jungermanniopsida estimated with Beast 1.6.2. Substitution rate estimates are plotted with the “chronoplot” function in APE (timescale in Ma).

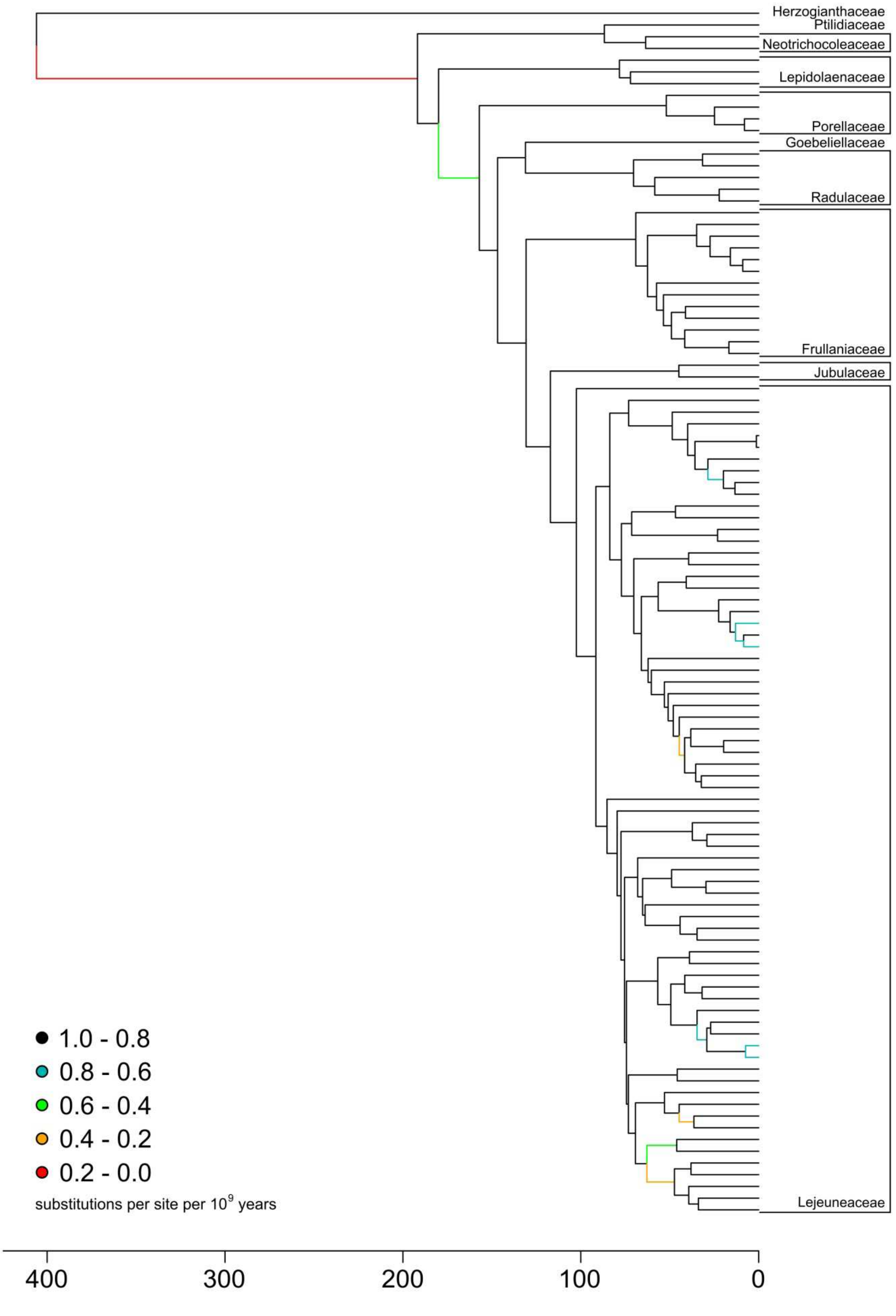


**Supplementary 2.2. Fig. 4:** The Porellales (mainly epiphytic leafy liverworts) clade from the maximum credibility tree for Jungermanniopsida estimated with Beast 1.6.2. Substitution rate estimates are plotted with the chronoplot function in APE (timescale in Ma).

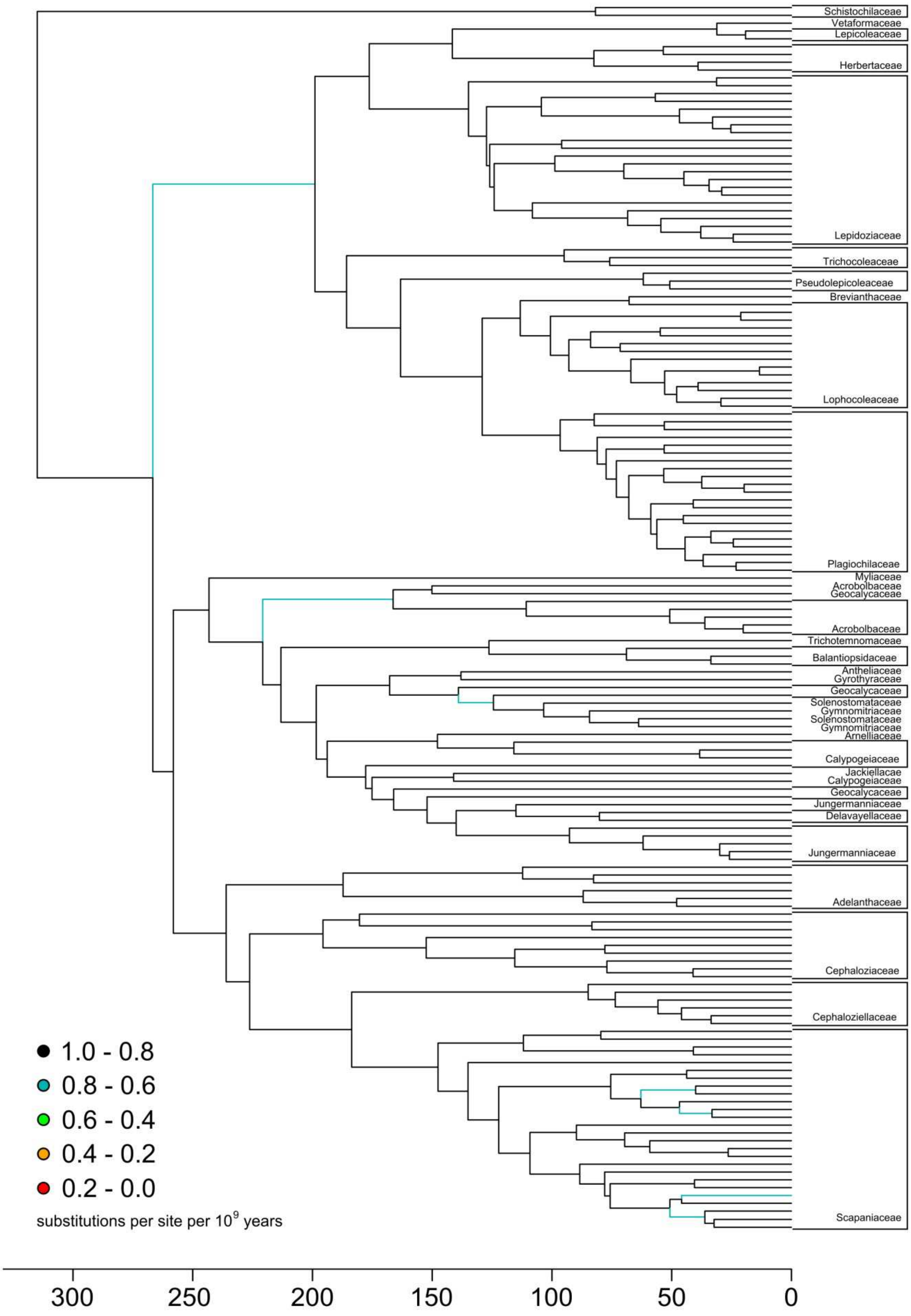


**Supplementary 2.2. Fig. 5:** The Jungermanniales (mainly generalistic leafy liverworts) clade from the maximum credibility tree for Jungermanniopsida estimated with Beast 1.6.2. Substitution rate estimates are plotted with the chronoplot function in APE (timescale in Ma).

**2.3. Lineages through time plots with TreeSIM**

The lineages through time plots (LTTPs) were estimated with 100 randomly chosen trees from the convergence phase of the Beast 1.6.2 analysis (see 1.1.1). The resulting confidence intervals are not very large and show the same trends as the LTTPs estimated for the four clades of the maximum credibility tree (Fig. 1B). To visualize times of putative rate changes the shift times estimated with LASER are included as arrows.

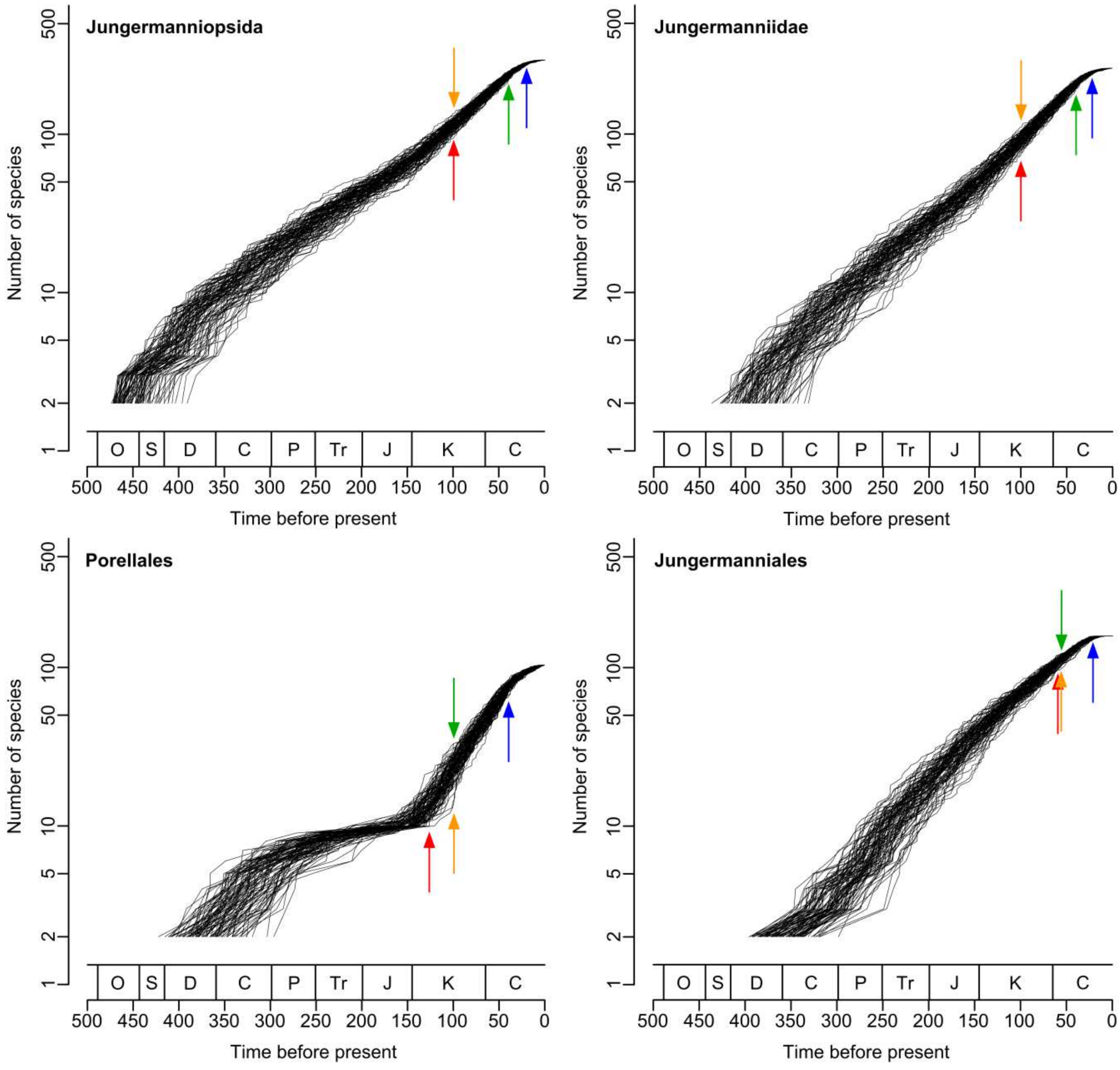


**Supplementary 2.3. Fig. 1:** Lineages through time plots estimated with TREESIM. The colored arrows indicate the shift times (points in time where the net diversification rate changes) estimated with the yule5rate model which was chosen with the AIC (Akaike Information Criterion) as the best fitting model in all analyses (see 2.6). The colors of the arrows correspond to the colors given to the values estimated for the yule5rate model in the modelfitting with LASER (see 2.6).

## 2.4. Gamma Statistics with APE and MCCR test with GEIGER

The significantly negative gamma values (γ; Supplementary 2.4. Table1) estimated for the four subsets (see 1.1.1) suggest that the cladogenic events are disproportionately distributed towards the root of the tree. This rejects the hypothesis that the diversification rates remained constant over time, indicates decreasing diversification rates and may also be an indicator of a rapid initial diversification[8]. All estimated γ values, except for Porellales, are well outside the null distribution (Supplementary 2.4. Fig. 1). The p-values indicate an overestimation of the γ due to incomplete taxon sampling. With a value of -2.1 the γ of Porellales falls just under the tail of the null distribution (Supplementary 2.4. Fig. 1).

| subsets | γ (CR) | p-values (MCCR) |
|---|---|---|
| class Jungermanniopsida | -4.564914 | 1.00 |
| subclass Jungermanniidae | -5.689822 | 1.00 |
| order Porellales | -2.123439 | 0.99 |
| order Jungermanniales | -5.981634 | -1.00 |

**Supplementary 2.4. Table 1:** Gamma statistics based on the maximum credibility tree (CR) and the p values obtained from MCCR analyses carried out to test the significance of the deviation.

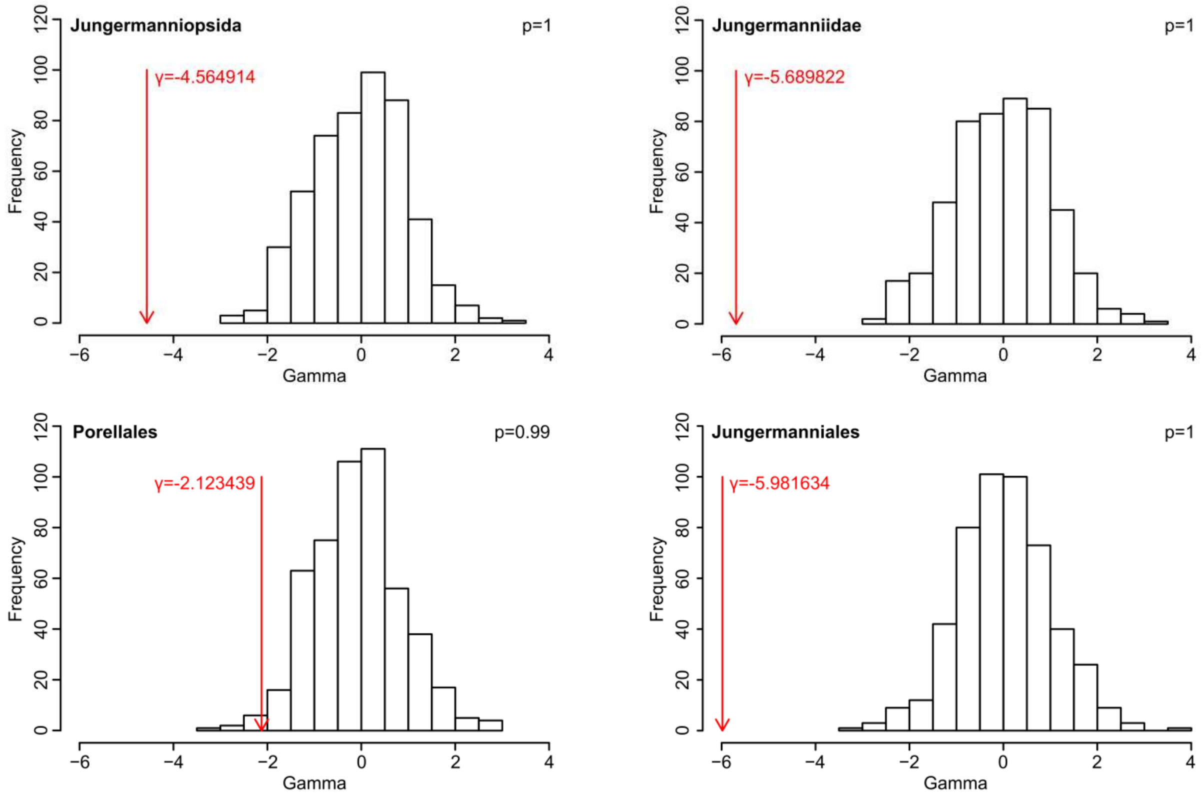


**Supplementary 2.4. Fig. 1:** Visualiyation of the null distributions of the gamma statistics with p-values derived from the MCCR test. The red arrows indicate the γ value as estimated with the CR test. All CR values were placed in the tails of the estimated distributions and were found significant.

## 2.5. Estimation of diversification rate shifts with TreePAR

These analyses were employed to explore rate-shifts accross 100 randonly sampled trees obtained from the convergence phase of the Beast analysis for four samplings: Jungermanniopsida, Jungermannidae, Porellales, and Jungerananiales (see 1.1.1).

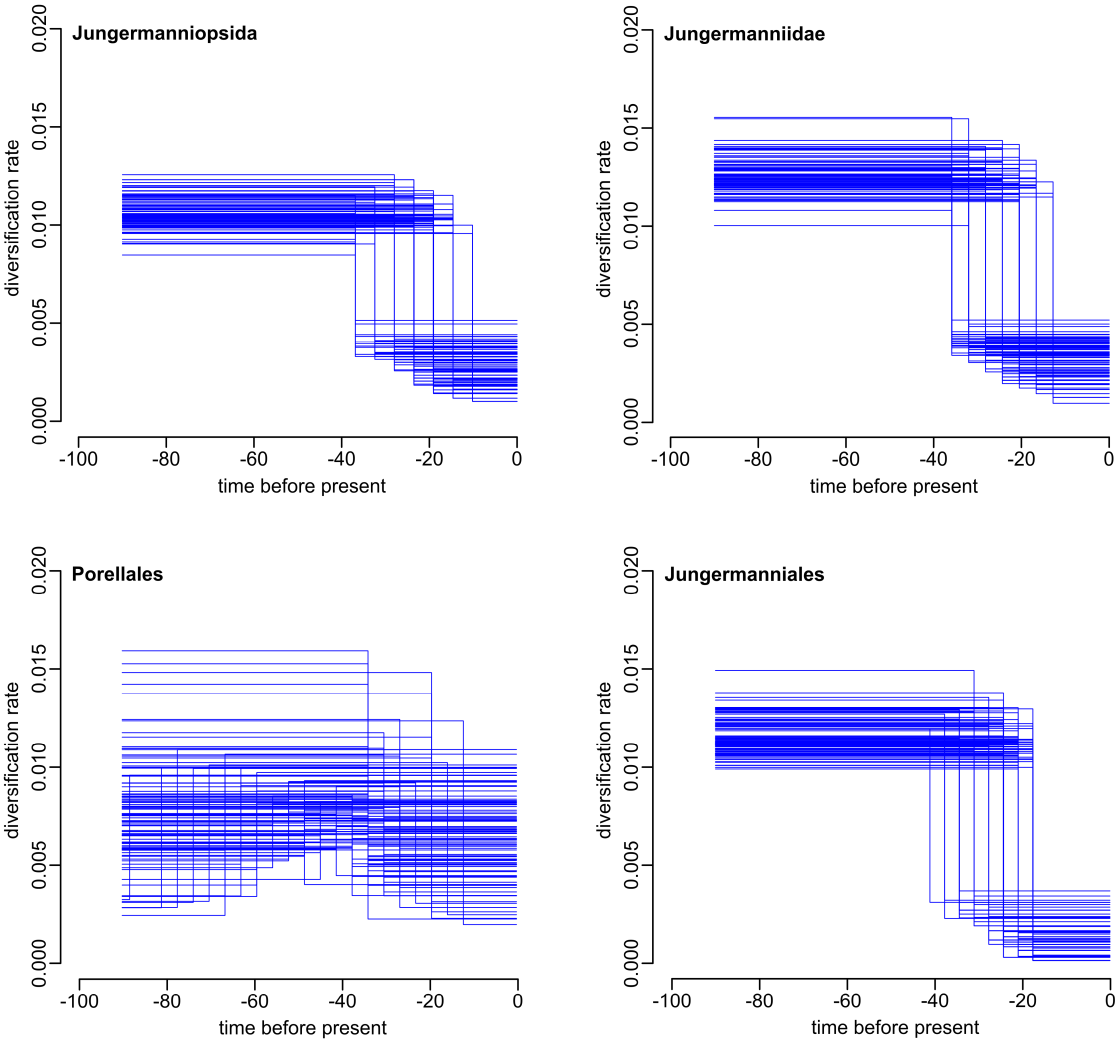


**Supplementary 2.5. Fig. 1:** Vizualization of the output of the TreePAR analyses with the time towards the presents plotted on the x-axis in million years towards the present, and the diversification rates plotted onto the y-axis. The blue lines show the distribution of rates for each tree considered. All analyses recovered a reduction of the diversification rate after 40 million years towards the present, whereas most trees of the Porellales showed also an increase of the rates around 80 to 60 million years towards the present.

**2.6. Modelfitting with LASER**

These tables present the results optained with the “fitdAICrc” and the “yule-n-rate” functions. The results for analyses with rate constant models are shown in Supplementary 2.6. Table 1 and the results for the rate variable models in Supplementary 2.6. Table 2. The rate constant model “pure birth” estimates only the speciation rate (λ) while the rate constant birth-death model additionaly estimates the extinction rate (μ). In this analysis the pure birth model was chosen with the Akaike Information Criterion (AIC) as the best fitting constant rate model (best). The DDX and DDL models fit density dependent speciation models to branch times and assume one rate shift. The “yule-n-rate” function can estimate up to 5 rate shifts as well as the shift time based on a pure birth (yule) model. Supplementary 2.6. Table 2 shows the results for the constant rate models The yule-5-rate model was chosen as best fitting model (best in Supplementary 2.6. Table 2) based on the AIC score. It is seen here as the best fitting of all models tested because it received a better AIC score than the pure birth model.

| | | **Jungermanniopsida** | **Jungermanniidae** | **Porellales** | **Jungermanniales** |
|---|---|---|---|---|---|
| **birth-death** | LH | -285.9849 | -254.8856 | -174.4428 | -244.4738 |
| | r1 | 0.009200704 | 0.01028827 | 0.01203396 | 0.009321269 |
| | a | 0 | 0 | 0 | 0 |
| | AIC | 575.9698 | 513.7711 | 352.8856 | 492.9477 |
| **pure birth (best)** | LH | -285.9849 | -254.8856 | -174.4428 | -244.4738 |
| | r1 | 0.009200704 | 0.01028827 | 0.01203396 | 0.009321269 |
| | AIC | 573.9698 | 511.7711 | 350.8856 | 490.9477 |

**Supplementary 2.6. Table 1:** Constant rate models. LH: likelihood, r1: net diversification rate, a: extinction fraction (extinction rate/speciation rate), AIC: Akaike Information Criterion.

| | | DDX | DDL | yule-2-rate | yule-3-rate | yule-4-rate | yule-5-rate (best) |
|---|---|---|---|---|---|---|---|
| **Jungermanniopsida** | **LH** | -282.1289 | -275.8456 | -257.241 | -253.675 | -250.8653 | -246.9981 |
| | **st1** | | | 22.15461 | 39.30597 | 126.7837 | 99.70935 |
| | **st2** | | | - | 19.86672 | 39.30597 | 99.70747 |
| | **st3** | | | - | - | 19.86672 | 39.30597 |
| | **st4** | | | - | - | - | 19.86672 |
| | **r1** | 0.02062544 | 0.01295814 | 0.01096744 | 0.01169846 | 0.009501562 | 0.01031821 |
| | **r2** | | | 0.002191284 | 0.007209806 | 0.01309443 | 4.767276 |
| | **r3** | | | - | 0.001913924 | 0.007209806 | 0.01304372 |
| | **r4** | | | - | - | 0.001913924 | 0.007209806 |
| | **r5** | | | - | - | - | 0.001913924 |
| | **xparam** | 0.1686242 | | | | | |
| | **kparam** | | 588.0043 | | | | |
| | **AIC** | 568.2578 | 555.6913 | 520.482 | 517.3501 | 515.7305 | 511.9961 |
| **Jungermanniidae** | **LH** | -249.0622 | -241.3152 | -222.7671 | -218.1685 | -216.3685 | -212.2882 |
| | **st1** | | | 7.68709287 | 39.30597 | 45.31878 | 99.70935 |
| | **st2** | | | | 22.15461 | 39.30597 | 99.70747 |
| | **st3** | | | | | 22.15461 | 39.30597 |
| | **st4** | | | | | | 22.15461 |
| | | | | | | | |
| | **r1** | 0.02818721 | 0.01475296 | 0.01108866 | 0.01383550 | 0.01327448 | 0.01245346 |
| | **r2** | | | 0.00100480 | 0.008075279 | 0.02033167 | 6.067443 |
| | **r3** | | | | 0.002125048 | 0.008075279 | 0.01483371 |
| | **r4** | | | | | 0.002125048 | 0.008075279 |
| | **r5** | | | | | | 0.002125048 |
| | **xparam** | 0.2147198 | | | | | |
| | **kparam** | | 520.008 | | | | |
| | **AIC** | 502.1244 | 486.6303 | 451.53413758 | 446.3370 | 446.7370 | 442.5764 |
| **Porellales** | **LH** | -174.3698 | -171.849 | -165.6515 | -156.6528 | -154.2654 | -150.5121 |
| | **st1** | | | 31.41612 | 126.7582 | 258.0115 | 126.7582 |
| | **st2** | | | | 39.3607 | 146.0875 | 99.70935 |
| | **st3** | | | | | 39.3607 | 99.70747 |
| | **st4** | | | | | | 39.3607 |
| | **r1** | 0.01384789 | 0.01682349 | 0.01555346 | 0.006075057 | 0.01262621 | 0.006075057 |
| | **r2** | | | 0.005681468 | 0.02155635 | 0.002071221 | 0.02438414 |
| | **r3** | | | | 0.006595756 | 0.02082457 | 24.26977 |
| | **r4** | | | | | 0.006595756 | 0.02075619 |
| | **r5** | | | | | | 0.006595756 |
| | **xparam** | 0.03783327 | | | | | |
| | **kparam** | | 206.0333 | | | | |
| | **AIC** | 352.7395 | 347.698 | 337.303 | 323.3055 | 322.5309 | 319.0241 |
| **Jungermanniales** | **LH** | -235.4998 | -232.1471 | -214.3916 | -209.1985 | -206.1352 | -203.6757 |
| | **st1** | | | 23.18535 | 66.24705 | 67.8467 | 59.83692 |
| | **st2** | | | | 22.87056 | 67.26448 | 56.06216 |
| | **st3** | | | | | 22.87056 | 55.98609 |
| | **st4** | | | | | | 22.87056 |
| | **r1** | 0.0417538 | 0.0137402 | 0.01177409 | 0.01356653 | 0.01304987 | 0.01317913 |
| | **r2** | | | 0.0005503569 | 0.009411897 | 0.06872758 | 0.002408339 |
| | **r3** | | | | 0.0002789234 | 0.00959347 | 0.2363308 |
| | **r4** | | | | | 0.0002789234 | 0.009717596 |
| | **r5** | | | | | | 0.0002789234 |
| | **xparam** | 0.3517625 | | | | | |
| | **kparam** | | 314.023 | | | | |
| | **AIC** | 474.9995 | 468.2943 | 434.7831 | 428.3969 | 426.2703 | 425.3514 |

**Supplementary 2.6. Table 2:** Rate variable models. Colored values refer to the LTTPs in Supplementary 2.3 Fig. 1. LH: likelihood, st: shift time, r1-5: net diversification rates, xparam: the x parameter in the density dependent exponential model, kparam: the K parameter in the logistic density dependent model, **AIC**: Akaike Information Criterion.

## 2.7. MEDUSA

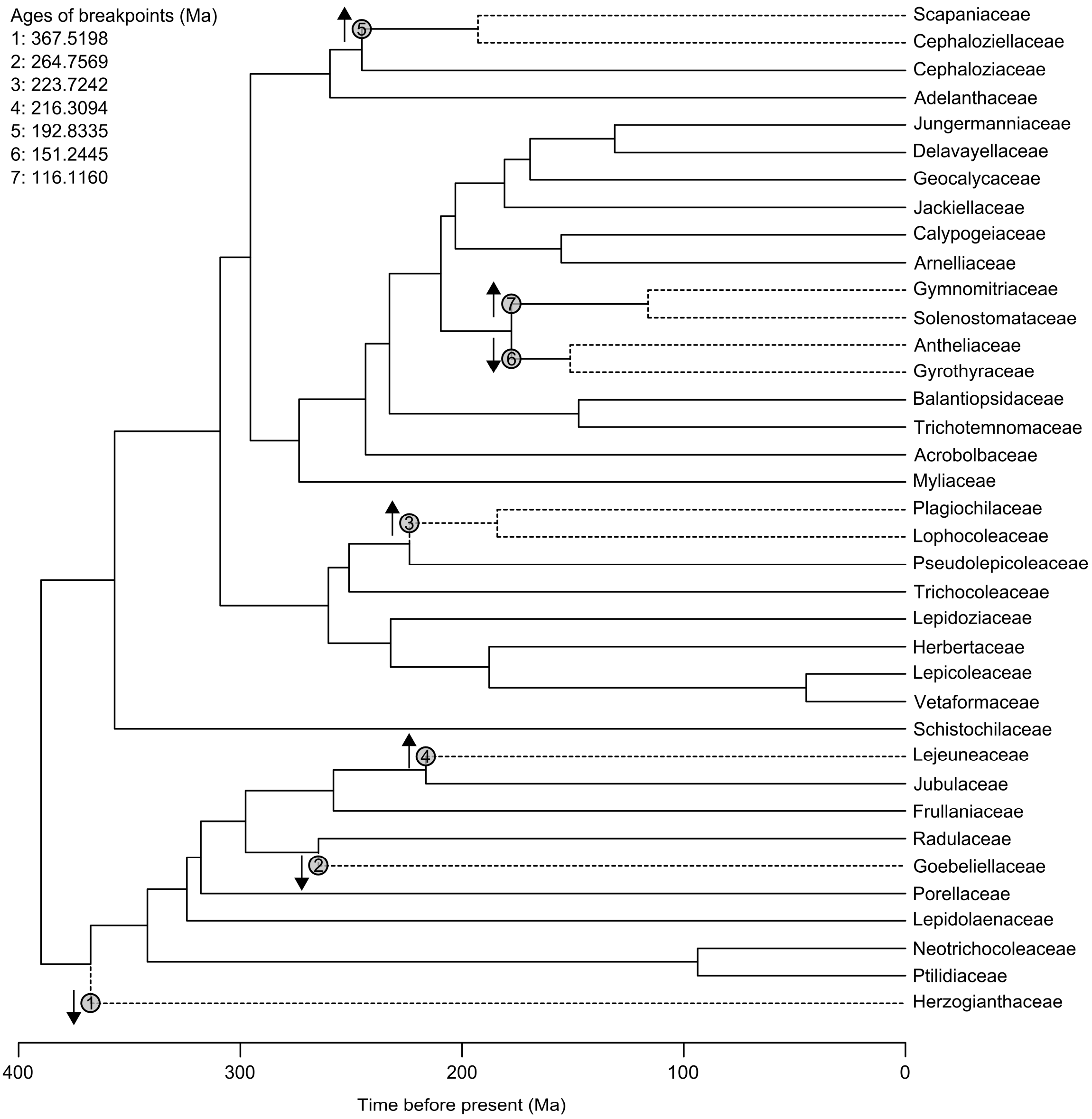


**Supplementary 2.7. Fig. 1:** Results of the MEDUSA analysis of the Jungermanniidae families. Breakpoints (diverisfication shift) estimated with the best model (yule) chosen with the corrected Akaike Information Criterion (AICc) and the times of the breaks. Arrows indicate an increased (up) or decreased (down) rate. The breakpoints are also shown in Fig. 1A using the same numbering scheme. Dashed lines indicate breakpoints placed at the crown of the clade, whereas continued lines indicate breakpoints at the stem of the clade.

## 2.8. BiSSE

Sumary of the BiSSE analyses with and without the addition of DIVERSTREE analyses carried out to test evidence suggesting dependence of diversification rate changes from ecological preferences. We added also a BiSSE independent test(Supplementary 2.8.1. Table 1).

### 2.8.1. Wilcoxon statistics to explore rate dependence of ecological preferences in sister clades

Clades with more than 50% epiphytes were considered as epiphytic, whereas clades with less than 50% epiphytes were classified as generalists. Test for group 1 and 2 compared the diversification rates of families classified as epiphytes versus families classified as generalists. Group 3 and 4 compared the difference in the diversification rates ofsister clades sharing the same ecological prefereence or show distinct ecological differences.

| Test | z-value | p-value |
|---|---|---|
| Group 1 & 2 Rates of epiphytes versus generalists | -0.94388 | 0.345 |
| Group 3 & 4 Rate changes in sister clades with eitehr the same ecological preferences or different ecological preferences | -1.98762 | 0.046 |

**Supplementary 2.8.1 Table 1:** Results of Wilcoxon statistics. $P < 0.5$ was considered to be significant. Thus, rate changes are associated with sister clades showing the transformation of ecological preferences, while epiphytic families did not show significant differences in their speciation rate compared to generalist families.

### 2.8.2. BiSSE with DIVERSITREE

For the BiSSE analysis with DIVERSITREE a character matrix with the substrate preference (E = epiphytic, G= generalistic) was assigned to the maximum credibility tree of Jungermanniopsida. Speciation rates ($\lambda$), extinction rates ($\mu$) and character state transitions between the states (q) were estimated with maximum likelihood and Bayesian inference. The analyses were performed with several parameter settings for which the likelihood and Akaike Information Criterion (AIC) values of the maximum likelihood estimation were best: 6 parameter ($\lambda_E \neq \lambda_G$, $\mu_E \neq \mu_G$, $q_{E>G} \neq q_{G>E}$ are estimated independently), 5 parameter ($\lambda_E, = \lambda_G$, $\mu_E \neq \mu_G$, $q_{E>G} \neq q_{G>E}$; $\lambda_E \neq \lambda_G$, $\mu_E = \mu_G$,$q_{E>G} \neq q_{G>E}$, $\lambda_E \neq \lambda_G$, $\mu_E \neq \mu_G$, $q_{E>G} = q_{G>E}$) and 4 parameter $\lambda_E, = \lambda_G$, $\mu_E, = \mu_G$, $q_{E>G}$, $q_{G>E}$; $\lambda_E, \neq \lambda_G$, $\mu_E, = \mu_G$, $q_{E>G} = q_{G>E}$; $\lambda_E, = \lambda_G$, $\mu_E, \neq \mu_G$, $q_{E>G} = q_{G>E}$). The p-values indicate that the differences between the variables of all models are significant.

| | ML | $\lambda_E$ | $\lambda_G$ | $\mu_E$ | $\mu_G$ | $q_{E>G}$ | $q_{G>E}$ | lnLik |
|---|---|---|---|---|---|---|---|---|
| 1 | $\lambda_E \neq \lambda_G$, $\mu_E \neq \mu_G$, $q_{E>G} \neq q_{G>E}$ | 0.08385 | 0.07077 | 0.04731 | 0.05809 | 0.00410 | 0.00031 | -1728.951 |
| 2 | $\lambda_E = \lambda_G$, $\mu_E \neq \mu_G$, $q_{E>G} \neq q_{G>E}$ | 0.07489 | | 0.03652 | 0.06256 | 0.00448 | 0.00026 | -1729.185 |
| 3 | $\lambda_E \neq \lambda_G$, $\mu_E = \mu_G$, $q_{E>G} \neq q_{G>E}$ | 0.09095 | 0.06871 | 0.05545 | | 0.00265 | 0.00039 | -1728.976 |
| 4 | $\lambda_E \neq \lambda_G$, $\mu_E \neq \mu_G$, $q_{E>G} = q_{G>E}$ | 0.11683 | 0.06444 | 0.08713 | 0.05015 | 0.00070 | | -1732.712 |
| 5 | $\lambda_E = \lambda_G$, $\mu_E = \mu_G$, $q_{E>G} \neq q_{G>E}$ | 0.10918 | | 0.09505 | | 0.00149 | 0.00074 | -1750.202 |
| 6 | $\lambda_E \neq \lambda_G$, $\mu_E = \mu_G$, $q_{E>G} = q_{G>E}$ | 0.09183 | 0.07174 | 0.05817 | | 0.00050 | | -1733.682 |
| 7 | $\lambda_E = \lambda_G$, $\mu_E \neq \mu_G$, $q_{E>G} = q_{G>E}$ | 0.07901 | | 0.04377 | 0.06591 | 0.00040 | | -1735.377 |

**Supplementary 2.8.2. Table 1:** Results of the maximum likelihood analyses for 7 parameter settings.

| | Parameter | mcmc | quant. | $\lambda_E$ | $\lambda_G$ | $\mu_E$ | $\mu_G$ | $q_{E>G}$ | $q_{G>E}$ | p |
|---|---|---|---|---|---|---|---|---|---|---|
| 1 | $\lambda_E \neq \lambda_G$, $\mu_E \neq \mu_G$, $q_{E>G} \neq q_{G>E}$ | 1 | 95% | 0.03493 | 0.02984 | 0.04453 | 0.03275 | 0.00585 | 0.00034 | - |
| | | 2 | 2.5% | 0.04755 | 0.04214 | 0.00123 | 0.026484 | 0.00151 | 0.00014 | 0.001723 |
| | | | 97.5% | 0.08906 | 0.07725 | 0.05318 | 0.06494 | 0.00838 | 0.00056 | 0.001717 |
| 2 | $\lambda_E = \lambda_G$ ,$\mu_E \neq \mu_G$, $q_{E>G} \neq q_{G>E}$ | 1 | 95% | 0.02479 | | 0.03351 | 0.02703 | 0.00593 | 0.00024 | - |
| | | 2 | 2.5% | 0.04893 | | 0.00180 | 0.03402 | 0.00158 | 0.00015 | 0.001723 |
| | | | 97.5% | 0.078 | | 0.04027 | 0.06573 | 0.00854 | 0.00044 | 0.001718 |
| 3 | $\lambda_E \neq \lambda_G$, $\mu_E = \mu_G$, $q_{E>G} \neq q_{G>E}$ | 1 | 95% | 0.02561 | 0.02737 | 0.03003 | | 0.00476 | 0.00034 | - |
| | | 2 | 2.5% | 0.06548 | 0.04129 | 0.02547 | | 0.001297 | 0.000227 | 0.001726 |
| | | | 97.5% | 0.09617 | 0.07397 | 0.06116 | | 0.006829 | 0.000626 | 0.001720 |
| 4 | $\lambda_E \neq \lambda_G$, $\mu_E \neq \mu_G$, $q_{E>G} = q_{G>E}$ | 1 | 95% | 0.04902 | 0.02871 | 0.05884 | 0.03184 | 0.00058 | | - |
| | | 2 | 2.5% | 0.05467 | 0.03855 | 0.01247 | 0.02115 | 0.00029 | | 0.001733 |
| | | | 97.5% | 0.11252 | 0.07356 | 0.08238 | 0.06001 | 0.00098 | | 0.001727 |
| 5 | $\lambda_E = \lambda_G$, $\mu_E = \mu_G$, $q_{E>G} \neq q_{G>E}$ | 1 | 95% | 0.03492 | | 0.03749 | | 0.00187 | 0.00067 | - |
| | | 2 | 2.5% | 0.07511 | | 0.05834 | | 0.00076 | 0.00043 | 0.001750 |
| | | | 97.5% | 0.11912 | | 0.10551 | | 0.00299 | 0.0012 | 0.001745 |
| 6 | $\lambda_E \neq \lambda_G$, $\mu_E = \mu_G$, $q_{E>G} = q_{G>E}$ | 1 | 95% | 0.02639 | 0.02831 | 0.03096 | | 0.00036 | | - |
| | | 2 | 2.5% | 0.06528 | 0.04346 | 0.02702 | | 0.00033 | | 0.001734 |
| | | | 97.5% | 0.09598 | 0.07606 | 0.06273 | | 0.00076 | | 0.001729 |
| 7 | $\lambda_E = \lambda_G$, $\mu_E \neq \mu_G$, $q_{E>G} = q_{G>E}$ | 1 | 95% | 0.02763 | | 0.03585 | 0.03003 | 0.00029 | | - |
| | | 2 | 2.5% | 0.05072 | | 0.00694 | 0.03495 | 0.00024 | | 0.001734 |
| | | | 97.5% | 0.08257 | | 0.04870 | 0.06963 | 0.00059 | | 0.001728 |

**Supplementary 2.8.2. Table 2:** Results of the BiSSE analysis with diversitree. MCMC 1 results are from the 95% quantile of the first MCMC analysis that is done to optimize the model values. MCMC 2 results are from the second MCMC analysis run with the optimized model. The 2.5% values are from the outer margins of the posterior distribution, the 97.5% values represent the inner part oft the distribution.

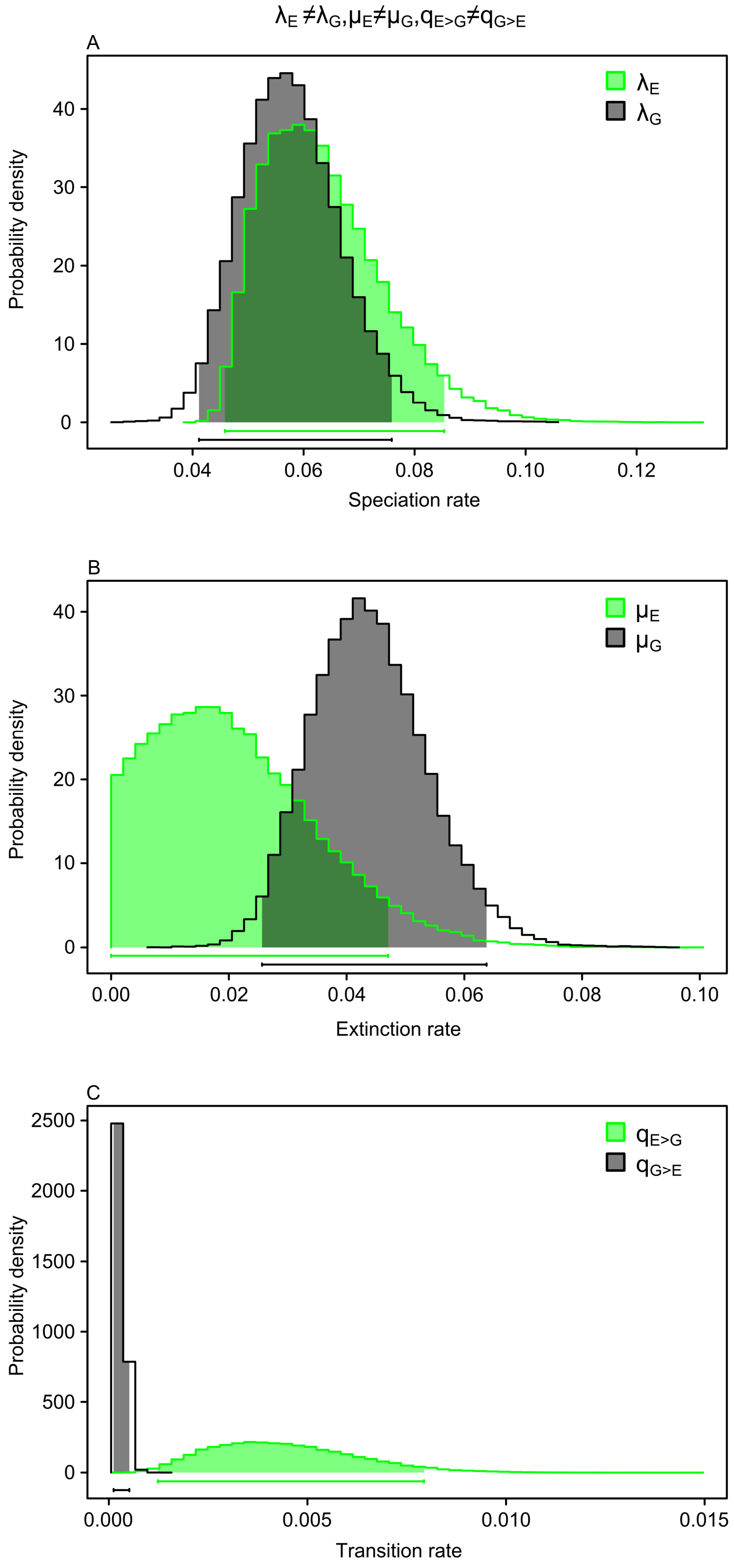


**Supplementary 2.8.2. Fig. 1:** Posterior probability distribution from the analysis with 6 variable parameters (λ: speciation rates, μ: extinction rates, q: transition rates).

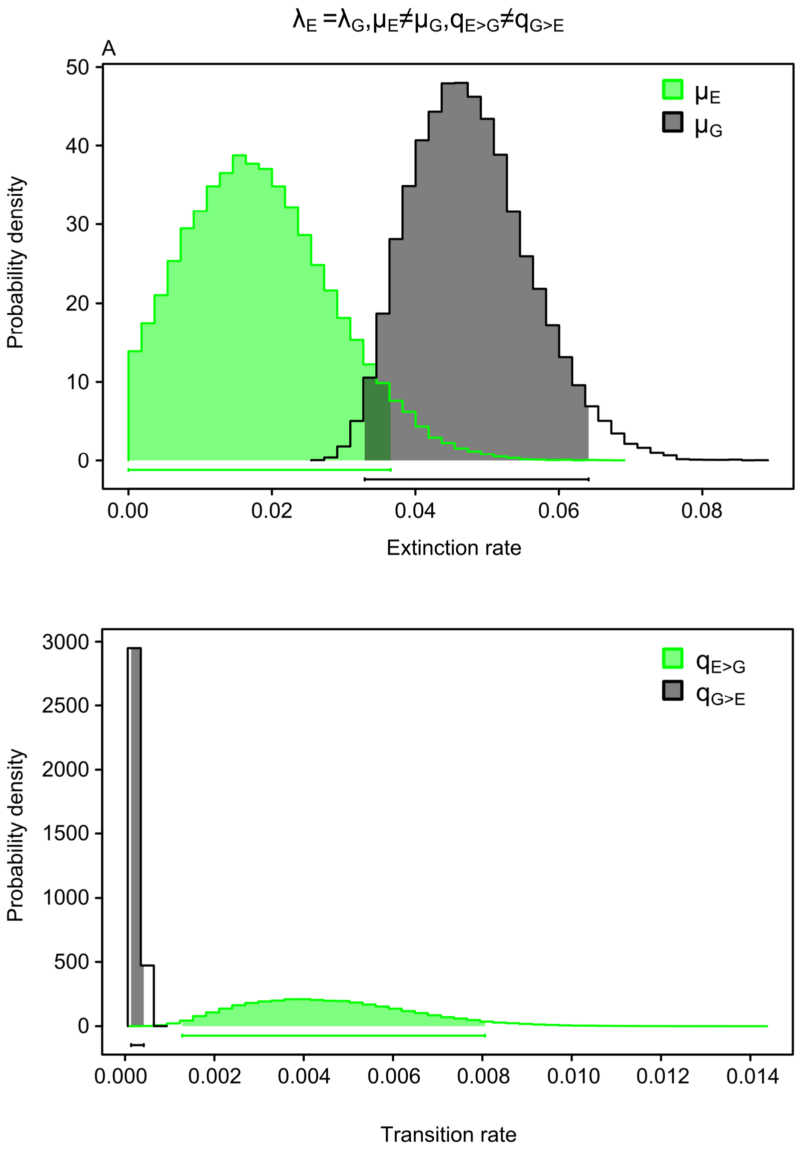


**Supplementary 2.8.2. Fig. 1:** Posterior probability distribution from the analysis with constant speciation rates λ (μ: extinction rates, q: transition rates).

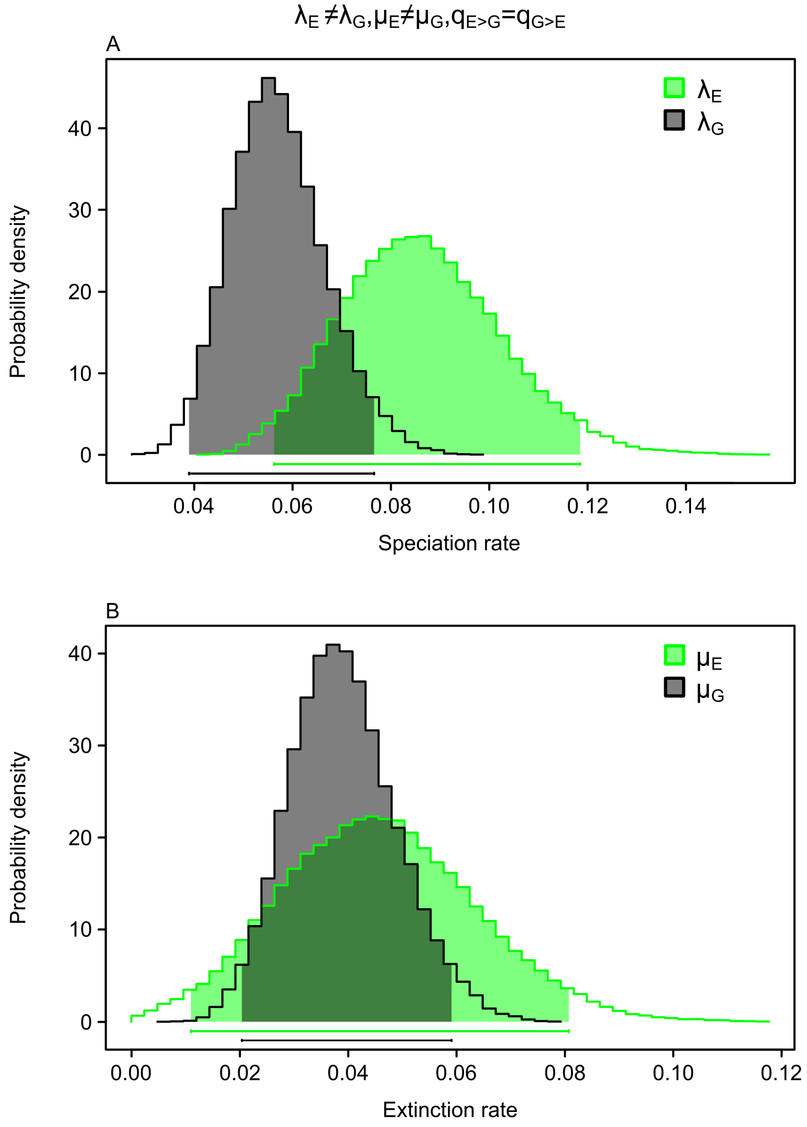


**Supplementary 2.8.2. Fig. 1:** Posterior probability distribution from the analysis with constant transition rates q (λ: speciation rates, μ: extinction rates).

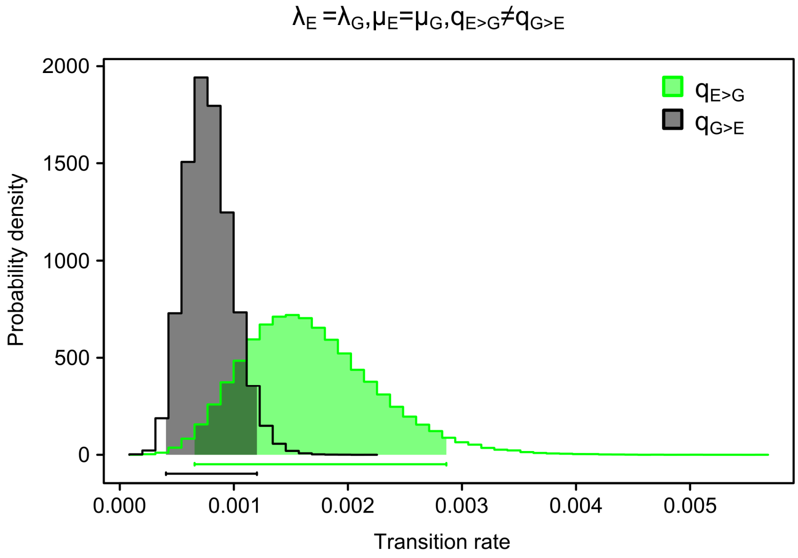


**Supplementary 2.8.2. Fig. 1:** Posterior probability distribution from the analysis with constant speciation rates λ and constant extinction rates μ (q: transition rates).

## 2.9. Estimation of speciation and extinction rates with DIVERSITREE

| | find.mle | | mle estimation | |
|---|---|---|---|---|
| | λ | μ | λ | μ |
| Jungermanniopsida | 0.009200707 | 0.000000000 | 8.932355e-03 | 7.699746e-07 |
| Jungermanniidae | 0.01028827 | 0.000000000 | 0.01028827 | 0.00000000 |
| Porellales | 0.009321272 | 0.000000000 | 0.01203397 | 0.00000000 |
| Jungermanniales | 0.009321272 | 0.000000000 | 0.009321272 | 0.00000000 |

**Supplementary 2.9. Table 1:** Estimated speciation (λ) and extinction rates (μ).

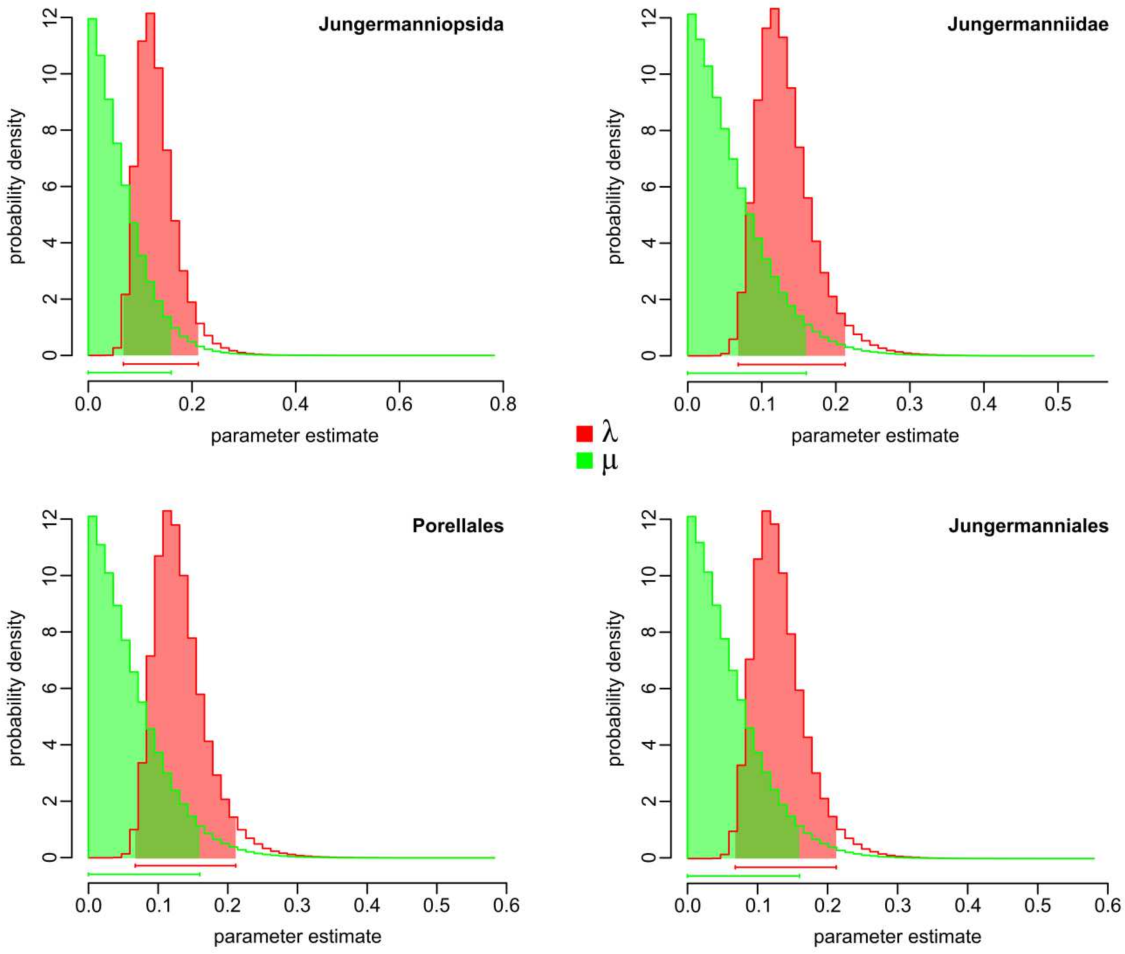


**Supplementary 2.9 Fig. 1:** Results of the mcmc-cross-validation of the birth-death model: posterior probability distributions of the speciation (λ) and extinction (μ) rates for the four subclades (see 1.1.1).

## Supplementary Information 3: References


1. R Development Core Team. R: *A Language and Environment for Statistical Computing.* (R Foundation for Statistical Computing, Vienna, 2008).
2. Crandall-Stotler, B., Stotler, R. E. & Long, D. G. Phylogeny and classification of the Marchantiophyta. *Edinb. J. Bot.* **66**, 158-198 (2009).
3. Sanderson, M. J. Estimating absolute rates of molecular evolution and divergence times: a penalized likelihood approach. *Mol. Biol. Evol.* **19**, 101-109 (2002).
4. Paradis, E., Claude, J. & Strimmer, K. APE: Analyses of phylogenetics and evolution in R language. *Bioinformatics* **20**, 289-290 (2004).
5. Paradis, E. *Analysis of Phylogenetics and Evolution with R* (Springer, New York, 2012).
6. Stadler, T. Simulating trees with a fixed number of extant species. *Syst. Biol.* **60**, 676-684 (2011).
7. Pybus, O. G. & Harvey, P. H. Testing macro-evolutionary models using incomplete molecular phylogenies. *Phil. Trans. R. Soc. B* **267**, 2267-2272 (2000).
8. Harmon, L. J., Weir, J. T., Brock, C. D., Glor, R. E. & Challenger, W. GEIGER: investigating evolutionary radiations. *Bioinformatics* **24**, 129-131 (2008).
9. Stadler, T. Mammalian phylogeny reveals recent diversification rate shifts. *Proc. Natl. Acad. Sci. USA* **108**, 6187-6192 (2011).
10. Rabosky, D. L. LASER: A maximum likelihood toolkit for detecting temporal shifts in diversification rates from molecular phylogenies. *Evol. Bioinf. Online* **2**, 247-250 (2006).
11. Maddison, W. P. & Maddison, D. R. Mesquite: a modular system for evolutionary analysis. Version 2.75 (2011).
12. FitzJohn, R.G. Diversitree: comparative phylogenetic analyses of diversification in R. *Methods Ecol. Evol.* **3**, 1084-1092 (2012).
13. FitzJohn, R. G. Quantitative traits and diversification. *Syst. Biol.* **59**, 619-633 (2010).
14. FitzJohn, R.G., Maddison W. P. & Otto, S. P. Estimating trait-dependent speciation and extinction rates from incompletely resolved phylogenies. *Syst. Biol.* **58**,595-611 (2009).
15. Benton, M. J. & Donoghue, P. C. J. Paleontological evidence to date the tree of life. *Mol. Biol. Evol.* **24**, 26-53 (2007).
16. Benton, M. J., Donoghue, P. C. J. & Asher, R. J. [Calibrating and constraining molecular clocks] *The Timetree of Life* [Hedges, S. B. & Kumar, S. (eds)] [35-86] (Oxford University Press, New York, 2009).
17. Clarke, J. T., Warnock, R. C. M. & Donoghue, P. C. J. Establishing a time-scale for plant evolution. *New Phytol.* **192**, 266-301 (2011).
18. Donoghue, P. C. J. & Benton, M. J. Rocks and clocks: calibrating the tree of life using fossils and molecules. *Trends Ecol. Evol.* **22**, 424-431 (2007).
19. Kenrick, P., Wellman, C. H., Schneider, H. & Edgecombe, G. D. A timeline for terrestrialization: consequences for the carbon cycle in the Palaeozoic. *Phil. Trans. R. Soc. B* **367**, 519-536 (2012).
20. Wellmann, C. H., Osterloff, P. L. & Mohiuddin, U. Fragments of the earliest land plants. *Nature* **425**, 282-284 (2003).
21. VanAller Hernick, L., Landing, E. & Bartowski, K. E. Earth's oldest liverworts - *Metzgeriothallus sharonae* sp. nov. from the middle Devonian (Givetian) of eastern New York, USA. *Rev. Palaeobot. Palynol.* **148**, 154-162 (2008).
22. Heinrichs, J., Hentschel, J., Wilson, R., Feldberg, K. & Schneider, H. Evolution of leafy liverworts (Jungermanniidae, Marchantiophyta): estimating divergence times from chloroplast DNA sequences using penalized likelihood with integrated fossil evidence. *Taxon* **56**, 31-44 (2007).
23. Grolle, R. & Meister, K. *The Liverworts in Baltic and Bitterfeld Amber* (Weissdorn, Jena, 2004).
24. Grolle, R. & So, M. L. A fossil *Porella* (Porellaceae, Hepaticae) in Eocene Baltic amber. *Bot. J. Linn. Soc.* **145**, 485-488 (2004).
25. Grolle, R. Lebermoose im Bernstein 2. *Feddes Repert.* **91**, 401-407 (1980).
26. Caspary, R. Einige neue Pflanzenreste aus dem samländischen Bernstein. *Schriften der Königlichen Physikalisch-Ökonomischen Gesellschaft zu Königsberg* **27**, 1-8 (1886).
27. Hentschel, J., Schmidt, A. R. & Heinrichs, J. *Frullania cretacea* sp. nov. (Porellales, Jungermanniopsida), a leafy liverwort preserved in Cretaceous amber from Myanmar. *Cryptogam. Bryol.* **30**, 323-328 (2009).
28. Grolle, R. *Nipponolejeunea* fossil in Europa. *J. Hattori Bot. Lab.* 50, 143-157 (1981).
29. Grolle, R. Die Lebermoosgattung *Cheilolejeunea* fossil in Mitteleuropa. *Feddes Repert.* **95**, 229-236 (1984).
30. Gradstein, S. R. New fossil Hepaticae preserved in amber of the Dominican Republic. *Nova Hedwigia* **57**, 353-374 (1993).
31. Grolle, R. Fossil *Spruceanthus* in Europe and two other hepatics in Baltic amber. *Prace Mizeum Ziemi* **37**, 79-85 (1985).

32. Reiner-Drehwald, M. E., Schmidt, A. R. & Heinrichs, J. The genus *Lejeunea* in Miocene amber from the Dominican Republic. *Cryptog. Bryol.* **33**, 33-38.
33. Rikkinen, J. & Poinar Jr., G. O. A new species of *Phyllopsora* (Lecanorales, lichen-forming Ascomycota) from Dominican amber, with remarks on the fossil history of lichens. *J. Exp. Bot.* **59**, 1007-1011 (2008).
34. Grolle, R. Lebermoose im Bernstein 1. *Feddes Repert.* **91**, 183-190 (1980).
35. Grolle, R. & Heinrichs, J. Eocene *Plagiochila groehnii* sp. nov. – the first representative of Plagiochilaceae in Baltic amber. *Cryptogam. Bryol.* **24**, 289-293 (2003).
36. Grolle, R. Zwei weitere Lebermoose im Bernstein aus Bitterfeld (DDR). *Feddes Repert.* **96**, 41-46 (1985).
37. Grolle, R. *Lophozia kutscheri*, a new hepatic (Jungermanniales) in Bitterfeld amber from central Germany. *Bryologist* **107**,79-81 (2004).
38. Grolle, R. A fossil *Scapania* (Hepaticae) with perianth and capsule in bitterfeld amber (Eocene) from Germany. *Bryologist* **104**, 362-366 (2001).
39. Frahm, J.-P. Neue Moosfunde aus Baltischem Bernstein. *Limprichtia* **29**, 119-129 (2006).
40. Grimaldi, D. A., Engel, M. S. & Nascimbene, P. C. Fossiliferous Cretaceous amber from Myanmar (Burma): its rediscovery, biotic diversity, and paleontological significance. *Am. Mus. Novit.* **3361**, 1-72 (2002).
41. Shi, G., Grimaldi, D. A., Harlow, G. E., Wang, J., Wang, J., Yang, M., Lei, W., Li, Q. & Li, X. Age constraint on Burmese amber based on U-Pb dating of zirkons. *Cretaceous Research* **37**, 155–163 (2013).
42. Cruickshank, R. D. & Ko, K. Geology of an amber locality in the Hukawng valley, Northern Myanmar. *J. Asian Earth Sci.* **21**, 441-445 (2003).
43. Heinrichs, J., Reiner-Drehwald, M. E., Feldberg, K., von Konrat, M., Hentschel, J., Váňa, J., Nascimbene, P., Grimaldi, D. & Schmidt, A. R. The leafy liverwort *Frullania* in the Cretaceous amber forest of Myanmar. *Rev. Palaeobot. Palynol.* **169**, 21-28 (2012).
44. Ritzkowski, S. [Das geologische Alter der bernsteinführenden Sedimente in Sambia (Bezirk Kaliningrad),bei Bitterfeld (Sachsen-Anhalt) und bei Helmstedt (SE Niedersachsen)] *Investigations into Amber. Proceedings of the International Interdisciplinary Symposium: Baltic Amber and other Fossil Resins, 2–6 September 1997* [Kosmowska Ceranowicz, B. & Paner, H. (eds)] [33-38] (Archaeological Museum, Gdansk, 1999).
45. Standke, G. Bitterfelder Bernstein gleich Baltischer Bernstein? – Eine geologische Raum-Zeit-Betrachtung und genetische Schlußfolgerungen. *Exkurs.f. und Veröfftl. DGG* **236**, 11–33 (2008).
46. Blumenstengel, H. Zur Palynologie und Stratigraphie der Bitterfelder Bernsteinvorkommen (Tertiär). *Exkurs.f. und Veröfftl. DGG* **224**, 17 (2004).
47. Blumenstengel, H., Volland, L. & Krutzsch, W. *Zur Stratigraphie und Fazies des Tertiärs im Bitterfelder Raum – Referenzprofile tertiärer Ablagerungen in den Tagebauen Goitsche, Golpa-Nord und Göbern* (Geologisches Landesamt Sachsen-Anhalt, Halle, 1999).
48. Dunlop, J.A. [Bitterfeld amber] *Biodiversity of Fossils in Amber from the Major World Deposits* [Penney, D. (ed.)] [57-68] (Siri Scientific Press, Manchester, 2010).
49. Kreier, H. P., Feldberg, K., Mahr, F., Bombosch, A., Schmidt, A. R., Zhu, R.-L., von Konrat, M., Shaw, B., Shaw, A. J. & Heinrichs, J. Phylogeny of the leafy liverwort *Ptilidium*: cryptic speciation and shared haplotypes between the Northern and Southern Hemispheres. *Molec. Phylogen. Evol.* **57**, 1260-1267 (2010).
50. Devos, N., Renner, M. A. M., Gradstein, S. R., Shaw, J. & Vanderpoorten, A. Molecular data challenge traditional subgeneric divisions in the leafy liverwort *Radula*. *Taxon* **60**, 1623-1632 (2011).
51. Feldberg, K., Heinrichs, J., Schmidt, A. R., Váňa, J. & Schneider, H. Exploring the impact of fossil constraints on the divergence time estimates of derived liverworts. *Pl. Syst. Evol.* **299**, 585-601 (2013).
52. Schäfer-Verwimp, A., Feldberg, K., Dong, S., van Melick, H., Peralta, D.F., Schmidt, A. R., Schneider, H. & Heinrichs, J. Towards a monophyletic classification of Lejeuneaceae III: the systematic position of *Leiolejeunea*. *Phytotaxa* **170**, 187-198 (2014).
53. de Roo, R. T., Hedderson, T. A. & Söderström, L. Molecular insights into the phylogeny of the leafy liverwort family Lophoziaceae Cavers. *Taxon* **56**, 301-314 (2007).
54. Heinrichs, J., Bombosch, A., Feldberg, K., Kreier, H. P., Hentschel, J., Eckstein, J., Long, D., Zhu, R.-L., Schäfer-Verwimp, A., Schmidt, A. R., Shaw, B., Shaw, A. J. & Váňa, J. A phylogeny of the northern temperate leafy liverwort genus *Scapania* (Scapaniaceae, Jungermanniales). *Molec. Phylogen. Evol.* **62**, 973-985 (2012).
55. Iturralde-Vinent, M. A. & Mac Phee, R. D. E. Age and paleogeographical origin of Dominican amber. *Science* **273**, 1850–1852 (1996).
56. Heinrichs, J., Klugmann, F., Hentschel, J. & Schneider, H. DNA taxonomy, cryptic speciation and diversification of the Neotropical-African liverwort, *Marchesinia brachiata* (Lejeuneaceae, Porellales). *Molec. Phylogen. Evol.* **53**, 113-121 (2009).

57. Heinrichs, J., Schäfer-Verwimp, A., Boxberger, J., Feldberg, K., Solórzano Kramer, M.M. & Schmidt, A.R. A fossil species of *Ceratolejeunea* (Lejeuneaceae, Porellales) preserved in Miocene Mexican amber. *Bryologist* **117**, 10-14 (2014).

## Supplementary Information 4: Accession numbers

| Species | Voucher (Herbarium ) | Origin | GenBank Acc.-Nos. rbcL |
|---|---|---|---|
| **Outgroup: Marchantiopsida** | | | |
| *Asterella wallichiana* (Lehm. & Lindenb.) Grolle | Long 30251 (E) | Nepal | DQ286001 |
| *Blasia pusilla* L. | Tsubota 2538 (HIRO) | Japan | AB476555 |
| *Conocephalum japonicum* (Thunb.) Grolle | Long 30724 (E) | Nepal | DQ286005 |
| *Dumortiera hirsuta* (Sw.) Nees | Long et al. 29627 (E) | Mexico | DQ286009 |
| *Lunularia cruciata* (L.) Dumort. ex Lindb. | Long 29553 (E) | Mexico | DQ286011 |
| *Marchantia polymorpha* L. | Mishler 3783 (DUKE) | USA | U87079 |
| *Monoclea gottschei* Lindb. | Long et al. 29637 (E) | Mexico | DQ286016 |
| *Riccia fluitans* L. | Cady s.n. (E) | USA | DQ286023 |
| *Sphaerocarpos texanus* Austin | Stotler & Crandall-Stotler 1515 (ABSH) | USA | AY507425 |
| **Ingroup: Jungermanniopsida** | | | |
| **Pelliidae** | | | |
| *Allisonia cockaynii* (K.I.Goebel) R.M.Schust. | Stotler & Crandall-Stotler 4470 (ABSH) | New Zealand | AY507389 |
| *Calycularia crispula* Mitt. | Furuki 20741 (HIRO) | Japan | AB476556 |
| *Fossombronia australis* Mitt. | Stotler & Crandall-Stotler 4610 (ABSH) | New Zealand | AY507392 |
| *Fossombronia foveolata* Lindb. | D. & S. Williams s.n. (ABSH) | USA | AY507399 |
| *Fossombronia porphyrorhiza* (Nees) Prosk. | Gradstein 9831 (ABSH) | Brazil | AF536234 |
| *Greeneothallus gemmiparus* Hässel | Long 31831 (E) | Argentina | AY688780 |
| *Hattorianthus erimonus* (Steph.) R.M.Schust. & Inoue | Furuki s.n. (ABSH) | Japan | AY507403 |
| *Hymenophyton flabellatum* (Labill.) Dumort. | Stotler & Crandall-Stotler 4511 (ABSH) | New Zealand | AY507406 |
| *Jensenia spinosa* (Lindenb. & Gottsche) Grolle | Villarreal 793 (ABSH) | Venezuela | AY734689 |
| *Makinoa crispata* (Steph.) Miyake | Stotler & Crandall-Stotler 4047 (ABSH) | China | AY877390 |
| *Moerckia flotoviana* (Nees) Schiffn. | Kinser & Schuette 709 (ABSH) | USA | AY507413 |
| *Noteroclada confluens* Taylor | Weiss& Schwerdtfeger s.n. (ABSH) | Ecuador | HM005729 |
| *Pallavicinia lyellii* (Hook.) Gray | Marsh s.n. (ABSH) | USA | AY507416 |
| *Pellia epiphylla* (L.) Corda | Forrest et al. 700 (ABSH) | United Kingdom | HM005732 |
| *Petalophyllum ralfsii* (Wilson) Nees & Gottsche | J. Proskauer culture via D. Mueller-TAMU (in ABSH) | United Kingdom | AY507417 |
| *Phyllothallia nivicola* E.A.Hodgs. | Stotler & Crandall-Stotler 4537 (ABSH) | New Zealand | AY507418 |
| *Podomitrium phyllanthus* (Hook.) Mitt. | Stotler & Crandall-Stotler 4517 (ABSH) | New Zealand | AY507419 |
| *Symphyogyna hymenophyllum* (Hook.) Mont. & Nees | Stotler & Crandall-Stotler 4452 (ABSH) | New Zealand | AY507426 |
| *Symphyogyna undulata* Colenso | Stotler & Crandall-Stotler 4463 (ABSH) | New Zealand | AY688790 |
| *Symphyogynopsis filicum* (Nadeaud) Grolle | Tazawa 222 (HIRO) | Malaysia | AB476601 |
| *Xenothallus vulcanicolus* R.M.Schust. | Stotler & Crandall-Stotler 4580 (ABSH) | New Zealand | AY507431 |
| **Metzgeriidae** | | | |
| *Aneura pinguis* (L.) Dumort. | Yamaguchi 25538 (HIRO) | Japan | AB476550 |
| *Lobatiriccardia lobata* (Schiffn.) Furuki | Glenny & Kinser 4581 (ABSH) | New Zealand | AY507421 |
| *Metzgeria caledonica* Steph. | Oguri s.n. (HIRO) | New Caledonia | AB535638 |
| *Metzgeria consanguinea* Schiffn. | Masuzaki 542 (HIRO) | Japan | AB535640 |
| *Metzgeria fruticola* Spruce | Davis 361 (DUKE) | Ecuador | AY608035 |
| *Metzgeria furcata* (L.) Corda | Hentschel Bryo 01759 (GOET) | Germany | AM384875 |
| *Metzgeria pubescens* (Schrank) Raddi | Long 29523 (E) | United Kingdom | DQ268967 |
| *Mizutania riccardioides* Furuki & Z.Iwats. | Masuzaki 147 (HIRO) | Malaysia | AB476582 |
| *Pleurozia purpurea* Lindb. | Long 28868 (E) | Bhutan | AY877391 |
| *Riccardia capillacea* (Steph.) Meenks & C.DeJong | Forrest 558 (ABSH) | Venezuela | AY877392 |
| *Riccardia multifida* (L.) Gray | Forrest & Badcock 601 (ABSH) | Canada | DQ268980 |
| *Riccardia vitrea* Furuki | Furuki 19806 (HIRO) | Japan | AB476596 |
| *Verdoornia succulenta* R.M.Schust. | Stotler & Crandall-Stotler 4602 | New Zealand | AY507430 |

| | | | |
|---|---|---|---|
| | (ABSH) | | |
| **Jungermanniidae** | | | |
| *Acanthocoleus aberrans* (Lindenb. & Gottsche) Kruijt | Schäfer-Verwimp & Verwimp 4213 (GOET) | Spain | DQ983647 |
| *Acrobolbus ciliatus* (Mitt.) Schiffn. | Long 30514 (E) | Nepal | DQ026579 |
| *Acrolejeunea fertilis* (Reinw., Blume & Nees) Spruce | Gradstein et al. 10358 (GOET) | Malaysia | DQ983650 |
| *Acromastigum exiguum* (Steph.) A.Evans | NSW700366 (NSW) | Australia | EF100974 |
| *Acromastigum tenax* (Steph.) A.Evans | NSW701861 (NSW) | New Caledonia | EF100979 |
| *Adelanthus lindenbergianus* (Lehm.) Mitt. | Jacome JJ1045 (GOET) | Bolivia | GQ900284 |
| *Alobiella husnotii* Schiffn. | Schäfer-Verwimp & Verwimp 17800 (GOET) | Dominica | KC184698 |
| *Anastrophyllum bidens* (Reinw., Blume & Nees) Steph. | Gradstein 12067 (GOET) | Indonesia | KC184700 |
| *Anastrophyllum piligerum* (Reinw., Blume & Nees) Steph. | Schäfer-Verwimp et al. 24271 (GOET) | Ecuador | KC184704 |
| *Andrewsianthus perigonialis* (Hook. & Taylor) R.M.Schust. | Engel & von Konrat 27283 (GOET) | New Zealand | KC184707 |
| *Andrewsianthus australis* J.J.Engel | Schäfer-Verwimp & Verwimp 23734 (GOET) | Thailand | KC184706 |
| *Anoplolejeunea conferta* (C.F.W.Meissn. ex Spreng.) A.Evans | Wilson et al. 04-08 (GOET) | Ecuador | DQ983653 |
| *Anthelia julacea* (L.) Dumort. | Long 31292 (E) | Nepal | DQ026581 |
| *Archeophylla schusteri* (E.A.Hodgs. & Allison) R.M.Schust. | Glenny 9892b (H) | New Zealand | FJ173308 |
| *Archilejeunea parviflora* (Nees) Steph. | Gradstein & Dauphin 9370 (GOET) | Costa Rica | DQ983657 |
| *Ascidiota blepharophylla* subsp. *alaskana* Steere & R.M.Schust. | Lewis 514 (F) | USA | EF545280 |
| *Aureolejeunea fulva* R.M.Schust. | Costa & Gradstein 3849 (GOET) | Brazil | AY548083 |
| *Balantiopsis diplophylla* (Hook. f. & Taylor) Mitt. | Furuki 19645 (HIRO) | Australia | AB476553 |
| *Barbilophozia barbata* (Schreb.) Loeske | Hentschel Bryo 0753 (GOET) | Bulgaria | DQ312477 |
| *Barbilophozia hatcheri* (A.Evans) Loeske | Bakalin 53 (GOET) | Kamchatka | KC184708 |
| *Bazzania fasciculata* (Steph.) D.Meagher | NSW605694 (NSW) | Australia | EF100980 |
| *Bazzania franciana* (Steph.) N.Kitag. | NSW611491 (NSW) | New Caledonia | EF100981 |
| *Bazzania tricrenata* (Wahlenb.) Trevis. | Heinrichs et al. 4388 (GOET) | Austria | AY699990 |
| *Bazzania tridens* (Reinw., Blume & Nees) Trevis. | Masuzaki 304 (HIRO) | Japan | AB476554 |
| *Blepharolejeunea incongrua* (Lindenb. & Gottsche) Slageren & Kruijt | Schäfer-Verwimp & Verwimp 12800 (GOET) | Brazil | DQ983659 |
| *Brevianthus flavus* (Grolle) J.J.Engel & R.M.Schust. | Glenny 8918 (CHR) | New Zealand | DQ026582 |
| *Bryopteris diffusa* (Sw.) Nees | Acebey & Villavincencio 885 (GOET) | Bolivia | AY548085 |
| *Calypogeia integristipula* Steph. | Furuki 20768 (HIRO) | Japan | AB476557 |
| *Calypogeia muelleriana* (Schiffn.) K.Müller | Hentschel Bryo 01340 (GOET) | Germany | AM392302 |
| *Castanoclobos julaceus* (Hatcher ex J.J.Engel) J.J.Engel & Glenny | Glenny 10122 (CHR) | Australia | FJ173309 |
| *Caudalejeunea recurvistipula* (Gottsche) Schiffn. | Gradstein et al. 10378 (GOET) | Malaysia | DQ983661 |
| *Cephalozia bicuspidata* (L.) Dumort. | Hentschel Bryo 0362 (GOET) | Germany | AM392307 |
| *Cephalozia macgregorii* (Steph.) R.M.Schust. | Koponen 35296 (GOET) | Papua New Guinea | KC184732 |
| *Cephaloziella granatensis* (J.B.Jack) Fulford | Dauphin et al. 1548 (GOET) | Panama | KC184715 |
| *Cephaloziella microphylla* (Steph.) Douin | Schäfer-Verwimp & Verwimp 16291 (GOET) | Thailand | KC184716 |
| *Cephaloziella stellulifera* (Taylor) Schiffn. | Doyle 11250 (GOET) | USA | KC184718 |
| *Cephaloziopsis intertexta* (Gottsche) R.M.Schust. | Linneo et al. 424 (GOET) | Bolivia | KC184720 |
| *Ceratolejeunea cornuta* (Lindenb.) Schiffn. | Drehwald 4739 (GOET) | Bolivia | AY548088 |
| *Ceratolejeunea grandiloba* J.B.Jack & Steph. | Gradstein 7055 (GOET) | Bolivia | AY548090 |
| *Chaetophyllopsis whiteleggei* (Carrington & Pearson) R.M.Schust. | Curnow 4804 (GOET) | Australia | AY462292 |
| *Cheilolejeunea acutangula* (Nees) Grolle | Gradstein & Velasquez s.n. (GOET) | Mexico | DQ983664 |
| *Cheilolejeunea meyeniana* (Gottsche, Lindenb. & Nees) R.M.Schust. & Kachroo | Schäfer-Verwimp & Verwimp 24836/C (GOET) | Indonesia | DQ983666 |
| *Cheilolejeunea* spec. | Schäfer-Verwimp & Verwimp 24836/C (GOET) | Indonesia | KJ508344 |
| *Cheilolejeunea trifaria* (Reinw., Blume & Nees) Mizut. | Gradstein 9951A (GOET) | Bolivia | AY548093 |
| *Chiastocaulon dendroides* (Nees) Carl | Kurita 84 (HIRO) | Japan | AY699991 |
| *Chiloscyphus fragmentissimus* (R.M.Schust.) J.J.Engel & R.M.Schust. | Frahm 97/5/N (GOET) | Venezuela | DQ312480 |
| *Chiloscyphus fragrans* (Moris & De Not.) J.J.Engel & R.M.Schust. | Schwab 113 (JE) | Portugal | DQ312484 |
| *Chiloscyphus muricatus* (Lehm.) J.J.Engel & R.M.Schust. | Streimann 51629 (JE) | Australia | DQ312486 |
| *Chiloscyphus polyanthos* (L.) Corda | Hentschel Bryo 01758 (GOET) | Germany | AM392318 |

| | | | |
|---|---|---|---|
| *Chiloscyphus profundus* (Nees) J.J.Engel & R.M.Schust. | He Nygren & Piippo 1470 (H) | Finland | AY149852 |
| *Chiloscyphus sabuletorum* (Hook. f. & Taylor) J.J.Engel & R.M.Schust. | Busch et al. Bryo 01396 (JE) | Chile | DQ312488 |
| *Chiloscyphus semiteres* (Lehm.) Lehm. & Lindenb. | Streimann 58464 (GOET) | Australia | DQ312489 |
| *Cololejeunea laevigata* (Mitt.) R.M.Schust. | von Konrat 81-503 (GOET) | New Zealand | DQ238563 |
| *Cololejeunea obliqua* (Nees & Mont.) Schiffn. | Wilson et al. 04-11 (GOET) | Ecuador | DQ983669 |
| *Cololejeunea metzgeriopsis* (K.I.Goebel) Gradst. et al. | Ilkiu-Borges et al. 2946 (GOET) | Malaysia | DQ238567 |
| *Cololejeunea vitalana* Tixier | Schäfer-Verwimp & Holz SV/H-0473/A (GOET) | Costa Rica | DQ238564 |
| *Colura acroloba* (Mont. ex Steph.) Jovet-Ast | Pócs 03261/BK (GOET) | Republic of Fiji | DQ238565 |
| *Colura imperfecta* Steph. | Pócs 03261/BA (GOET) | Republic of Fiji | DQ238566 |
| *Colura tortifolia* (Mont. & Nees) Trevis. | Wilson et al. 04-12 (GOET) | Ecuador | DQ983671 |
| *Cryptolophocolea connata* (Sw.) L.Söderstr. & Váña | Gradstein 9404 (GOET) | Costa Rica | DQ312483 |
| *Cuspidatula monodon* Steph. | Pócs & Streimann 99189/B (EGR) | Australia | GQ900299 |
| *Cyclolejeunea peruviana* (Lehm. & Lindenb.) A.Evans | Gradstein 8546 (GOET) | Colombia | DQ983672 |
| *Cylindrocolea recurvifolia* (Steph.) Inoue | Deguchi 36641 (HIRO) | Japan | AM392306 |
| *Delavayella serrata* Steph. | Long 30388 (E) | Nepal | DQ026583 |
| *Dendrolejeunea fruticosa* (Lindenb. & Gottsche) Lacout. | Ilkiu-Borges & Yong 3026 (GOET) | Malaysia | DQ983673 |
| *Dendromastigophora flagellifera* (Hook.) R.M.Schust. | Glenny 8520 (CHR) | New Zealand | AY462294 |
| *Dicranolejeunea axillaris* (Nees & Mont.) Schiffn. | Drehwald 4829 (GOET) | Bolivia | DQ983674 |
| *Diplasiolejeunea cavifolia* Steph. | Schäfer-Verwimp & Verwimp 19036/A (GOET) | Malaysia | JQ729531 |
| *Diplasiolejeunea involuta* S.Winkl. ssp. *andicola* Pócs | Schäfer-Verwimp & Verwimp 23508 (GOET) | Ecuador | AY548096 |
| *Diplasiolejeunea villaumei* Steph. | Pócs et al. 04042/QR (EGR) | Kenya | JQ729467 |
| *Diplophyllum albicans* (L.) Dumort. | Hentschel Bryo 0240 (GOET) | Germany | AM392309 |
| *Diplophyllum taxifolium* (Wahlenb.) Dumort. | Hentschel Bryo 02044 (JE) | Germany | KJ508343 |
| *Douinia ovata* (Dicks.) H.Buch | Schofield & Davidson 1809 (GOET) | Canada | KC184724 |
| *Drepanolejeunea biocellata* A.Evans | Gradstein et al. 10053 (GOET) | Ecuador | AY548097 |
| *Drepanolejeunea erecta* (Steph.) Mizut. | Koponen et al. 50605 (H) | China | AY125940 |
| *Drepanolejeunea* spec. | Ilkiu-Borges et al. 3024 (GOET) | Malaysia | DQ983678 |
| *Drepanolejeunea vesiculosa* (Mitt.) Steph. | Gradstein et al. 10372 (GOET) | Malaysia | DQ983679 |
| *Drucella integristipula* (Steph.) E.A.Hodgs. | Brown 08/219 (NSW) | New Zealand | JF316287 |
| *Eocalypogeia quelpartensis* (S.Hatt. & Inoue) R.M.Schust. | Furuki 20233 (HIRO) | Japan | AB476566 |
| *Eotrichocolea polyacantha* (Hook. f. & Taylor) R.M.Schust. | Glenny 9891b (CHR) | New Zealand | FJ173322 |
| *Eremonotus myriocarpus* (Carrington) Pearson | Rothero NH 2116-2410 (GOET) | United Kingdom | EF503674 |
| *Evansiolejeunea roccatii* Vanden Berghen | Fischer RWA-1120 (GOET) | Rwanda | DQ983680 |
| *Frullania atrata* (Sw.) Dumort. | Costa & Gradstein 3859 (GOET) | Brazil | FJ380853 |
| *Frullania berthoumieuii* Steph. | Schäfer-Verwimp & Verwimp 16155 (GOET) | Thailand | FJ380724 |
| *Frullania depressa* Mitt. | Pócs et al. 9217/D (GOET) | Kenya | FJ380797 |
| *Frullania dilatata* (L.) Dumort. | Hentschel Bryo 0758 (GOET) | Bulgaria | AM384881 |
| *Frullania ecklonii* (Spreng.) Spreng. | Nöske 1705 (GOET) | Ecuador | FJ380785 |
| *Frullania glomerata* (Lehm. & Lindenb.) Nees & Mont. | Costa & Gradstein 3839 (GOET) | Brazil | FJ380774 |
| *Frullania gracilis* (Reinw., Blume & Nees) Dumort. | Schäfer-Verwimp & Verwimp 16158 (GOET) | Thailand | FJ380840 |
| *Frullania nodulosa* (Reinw., Nees & Blume) Nees | Pócs & Pócs 03271/B (GOET) | Republic of Fiji | FJ380814 |
| *Frullania obcordata* Lehm. & Lindenb. | Adolf s.n. (GOET) | Brazil | FJ380805 |
| *Frullania patula* Mitt. | von Konrat 98/43 (GOET) | New Zealand | FJ380728 |
| *Frullania ptychantha* Mont. | Engel & von Konrat 24353 (GOET) | New Zealand | FJ380807 |
| *Frullania reflexistipula* Sande Lac. | Lee & Indri 97 (GOET) | Indonesia | FJ380734 |
| *Frullania tamarisci* (L.) Dumort. | Hentschel Bryo 0733 (GOET) | Germany | AM384880 |
| *Frullanoides mexicana* Slageren | Burghardt 4421a (GOET) | Mexico | DQ983682 |
| *Fulfordianthus evansii* (Fulford) Gradst. | Dauphin s.n. (GOET) | Costa Rica | DQ983683 |
| *Fuscocephaloziopsis albescens* (Hook.) Váňa & L. Söderstr. | Schäfer-Verwimp & Verwimp 18194 (GOET) | Austria | KC184742 |
| *Fuscocephaloziopsis crassifolia* (Lindenb. & Gottsche) Váňa & L. Söderstr. | Churchill et al. 21621 (GOET) | Bolivia | KC184711 |
| *Gackstroemia magellanica* (Lam.) Trevis. | Hyvonen 3079 (H) | Argentina | AY462295 |
| *Geocalyx graveolens* (Schrad.) Nees | Meinunger s.n. (JE) | Germany | DQ312490 |
| *Goebeliella cornigera* (Mitt.) Steph. | Glenny 2001 (H) | New Zealand | AY462296 |
| *Goebelobryum unguiculatum* (Hook. f. & Taylor) Grolle | Qiu et al. NZ-03054 (MICH) | New Zealand | DQ787465 |
| *Gongylanthus ericetorum* (Raddi) Nees | Duell 63.(35.) (GOET) | Greece | AM392303 |
| *Gottschelia schizopleura* (Spruce) Grolle | Ah-Peng R96 (GOET) | Madagascar | FJ984940 |
| *Gymnomitrion concinnatum* (Lightf.) Corda | Davis 424 (DUKE) | Canada | DQ439686 |

| | | | |
|---|---|---|---|
| *Gyrothyra underwoodiana* M.Howe | Forrest 593 (ABSH) | Canada | DQ268970 |
| *Harpalejeunea grandistipula* R.M.Schust. | Schäfer-Verwimp et al. 24289 (GOET) | Ecuador | DQ983685 |
| *Harpanthus scutatus* (F.Weber & D.Mohr) Spruce | Risk et al. 10341 (DUKE) | USA | DQ787464 |
| *Herbertus sendtneri* (Nees) A.Evans | Groth s.n. (GOET) | Bolivia | AY699993 |
| *Herzogianthus vaginatus* (Herzog) R.M.Schust. | Qiu & Braggins NZ-03173 (MICH) | New Zealand | DQ787462 |
| *Heteroscyphus aselliformis* (Reinw., Blume & Nees) Schiffn. | Gradstein 10240 (GOET) | Indonesia | DQ312493 |
| *Heteroscyphus coalitus* (Hook.) Schiffn. | Long 30316 (H) | Nepal | AY149844 |
| *Heteroscyphus knightii* (Steph.) Grolle | Engel & vonKonrat 28206 (H) | New Zealand | HM439103 |
| *Hyalolepidozia microphylla* R.M.Schust. ex J.J.Engel | Brown 08/301 (NSW) | New Zealand | JF316304 |
| *Hygrolembidium acrocladum* (Berggr.) R.M.Schust. | Streimann 16733 (H) | Australia | AY462301 |
| *Isolembidium anomalum* (Rodway) Grolle | Brown 08/286 (NSW) | New Zealand | JF316294 |
| *Isotachis japonica* Steph. | Matsuda 52 (HIRO) | Japan | AB476569 |
| *Jackiella curvata* E.A.Hodgs. & Allison | Qiu et al. NZ-03064 (AK, MICH) | New Zealand | DQ787461 |
| *Jubula hutchinsiae* (Hook.) Dumort. | Drehwald 3007 (GOET) | Portugal | AY548101 |
| *Jungermannia atrovirens* Dumort. | Hentschel & Heinrichs Bryo 01784 (GOET) | Germany | EF503675 |
| *Jungermannia exsertifolia* Steph. ssp. *cordifolia* Váňa | Duncan s.n. (GOET) | USA | EF503676 |
| *Jungermannia ovato-trigona* (Steph.) Grolle | Gradstein 10007 (GOET) | Colombia | DQ312500 |
| *Jungermannia pumila* With. | Bakalin P-72-2-05 (GOET) | Russia | EF503677 |
| *Kurzia hippuroides* (Hook. f. & Taylor) Grolle | NSW709903 (NSW) | Australia | EF100997 |
| *Kurzia trichoclados* (Müll. Frib.) Grolle | Rothero 11014 (E) | United Kingdom | JF316319 |
| *Kymatocalyx dominicensis* (Spruce) Váňa | Schäfer-Verwimp & Verwimp 22451 (GOET) | Guadeloupe | KC184726 |
| *Leiocolea heterocolpos* (Thed.) H.Buch | Stotler & Crandall-Stotler 4357 (ABSH) | USA | DQ268972 |
| *Leiomitra lanata* (Hook.) R.M.Schust. | Glenny 8521 (CHR) | New Zealand | AY462305 |
| *Lejeunea cancellata* Nees & Mont. ex Mont. | Wilson et al. 04-02 (GOET) | Ecuador | DQ983686 |
| *Lejeunea catinulifera* Spruce | Wilson et al. 04-01 (GOET) | Ecuador | DQ983687 |
| *Lejeunea cavifolia* (Ehrh.) Lindb. | Heinrichs 3695 (GOET) | Germany | AY548102 |
| *Lejeunea eckloniana* Lindenb. | Schäfer-Verwimp & Verwimp 24788 (GOET) | Spain | DQ983690 |
| *Lejeunea flava* (Sw.) Nees | Gradstein s.n. (GOET) | Brazil | DQ983692 |
| *Lejeunea laetevirens* Nees & Mont. | Schäfer-Verwimp & Verwimp 17899 (GOET) | Dominica | AY548103 |
| *Lejeunea mimula* Hürl. | Schäfer-Verwimp & Verwimp 20930 (GOET) | Indonesia | AY548104 |
| *Lejeunea paucidentata* (Steph.) Grolle | Schäfer-Verwimp & Verwimp 17737 (GOET) | Dominica | DQ983695 |
| *Lembidium nutans* (Hook. f. & Taylor) Mitt. ex A.Evans | NSW493343 (NSW) | New Zealand | EF100992 |
| *Lepicolea attenuata* (Mitt.) Steph. | Stotler & Crandall-Stotler 4586 (ABSH) | New Zealand | AY507410 |
| *Lepicolea pruinosa* (Taylor) Spruce | Schäfer-Verwimp et al. 24205 (GOET) | Ecuador | AM392314 |
| *Lepidogyna hodgsoniae* (Grolle) R.M.Schust. | Frey & Pfeiffer 98-T 68 B (BSB) | New Zealand | DQ645968 |
| *Lepidolaena clavigera* (Hook.) Dumort. ex Trevis. | Curnow 5079 (H) | New Zealand | AY462309 |
| *Lepidolejeunea eluta* (Nees) R.M.Schust. | Churchill & Vasquez 2180 (GOET) | Bolivia | AY548066 |
| *Lepidozia glaucophylla* (Hook. f. & Taylor) Gottsche, Lindenb. & Nees | NSW722420 (NSW) | Australia | EF100999 |
| *Lepidozia spinosissima* (Hook. f. & Taylor) Mitt. | NSW712837 (NSW) | New Zealand | EF101002 |
| *Lepidozia ulothrix* (Schwägr.) Lindenb. | NSW708080 (NSW) | New Zealand | EF101004 |
| *Leptolejeunea elliptica* (Lehm. & Lindenb.) Schiffn. | Matsuda 30 (HIRO) | Japan | AB476575 |
| *Leptoscyphus autoicus* (J.J.Engel & Gradst.) Vanderp. & Gradst. | Gradstein & Nöske 10090 (GOET) | Ecuador | DQ312511 |
| *Leptoscyphus jackii* (Steph.) Grolle | Schäfer-Verwimp et al. 24296 (GOET) | Ecuador | DQ312504 |
| *Lethocolea naruto-toganensis* Furuki | Furuki 18022 (HIRO) | Japan | AB476576 |
| *Cheilolejeunea xanthocarpa* (Lehm. & Lindenb.) Malombe | Costa & Gradstein 3839 (GOET) | Brazil | DQ983700 |
| *Lindigianthus cipaconeus* (Gottsche) Kruijt & Gradst. | Kottke & Preußing MPEO2390 (GOET) | Ecuador | DQ983702 |
| *Liochlaena lanceolata* Nees | Stotler & Crandall-Stotler 107 (ABSH) | USA | AY507409 |
| *Lopholejeunea eulopha* (Taylor) Schiffn. | Pócs & Streimann 9987/H (GOET) | Australia | AY548067 |
| *Lopholejeunea subfusca* (Nees) Schiffn. | Gradstein et al. 10368 (GOET) | Malaysia | DQ983705 |
| *Lophozia ascendens* (Warnst.) R.M.Schust. | Klama et al. 171 (GOET) | Poland | KC184727 |
| *Lophozia lantratoviae* Bakalin | Bakalin P-72-19-05 (GOET) | Russia | KC184728 |
| *Lophoziopsis excisa* (Dicks.) Konstant. & Vilnet | Bakalin P-74-17-05 (GOET) | Russia | KC184729 |

| | | | |
|---|---|---|---|
| *Lophoziopsis longidens* (Lindb.) Konstant. & Vilnet | Bakalin 65 (GOET) | Russia | KC184730 |
| *Macrocolura sagittistipula* (Spruce) R.M.Schust. | Schäfer-Verwimp & Verwimp 18014 (GOET) | Dominica | DQ983707 |
| *Marchesinia robusta* (Mitt.) Schiffn. | Wilson et al. 04-05 (GOET) | Ecuador | DQ983710 |
| *Marsupella emarginata* (Ehrh.) Dumort. | Qiu 99077 (Z) | ? | DQ645972 |
| *Marsupidium latifolium* R.M.Schust. | Dauphin 2920 (NY) | Costa Rica | AY608034 |
| *Mastigolejeunea auriculata* (Wilson & Hook.) Schiffn. | Schäfer-Verwimp & Verwimp 20780 (GOET) | Indonesia | AY548071 |
| *Mastigophora diclados* (Brid. ex F.Weber) Nees | Schäfer-Verwimp & Verwimp24898 (GOET) | Indonesia | EF503679 |
| *Metacalypogeia cordifolia* (Steph.) Inoue | Furuki 21344 (HIRO) | Japan | AB476580 |
| *Mnioloma fuscum* (Lehm.) R.M.Schust. | Masuzaki 918 (HIRO) | Malaysia | AB476585 |
| *Mylia verrucosa* Lindb. | Masuzaki 1344 (HIRO) | Japan | AB476587 |
| *Myriocolea irrorata* Spruce | Gradstein et al. 10033B (GOET) | Ecuador | AY548073 |
| *Myriocoleopsis gymnocolea* (Spruce) M.E.Reiner & Gradst. | Gradstein et al. 10020 (GOET) | Ecuador | DQ238568 |
| *Nardia succulenta* (Rich. ex Lehm. & Lindenb.) Spreng. | Churchill et al. 22796 (GOET) | Bolivia | EF503681 |
| *Neesioscyphus bicuspidatus* (Steph.) Grolle | Dauphin 2042 (H) | Costa Rica | DQ354978 |
| *Neoorthocaulis floerkei* (F.Weber & D.Mohr) L.Söderstr., De Roo & Hedd. | Drehwald s.n. (GOET) | Germany | KC184734 |
| *Neotrichocolea bisseti* (Mitt.) S.Hatt. | He 040522 (PE) | China | EF460702 |
| *Neurolejeunea breutelii* (Gottsche) A.Evans | Gradstein & Costa 3871 (GOET) | Brazil | DQ983713 |
| *Nipponolejeunea subalpina* (Horik.) S.Hatt. | Ohnishi 5611 (HIRO) | Japan | AM392292 |
| *Nothogymnomitrion erosum* (Carrington & Pearson) R.M.Schust. | Streimann 53475 (JE) | Australia | GQ900318 |
| *Notoscyphus lutescens* (Lehm. & Lindenb.) Mitt. | Pócs et al. 98101/D (GOET) | Vietnam | EF503682 |
| *Nowellia dominicensis* Steph. | Schäfer-Verwimp & Verwimp 17954/A (GOET) | Dominica | KC184735 |
| *Odontolejeunea lunulata* (F. Weber) Schiffn. | Piippo s.n. (H) | Costa Rica | AY302456 |
| *Odontoschisma falcifolium* Steph. | Gradstein 8535 (GOET) | Colombia | KC184739 |
| *Odontoschisma fluitans* (Nees) L.Söderstr. & Váňa | Heinrichs et al. 2058 (GOET) | Germany | KC184721 |
| *Omphalanthus filiformis* (Sw.) Nees | Drehwald 4462 (GOET) | Bolivia | AY548074 |
| *Pedinophyllum interruptum* (Nees) Kaal. | Heinrichs et al. 4701 (GOET) | Germany | GU593095 |
| *Phaeolejeunea amicorum* (Hürl.) Pócs | Pócs 03304L (EGR) | Republic of Fiji | DQ983718 |
| *Physantholejeunea portoricensis* (Hampe & Gottsche) R.M.Schust. | Schäfer-Verwimp & Verwimp 22615 (GOET) | Guadeloupe | DQ983719 |
| *Plagiochila alternans* Lindenb. & Gottsche | Heinrichs et al. 4178 (GOET) | Bolivia | AY699995 |
| *Plagiochila asplenioides* (L.) Dumort. | Heinrichs & Groth 4339 (GOET) | Germany | AY699996 |
| *Plagiochila cucullifolia* J.B.Jack & Steph. | Holz 558 E/5-01 | Ecuador | DQ194126 |
| *Plagiochila diversifolia* Lindenb. & Gottsche | Holz 017 E/5-01 | Ecuador | DQ194131 |
| *Plagiochila dura* De Not. | Drehwald 3190 (GOET) | Chile | DQ194133 |
| *Plagiochila dusenii* Steph. | Holz 146 (GOET) | Argentina | DQ194135 |
| *Plagiochila frondescens* (Nees) Lindenb. | Schäfer-Verwimp & Verwimp 20704 (GOET) | Indonesia | DQ194141 |
| *Plagiochila fruticosa* Mitt. | Deguchi s.n. (GOET) | Japan | DQ194142 |
| *Plagiochila fusca* Sande Lac. | Gradstein 10259 (GOET) | Indonesia | DQ194143 |
| *Plagiochila integerrima* Steph. | O'Shea M7552a (GOET) | Malawi | DQ194095 |
| *Plagiochila montagnei* Nees | Groth s.n. (GOET) | Bolivia | DQ194164 |
| *Plagiochila peculiaris* Schiffn. | Long 28832 (E) | Bhutan | DQ194176 |
| *Plagiochila punctata* (Taylor) Taylor | Rycroft 01013 (GOET) | United Kingdom | DQ194180 |
| *Plagiochila rubescens* (Lehm. & Lindenb.) Lindenb. | Rycroft 020723-6 (GOET) | Chile | DQ194187 |
| *Plagiochila rutilans* Lindenb. | Groth 101 (GOET) | Bolivia | DQ194189 |
| *Plagiochila sandei* Dozy ex Sande Lac. | Gradstein 9970 (GOET) | Indonesia | DQ194191 |
| *Plagiochila sciophila* Nees | Ohnishi 5400 (HIRO) | Japan | DQ194193 |
| *Plagiochila semidecurrens* (Lehm. & Lindenb.) Lehm. & Lindenb. | Long 21348 (E) | Nepal | DQ194194 |
| *Plagiochilion mayebarae* S.Hatt. | Onishi 5588 (HIRO) | Japan | AY699999 |
| *Microlejeunea squarrosa* (Steph.) Heinrichs, Schäf.-Verw., Pócs & S.Dong | Schäfer-Verwimp & Verwimp 13376 (GOET) | Brazil | DQ983720 |
| *Porella cordaeana* (Huebener) Moore | Long 34092 (E) | United Kingdom | EF545286 |
| *Porella nitens* (Steph.) S.Hatt. | Long 34415 (E) | China | EF545339 |
| *Porella obtusata* (Taylor) Trevis. | Schäfer-Verwimp & Verwimp 24754 (GOET) | Spain | EF545349 |
| *Pseudocephalozia lepidozioides* R.M.Schust. | Brown 08/216 (NSW) | New Zealand | JF316289 |
| *Pseudomarsupidium decipiens* (Hook.) Grolle | Wigginton 05/613 (GOET) | United Kingdom | FJ984934 |

| | | | |
|---|---|---|---|
| *Psiloclada clandestina* Mitt. | NSW611373 (NSW) | New Caledonia | EF101007 |
| *Ptilidium ciliare* (L.) Hampe | Hentschel Bryo 01602 (GOET) | Germany | AM392297 |
| *Ptychanthus striatus* (Lehm. & Lindenb.) Nees | Deguchi 36758 (GOET) | Japan | DQ983722 |
| *Pycnolejeunea densistipula* (Lehm. & Lindenb.) Steph. | Schäfer-Verwimp 23368 (GOET) | Ecuador | AY548075 |
| *Radula complanata* (L.) Dumort. | Heinrichs 1712 (GOET) | Austria | KJ508346 |
| *Radula frondescens* Steph. | Holz CR 00-774B (GOET) | Costa Rica | AM392291 |
| *Radula javanica* Gottsche | Holz FG 00-62 (GOET) | French Guiana | AM392290 |
| *Radula kojana* Steph. | Tsubota 5932 (HIRO) | Japan | AB476595 |
| *Radula striata* Mitt. ex Steph. | Drehwald DB34326 (GOET) | Chile | KJ508345 |
| *Rectolejeunea berteroana* (Gottsche ex Steph.) A.Evans | Schäfer-Verwimp & Verwimp 22245/A (GOET) | Guadeloupe | DQ983724 |
| *Saccogyna viticulosa* (L.) Dumort. | Gradstein 9977 (GOET) | Spain | DQ312514 |
| *Saccogynidium goebelii* (Herzog) Grolle | Schäfer-Verwimp & Verwimp 24874 (GOET) | Indonesia | EF503683 |
| *Scapania ampliata* Steph. | Yokoyama 11576 (GOET) | Japan | KC184744 |
| *Scapania aspera* M.Bernet & Bernet | Hentschel Bryo 0762 (GOET) | Bulgaria | AM392310 |
| *Scapania javanica* Gottsche | Schäfer-Verwimp & Verwimp 16900 (GOET) | Indonesia | KC184750 |
| *Scapania paludosa* (Müll. Frib.) Müll.Frib. | Schäfer-Verwimp & Verwimp 19613 (GOET) | Germany | KC184753 |
| *Scapania portoricensis* Hampe & Gottsche | Churchill 24297 (GOET) | Ecuador | KC184754 |
| *Schiffneria hyalina* Steph. | Schäfer-Verwimp & Verwimp 24869 (GOET) | Indonesia | KC184759 |
| *Schiffneriolejeunea parviloba* (Steph.) Gradst. | Schäfer-Verwimp & Verwimp 20314 (GOET) | Réunion | AY548076 |
| *Schistochila acuminata* Steph. | Schäfer-Verwimp & Verwimp 25281 (GOET) | Indonesia | AM392299 |
| *Schistochila aligera* (Nees & Blume) J.B.Jack & Steph. | Gradstein 10406 (GOET) | Malaysia | AM392300 |
| *Schistochilopsis incisa* (Schrad.) Konstant. | Hentschel & Wilson Bryo 01593 (GOET) | Germany | AM392308 |
| *Siphonolejeunea elegantissima* (Steph.) Grolle | Pócs & Brown 0026/A,A (EGER) | Australia | DQ983726 |
| *Solenostoma infusca* (Mitt.) Hentschel | Deguchi s.n. (GOET) | Japan | DQ312498 |
| *Sphenolobus minutus* (Schreb.) Berggr. | Hentschel Bryo 0421 (GOET) | Norway | DQ312475 |
| *Spruceanthus theobromae* (Spruce) Gradst. | Andersson & Kautz AK10 (GOET) | Ecuador | DQ983727 |
| *Stictolejeunea squamata* (Willd. ex F.Weber) Schiffn. | Dauphin 2134 (GOET) | Costa Rica | DQ983728 |
| *Symbiezidium barbiflorum* (Lindenb. & Gottsche) A.Evans | Gradstein & Dauphin 9363 (GOET) | Costa Rica | DQ983729 |
| *Symbiezidium transversale* (Sw.) Trevis. | Gradstein 9924 (GOET) | Bolivia | AY548079 |
| *Syzygiella anomala* (Lindenb. & Gottsche) Steph. | Heinrichs et al. K4 (GOET) | Ecuador | GQ900328 |
| *Syzygiella perfoliata* (Sw.) Spruce | Gradstein 3888 (GOET) | Brazil | GQ900353 |
| *Telaranea wallichiana* (Gottsche) R.M.Schust. | Brown 04/829 (NSW) | Papua New Guinea | JF316328 |
| *Temnoma pilosum* (A.Evans) R.M.Schust. | Hyvoenen 5084 (H) | Chile | AY462330 |
| *Tetracymbaliella cymbalifera* (Hook. f. & Taylor) Grolle | Pócs et al. 90066/F (H) | Tanzania | DQ026591 |
| *Tetralophozia pilifera* (Steph.) R.M.Schust. | Gradstein 11015 (GOET) | Indonesia | KC184763 |
| *Thysananthus comosus* Lindenb. ex Lehm. | Gradstein et al. 10366 (GOET) | Malaysia | DQ983736 |
| *Triandrophyllum subtrifidum* (Hook. & Taylor) Fulford & Hatcher | Schäfer-Verwimp et al. 24436 (GOET) | Ecuador | AM392315 |
| *Trichocolea pluma* Dumort. | Masuzaki 776 (HIRO) | Japan | AB476603 |
| *Trichocoleopsis sacculata* (Mitt.) S.Okamura | Liu 4170 (PE) | China | EF460701 |
| *Tricholepidozia plumulosa* (Lehm. & Lindenb.) E.D.Cooper | Engel 26422 (F) | Chile | JF316312 |
| *Trichotemnoma currugatum* (Steph.) R.M.Schust. | Glenny 8426 (CHR) | New Zealand | AY462333 |
| *Tritomaria exsecta* (Schmidel ex Schrad.) Schiffn. ex Loeske | Schäfer-Verwimp & Verwimp 21816 (GOET) | Russia | KC184765 |
| *Tylimanthus laxus* (Lindenb.) Steph. | Holz CR-00-0197 (GOET) | Costa Rica | AY700004 |
| *Acrobolbus saccatus* (Hook.) Trevis. | Qiu 02044; Frey & Pfeiffer 98-Mo 41 A (BSB) | New Zealand | DQ645975 |
| *Vetaforma dusenii* (Steph.) Fulford & J.Taylor | Engel 11423 (C) | Chile | AY462335 |
| *Wettsteinia inversa* (Sande Lac.) Schiffn. | Gradstein 11014 (GOET) | Indonesia | FJ984935 |
| *Xylolejeunea crenata* (Mont.) X.-L.He & Grolle | Schäfer-Verwimp & Verwimp 11225 (GOET) | Brazil | DQ983740 |
| *Zoopsidella caledonica* (Steph.) R.M.Schust. | NSW611654 (NSW) | New Caledonia | EF101022 |
| *Zoopsis leitgebiana* (Carrington & Pearson) Bastow | NSW614795 (NSW) | Australia | EF101023 |
